# Bifunctional Skipped Dienes through Cu/Pd-Catalyzed Allylboration of Alkynes with B_2_pin_2_ and Vinyl Epoxides

**DOI:** 10.1021/acs.orglett.2c03390

**Published:** 2022-11-03

**Authors:** Nuria Vázquez-Galiñanes, Isabel Velo-Heleno, Martín Fañanás-Mastral

**Affiliations:** Centro Singular de Investigación en Química Biolóxica e Materiais Moleculares (CiQUS), Universidade de Santiago de Compostela, 15782 Santiago de Compostela, Spain

## Abstract

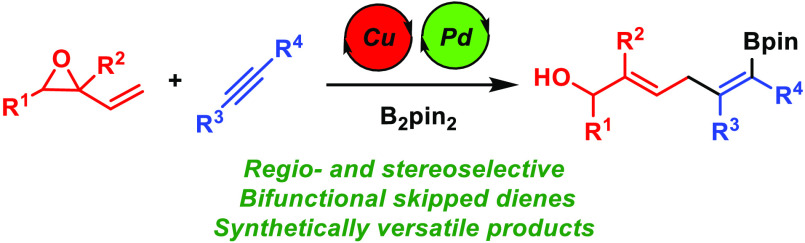

A method for the use of vinyl epoxides in catalytic allylboration of alkynes is described. This transformation allows for the synthesis of bifunctional skipped dienes bearing both an allylic alcohol and an alkenylboronate from simple starting materials with high regio- and stereoselectivity. These two functionalities provide these products with highly versatile reactivity, as shown by their stereocontrolled conversion into cyclic boron compounds and polyenes.

Synthetic methods that enable the assembly of complex organic molecules from simple and readily available starting materials are highly sought. In particular, multicomponent reactions that provide stereoselective access to densely functionalized building blocks still represent a formidable challenge and are particularly valuable to achieve molecular diversity through diverse synthetic modifications.^1^

Vinyl epoxides are a versatile class of substrates since they can engage in a number of synthetic transformations.^2^ The metal-catalyzed allylic alkylation of vinyl epoxides is of main interest since it allows the concomitant formation of a C–C bond and an allylic alcohol, which can be used for further functionalization. Although several carbon nucleophiles have been used in this reaction,^3^ examples regarding the use of alkenyl nucleophiles are mainly limited to couplings with alkenylstannanes^4^ and alkenylboranes (Scheme 1A).^5^ A similar type of products can be accessed by the Ni-catalyzed three-component coupling of a vinyl epoxide, an alkyne, and dimethylzinc (Scheme 1B).^6^ However, besides the drawbacks associated with the stoichiometric use of organometallic reagents, control over the regioselectivity (1,4- vs 1,2-addition) and stereoselectivity (*E* vs *Z* isomer) has represented a major issue in both cases.

**Scheme 1:**
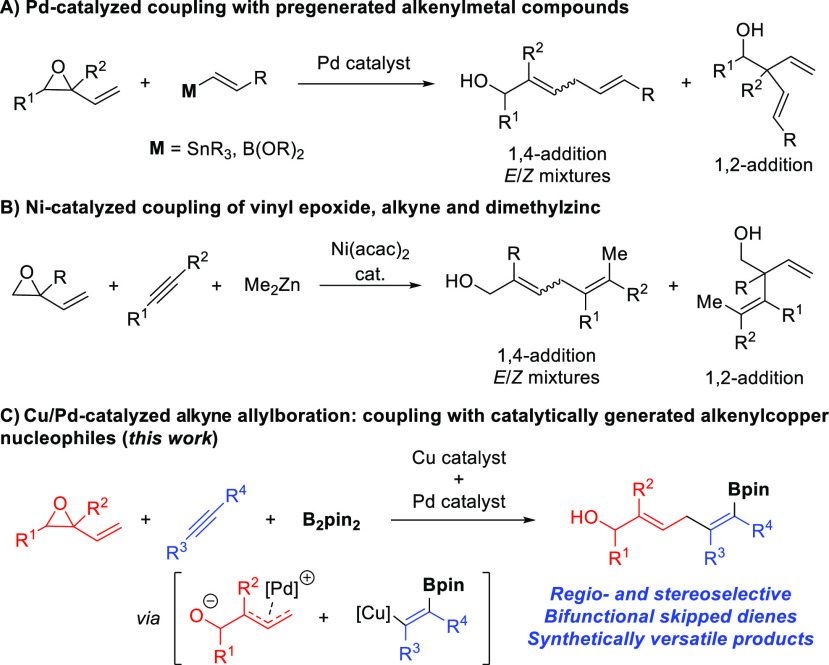
Methods for the Alkenylation of Vinyl Epoxides

In recent years, our group^7^ and that of Gong and Fu^8^ have explored a complementary strategy toward Pd-catalyzed stereoselective allylic alkenylation. This approach is based on a synergistic catalytic mechanism that involves the generation of a catalytic stereodefined C(sp^2^) nucleophile by LCu–Bpin addition across an alkyne followed by Pd-catalyzed allylic substitution to formally provide a carboboration product.^9^ This process is attractive since a simple alkyne is used as a pronucleophile, thus allowing the concentration of the reactive species to be kept low, and since two new C–C and C–B bonds are generated in a single operation.

Based on our previous studies, we envisioned that the allylboration of an alkyne using a vinyl epoxide as the allylic component would result in a bifunctional skipped diene bearing two orthogonal functionalities such as an allylic alcohol and an alkenylboronate (Scheme 1C). Given the broad reactivity of both functionalities, this transformation would provide a highly versatile building block. Besides the control over the regio- and stereoselectivity, the success of our proposed strategy also requires a high level of chemoselectivity since competitive addition of B_2_pin_2_ to the vinyl epoxide^10^ must be suppressed. Moreover, trapping of the allylpalladium complex by the alkenylcopper intermediate should be faster than potentially competitive rearrangement to the corresponding carbonyl compound.^11^ Herein we report the successful implementation of this idea and thus the development of a three-component catalytic process that allows for the regio-, stereo-, and chemoselective synthesis of bifunctional skipped dienes. This method is distinct from previous hydrocarbon carboboration reactions since it is the first example that allows for the use of vinyl epoxides. It is also important to note that this new methodology provides unique access to these bifunctional dienol boronates, which can be transformed into a variety of structures in a stereocontrolled manner.

We started our studies by applying our previously described conditions for the Cu/Pd-catalyzed allylboration of alkynes with allyl carbonates^7a^ to the reaction involving 1,3-butadiene epoxide (**1**), 1-phenyl-1-propyne (**2**), and B_2_pin_2_, (Table 1). The initial experiment already showed the challenging nature of this multicomponent reaction since diene **3** was obtained in only 10% yield as a 1:1 mixture of *E*,*Z* and *Z*,*Z* isomers (entry 1). Interestingly, although **3** was obtained in similar yield when reaction was run at 30 °C, it was obtained as the pure (*Z*)-alkenyl boronate and (*E*)-allylic alcohol (entry 2). Increasing the Cu/Pd molar ratio from 1:1 to 2:1 produced a slight improvement, although the yield of **3** was still far from satisfactory (entry 3). Careful analysis of the reaction mixtures revealed that the low yield of **3** was accompanied by total consumption of **1**, which likely occurred through direct borylation pathways.^12^ In order to minimize these nonproductive reactions, we reasoned that keeping a lower concentration of **1** in the reaction medium should result in the formation of a larger amount of the desired diene **3**. Accordingly, we found that slow addition and adjustment of the vinyl epoxide stoichiometry produced a significant enhancement of the reaction yield (entries 4 and 5). Remarkably, this transformation could be successfully carried out by using a catalytic amount of NaO^*t*^Bu, which even improved the efficiency of the reaction. Under these optimized conditions, bifunctional diene **3** was obtained as a single 1,4-addition product in 70% yield with complete control over the stereoselectivity of both newly formed double bonds (entry 6). Evaluation of other solvents, copper and palladium catalysts, and bases did not result in any improvement (see the Supporting Information). Finally, control experiments demonstrated that both copper and palladium are essential to ensure product formation (entries 7 and 8).

**Table 1:**
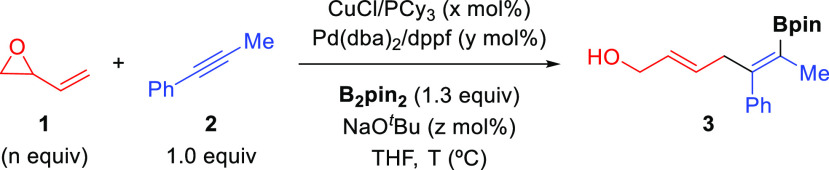
Optimization Studies^a^

entry	equiv of **1**	*T* (°C)	*x* mol %	*y* mol %	*z* mol %	conv. (%)	yield of **3** (%)^b^
1	1.2	50	5	5	200	54	10^c^
2	1.2	30	5	5	200	94	15
3	1.2	30	10	5	200	90	21
4^d^	1.2	30	10	5	200	95	57
5^d^	2	30	10	5	200	90	70
6^d^	2	30	10	5	20	100	78 (70^e^)
7	1.2	30	–	5	200	30	–
8	1.2	30	5	–	200	70	–

a Reactions were performed on a 0.3 mmol scale (0.1 M). **3** was obtained as a single isomer unless otherwise noted.

b Determined by ^1^H NMR analysis using 1,3,5-trimethoxybenzene as an internal standard.

c 1:1 mixture of *E*,*Z* and *Z*,*Z* isomers.

d **1** in THF (0.5 mL) was added over 1 h by syringe pump.

e The yield of the isolated product shown in parentheses.

Having established the optimized conditions (Table 1, entry 6), we set out to investigate the scope of the reaction (Scheme 2). Remarkably, the reaction proceeded with total stereo- and regioselectivity and furnished exclusively the 1,4-addition product with excellent *E*,*Z* selectivity in nearly all cases. Internal aryl alkyl alkynes and 1,2-diarylalkynes proved to be efficient substrates and reacted with epoxide **1** and B_2_pin_2_ to afford the corresponding bifunctional dienes **3**–**6** in good yields. A terminal alkyne such as trimethylsilylacetylene also worked well in this transformation, providing trifunctionalized skipped diene **7** in 56% yield. However, the use of other terminal alkynes such as 1-hexyne or phenylacetylene was problematic and resulted in no product formation.

**Scheme 2:**
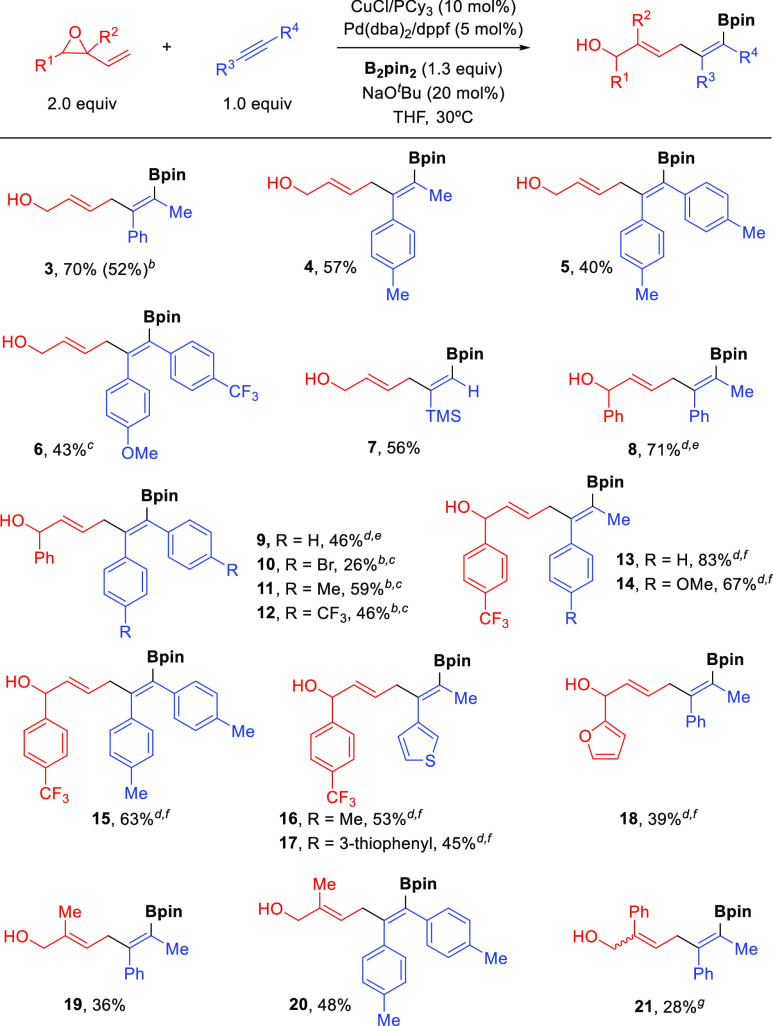
Cu/Pd-Catalyzed Alkyne Allylboration with Vinyl Epoxides and B_2_pin_2_ Conditions: see Table 1, entry 6. Yields of isolated products are reported. The reaction was run on a 1 mmol scale. Obtained as a 2:1 mixture of regioisomers (only the major isomer is shown). The reaction was run at 50 °C. The vinyl epoxide was used as a 2:1 *cis*/*trans* mixture. The vinyl epoxide was used as a 5:1 *cis*/*trans* mixture. Obtained as a 1:1 *E*,*Z*/*Z*,*Z* mixture.

More substituted vinyl epoxides were also evaluated under these dual Cu/Pd catalytic conditions. 1,2-Disubstituted epoxides required a slightly higher temperature but also worked well and afforded products **8**–**15** in moderate to good yields. Notably, these secondary allylic alcohols were obtained with perfect regio- and stereoselectivity despite the use of diastereomeric *cis*/*trans* mixtures of the corresponding starting vinyl epoxides.^13,14^ Heteroaromatic substituents were well-tolerated at either the alkyne (**16** and **17**) or the vinyl epoxide structure (**18**). Isoprene monoepoxide also proved to be efficient for this transformation and provided dienes **19** and **20,** which feature both tetra- and trisubstituted double bonds, with excellent selectivity. 2-Phenyl-2-vinyloxirane could also be used, although in that case the corresponding product **21** was obtained in low yield as a 1:1 *E*,*Z*/*Z*,*Z* mixture. It is important to note that this synergistic Cu/Pd catalysis provides access to skipped dienols bearing a tetrasubstituted alkenylboronate (cf. **3**–**6** and **8**–**21**) that cannot be synthesized using any other current methodology.^15^

Cyclic vinyl carbonates have also been used to generate allylic alcohol derivatives via metal-catalyzed reactions with several nucleophiles.^16^ We thus explored this class of compounds in our Cu/Pd-catalyzed allylboration reaction (Scheme 3). Vinyl carbonate **22** proved to be less efficient than 1,3-butadiene epoxide, providing bifunctional diene **3** in almost negligible yield under the optimized conditions. The use of 2 equiv of NaO^*t*^Bu increased the reaction efficiency, although **3** was obtained in only 35% yield. These reaction conditions were also applied to phenyl-substituted carbonate **23**. Surprisingly, no alkenylboronate was obtained in this reaction. When 1 equiv of **23** was used, only protodeboronation product **24** was obtained, while the use of 2 equiv of the vinyl carbonate led to the stereoselective formation of **24** and skipped triene–diol **25** featuring two *Z*-configured allylic alcohols. Comparison of this result with the reaction using 2-phenyl-2-vinyloxirane (cf. formation of **21**) suggests the presence of different allylpalladium intermediates depending on the allylic substrate that is used.^16c^

**Scheme 3:**
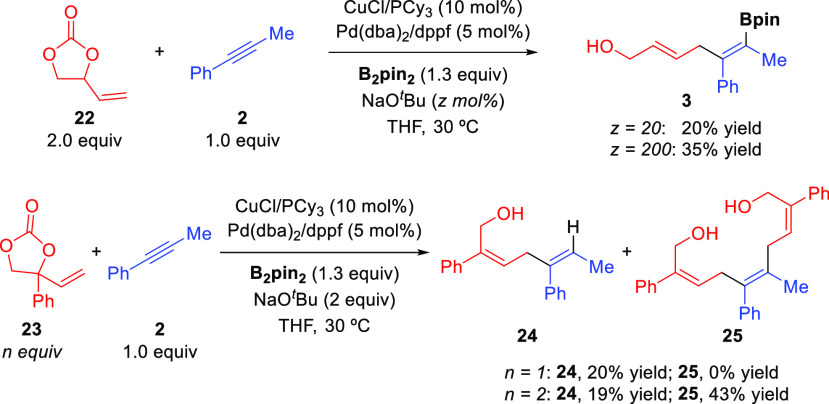
Cu/Pd-Catalyzed Alkyne Allylboration with Cyclic Vinyl Carbonates

On the basis of our experimental observations and our previous investigations,^7^ we propose the following mechanism for the Cu/Pd-catalyzed alkyne allylboration with vinyl epoxides (Scheme 4a). Regio- and stereoselective addition of the LCu–Bpin complex across alkyne **2** would generate β-borylalkenylcopper(I) intermediate **I**. In the second catalytic cycle, zwitterionic η^3^-allylpalladium complex **II** would be formed by oxidative addition of vinyl epoxide **1** to the L′Pd(0) complex. Transmetalation^7b^ between these two organometallic species would give rise to bimetallic intermediate **III**, which would undergo reductive elimination with concomitant regeneration of the Pd(0) catalyst and formation of copper alkoxide **IV**. This intermediate would be reactive enough to undergo σ-bond metathesis with B_2_pin_2_, resulting in the recovery of the active LCu–Bpin complex and the formation of intermediate **V**, which would lead to bifunctional diene **3** by protonation. The reaction between intermediate **IV** and B_2_pin_2_ would explain the viability of performing this reaction with a catalytic amount of NaO^*t*^Bu.

**Scheme 4:**
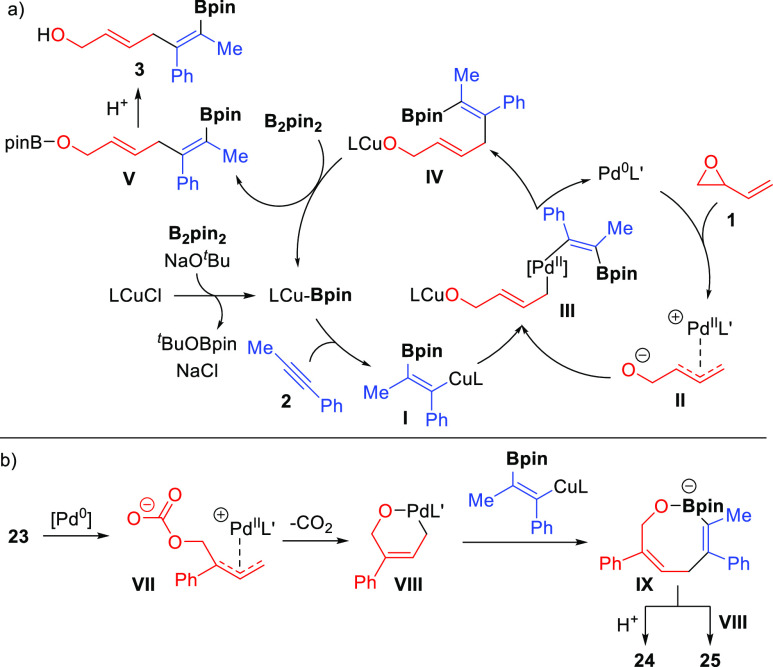
Proposed Mechanism

The differences observed in the reaction with cyclic vinyl carbonate **23** may account for the formation of a different allylpalladium intermediate (Scheme 4b). In this case, extrusion of carbon dioxide from η^3^-allylpalladium open carbonate **VII** may lead to the formation of η^1^-oxapalladacycle **VIII** in which the double bond is electronically stabilized by conjugation with the phenyl ring.^16b,16c^ The *Z* configuration of this palladacycle would define the stereocontrol toward a (*Z*)-allyl alkoxide after reaction with the alkenylcopper species. The *Z*,*Z* configuration could facilitate intramolecular oxygen–boron coordination leading to eight-membered boron intermediate **IX**,^17^ which would undergo protodeboronation^18^ to form **24**. In the presence of excess vinyl carbonate **23**, intermediate **IX** would undergo a Suzuki-type reaction to afford **25**.

An attractive feature of this new allylboration reaction with vinyl epoxides is the combination of an allylic alcohol and an alkenylboronate present in the products, which makes them highly versatile building blocks. Notably, the rhenium-catalyzed allylic [1,3] transposition^19^ of bifunctional dienes **4** and **8** resulted in an efficient synthesis of cyclic boronic acids **26** and **27** (Scheme 5a). These boracycles are important structures since they are valuable synthetic intermediates^20^ and have recently gained increased interest in the drug discovery process in the pharmaceutical industry.^21^ Furthermore, triene **28** could be obtained from **3** in a stereocontrolled manner via a one-pot allylic [1,3] transposition/Suzuki cross-coupling^22^ (Scheme 5b). The presence of the alkenylboronate unit also offers a synthetic handle to easily convert the products into 6-hydroxy-2-aryl ketones by treatment with sodium perborate, as illustrated with the synthesis of compound **29** (Scheme 5c).

**Scheme 5:**
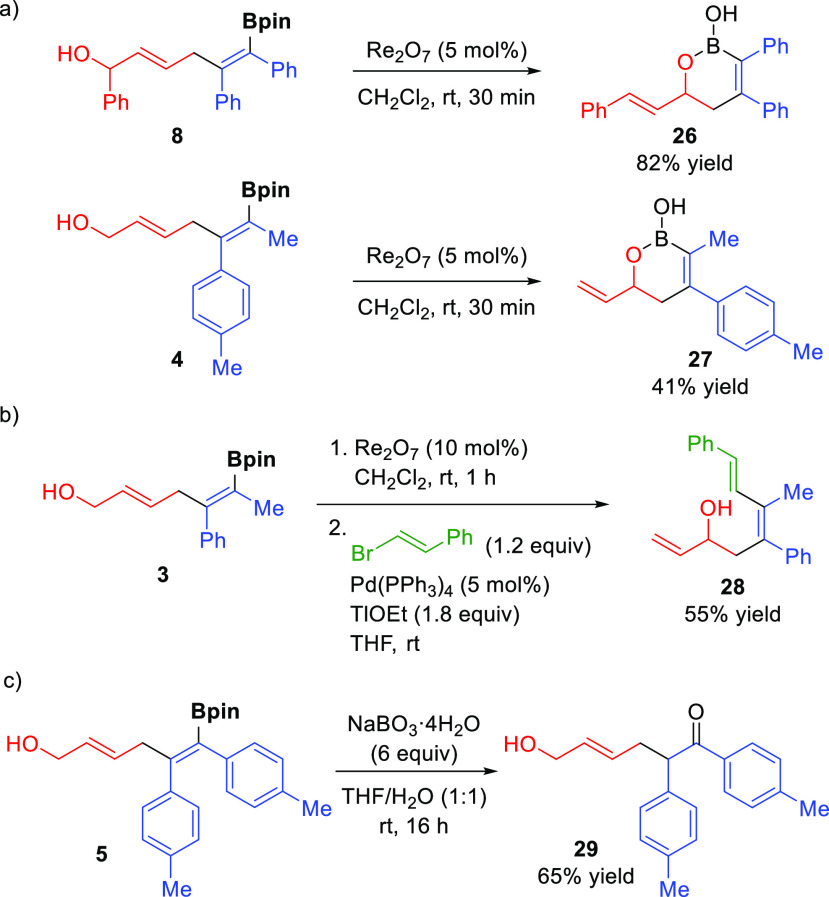
Synthetic Modifications of Bifunctional Dienes

In summary, we have described a synergistic Cu/Pd-catalyzed three-component reaction of alkynes, B_2_pin_2_, and vinyl epoxides. This transformation represents an efficient alkenylation of these allylic compounds and provides bifunctional skipped dienes in good yields with remarkable regio- and stereoselectivity. The presence of two versatile and orthogonal functionalities such as an allylic alcohol and an alkenylboronate makes these products very attractive building blocks for chemical synthesis.

## Notes

The data underlying this study are available in the published article and its online Supporting Information.

## Supporting Information Available

Optimization tables, synthetic procedures, and characterization data (PDF)
