# Supplementary material for: Bifunctional Skipped Dienes through Cu/Pd-Catalyzed Allylboration of Alkynes with B2pin2 and Vinyl Epoxides
Source: Org Lett. 2022 Nov 3;24(44):8244–8. doi: 10.1021/acs.orglett.2c03390 (PMC9664487; doi:10.1021/acs.orglett.2c03390)
Supplement: Supplementary file 1 — ol2c03390_si_001.pdf [file ol2c03390_si_001.pdf]

Supporting Information

Bifunctional skipped dienes through Cu/Pd-catalyzed allylboration of alkynes with B_2pin_2 and vinyl epoxides

Nuria Vázquez-Galiñanes, Isabel Velo-Heleno and Martín Fañanás-Mastral*

Centro Singular de Investigación en Química Biolóxica e Materiais Moleculares (CiQUS), Universidade de Santiago de Compostela, 15782 Santiago de Compostela, Spain.

Correspondence to: martin.fananas@usc.es

Table of contents

1.	General methods	S3
2.	List of starting materials	S4
3.	Optimization tables	S5
	Table S1: Screening of solvents and temperature	
	Table S2: Screening of copper catalysts	
	Table S3: Screening of palladium catalysts	
	Table S4: Evaluation of the slow addition time	
	Table S5: Effect of the base	
4.	General procedures for the Cu/Pd-catalyzed allylboration of alkynes	S8
5.	Compound characterization	S9
6.	Synthetic modifications	S16
7.	Unsuccessful substrates	S19
8.	NMR spectra	S20
9.	Stereochemistry determination	S55
10.	Regiochemistry determination of compound 46 (and 6 / 6’)	S60
11.	References	S63

1. General methods

- All reactions were performed under argon atmosphere using oven dried glassware and using standard Schlenk techniques. Solvents were dried using an MBraun SPS 800 system. All chemicals and copper complexes were purchased from Acros Organics Ltd., Aldrich Chemical Co. Ltd., Alfa Aesar, Apollo, Strem Chemicals Inc., Fluorochem Ltd. or TCI Europe N.V. chemical companies and used without further purification, unless otherwise noted.

- Analytical thin layer chromatography was carried out on silica-coated aluminium plates (silica gel 60 F254 Merck) and components were visualized by UV light, I_2 and $KMnO_4$ staining. Flash column chromatography was performed on silica gel 60 (Merck, 230-400 mesh) without previous deactivation, unless otherwise stated.

- GC-MS analyses were performed in an Agilent instrument GC-6890N equipped with Chemical Ionization (CI) MS-5973 detector.

- High Resolution Mass spectrometry was carried out on a Bruker microTOF spectrometer using APCI.

- ^{1}H- and ^{13}C-NMR experiments were carried out using a Bruker AVIII-500 MHz or a Varian Mercury 300 MHz NMR spectrometers. Chemical shift values are reported in ppm with the solvent resonance as the internal standard ($CHCl_3$: δ 7.26 for ^{1}H, δ 77.16 for ^{13}C). Coupling constants *J* are given in Hertz (Hz). Multiplicities are reported as follows: s = singlet, d = doublet, t=triplet, q=quartet, p=pentet, m=multiplet or as a combination of them.

- Structural assignments were made with additional information from gCOSY, gHSQC, and gHMBC experiments.

- Because of quadrupolar relaxation, in all cases the carbon directly attached to the boron atom was not detected by ^{13}C NMR technique.

- In order to preclude side protoboration reactions, commercial alkynes and B_2pin_2 were dried over Na_2SO_4 prior to being used.

2. List of starting materials

Alkynes **2**, **33** and **35**, vinyl epoxides **1**, **43** and **44**, vinyl cyclic carbonate **22** and B_2(pin)$_2$ were obtained from commercial sources and used without further purification. Alkynes **30**-**32**,[1] **34**-**39**,[1] vinyl epoxides **40**,[2] **41**,[2] **42**[3] and vinyl cyclic carbonate **20**[4] were prepared following described procedures.

- **Alkynes**

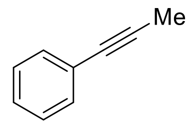

2

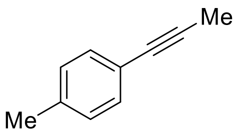

30

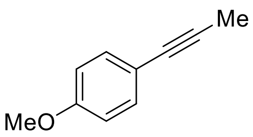

31

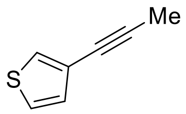

32

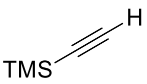

33

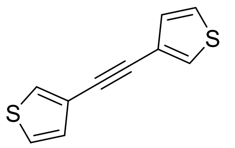

34

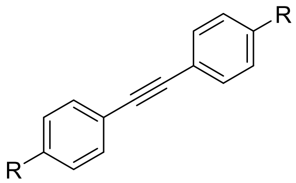

35, R= H **36**, R= Br
37, R= Me **38**, R= CF_3

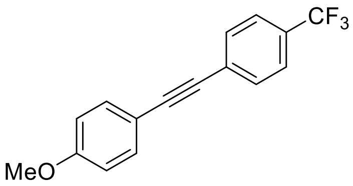

39

- **Vinyl epoxides**

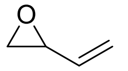

1

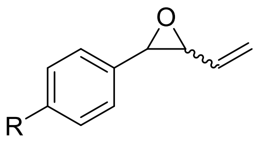

40, R= H (2:1 cis/trans)
41, R= CF_3 (5:1 cis/trans)

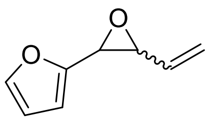

42
5:1 cis/trans

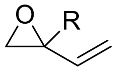

43, R= Me
44, R= Ph

- **Vinyl cyclic carbonates**

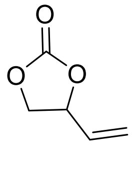

22

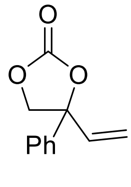

23

3. Optimization studies

3.1 Screening of solvents

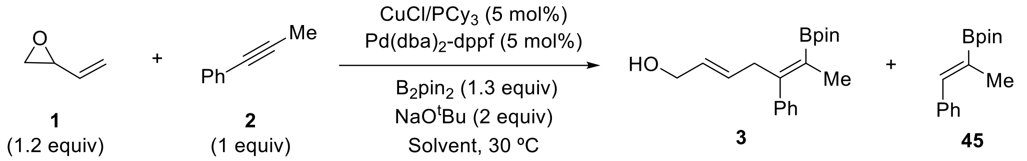

Entry [a]	Solvent	% Conv 2[b]	% yield 3[b]	% yield 45[b]
**1**	THF	92	40	16
**2**	Toluene	87	14	26
**3**	DMF	69	-	25
**4**	DMA	80	-	-

[a] Reactions performed on a 0.3 mmol scale with slow addition of **1** (dissolved in 0.3 ml of THF) over 3 h. [b] Determined by ^{1}H-NMR using 1,3,5-trimethoxybenzene as internal standard

3.2 Screening of copper catalysts

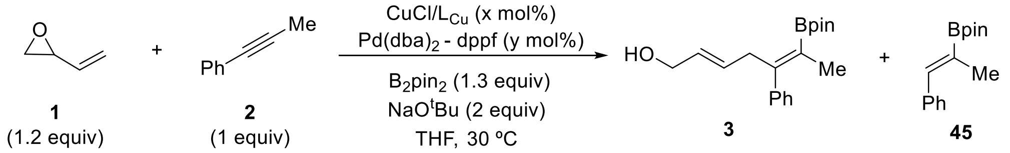

Entry[a]	x:y	L_{Cu}	% Conv 2[b]	% yield 3[b]	% yield 45[b]
**1**	5:5	IPr	40	-	9
**2**	5:5	IMes	63	-	44
**3**	5:5	SIMes	75	-	54
**4**	5:5	PPh_3	63	-	44
**5**	5:5	PCy_3	92	40	16
**6**	10:5	PCy_3	95	47(56)[c]	32
**7**	10:5	BINAP	87	22(20)[c]	56
**8**	10:5	P(*p*-OMe-C_6H_4)$_3$	64	traces	58
**9**	10:2	PCy_3	83	8	11
**10**	5:10	PCy_3	Full	-	51

[a] Reactions performed on a 0.3 mmol scale with slow addition of **1** in 0.3 ml of THF added over 3 h. [b] Determined by ^{1}H-NMR using 1,3,5-trimethoxybenzene as internal standard. [c] Yield of isolated product shown in brackets.

3.3 Screening of palladium catalysts

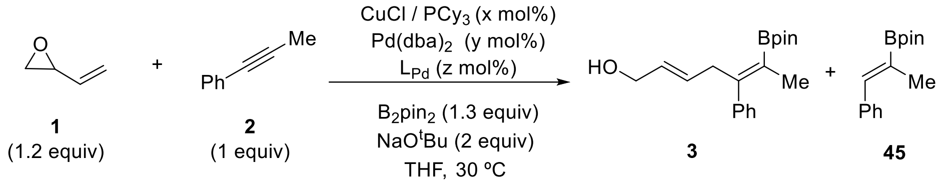

Entry[a]	T (°C)	x:y:z	Pd source	L_{Pd}	% Conv 2[b]	% yield 3[b]	% yield 45[b]
1	50	5:5:5	$Pd(dba)_2$	dppf	nd	10[c]	29
2	50	5:5:5	$Pd(dba)_2$	dppe	95	-	38
3	50	5:5:5	$Pd(dba)_2$	dppm	70	-	31
4	50	5:5:5	$Pd(dba)_2$	PPh_3	82	24	traces
5	50	5:5:5	$Pd_2(dba)_3$	PPh_3	86	16	15
6	50	5:5	$Pd(CH_3CN)Cl_2$		90	5	10
7	30	5:5:5	$Pd(dba)_2$	PPh_3	Full	18	26
8	30	5:5:5	$Pd(dba)_2$	$P(p\text{-}F\text{-}C_6H_4)_3$	92	traces	14
9	30	5:5:5	$Pd(dba)_2$	$P(p\text{-}OMe\text{-}C_6H_4)_3$	90	-	19
10[d]	30	5:5:10	$Pd_2(dba)_3$	PPh_3	79	19	26
11	30	5:5:5	$Pd(dba)_2$	dppf	94	10(13)[e]	63(70)[e]
12	30	10:5:5	$Pd(dba)_2$	dppf	90	19	9
13[f]	30	5:2.5	$[PdCl(C_3H_5)]_2$		64	-	40
14[f]	30	10:5:5	$Pd(dba)_2$	dppf	95	47(56)[e]	32
15[f]	30	10:5:5	$Pd(dba)_2$	dippf	52	traces	-
16[f]	30	10:5:5	$Pd(dba)_2$	L2	85	traces	60
17[f]	30	10:5:5	$Pd(dba)_2$	L3	82	traces	48
18[f]	30	10:5	Pd-dppf G3		74	15	52

[a]Reactions performed on a 0.3 mmol scale. [b]Determined by ^{1}H-NMR using 1,3,5-trimethoxybenzene as internal standard. [c]1:1 mixture of *Z,E*:*Z,Z* isomers. [d]Use of 10 mol% of PPh_3. [e]Yield of isolated product shown in brackets. [f]Slow addition of 0.36 mmol of **1** in 0.3 ml of THF added over 3 h.

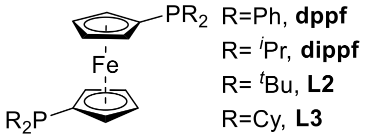

3.4 Evaluation of the slow addition time

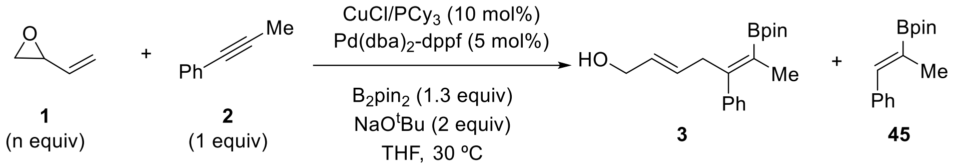

Entry[a]	Slow addition time (h)	Vol. THF for the addition (ml)	Equiv 1	% Conv 2[b]	% yield 3[b]	% yield 45[b]
**1**	-	-	2	66	15	20
**2**	5	0.5	2	95	35	53
**3**	3	0.5	2	91	34	39
**4**	1	0.5	2	90	70(65)[c]	10
**6**	3	0.3	1.2	95	52(56)[c]	32
**7**	1	0.3	1.2	75	32(28)[c]	15
**8**	1	0.3	2	84	41(34)[c]	24

[a]Reactions performed on a 0.3 mmol scale. [b]Determined by ^{1}H-NMR using 1,3,5-trimethoxybenzene as internal standard. [c]Yield of isolated product shown in brackets.

3.5 Effect of the base

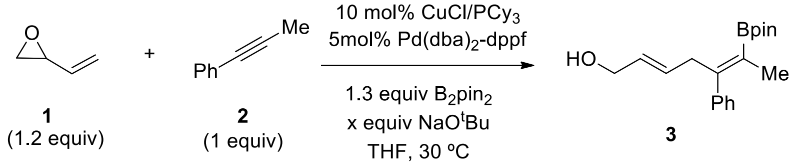

Entry[a]	Base	Equivalents of base	% Conv 2[b]	% yield 3[b]
**1**	NaO^{t}Bu	3	66	15
**2**	NaO^{t}Bu	2	90	70(65)[c]
**3**	NaO^{t}Bu	1.5	90	65
**4**	NaO^{t}Bu	1.2	95	60
**5**	NaO^{t}Bu	0.2	Full	75(70)[c]
**6**	LiO^{t}Bu	0.2	Full	74
**7**	KO^{t}Bu	0.2	Full	56

[a] Reactions performed on a 0.3 mmol scale with slow addition of 0.36 mmol of **1** in 0.3 ml of THF added in 3 h. [b] Determined by ^{1}H-NMR using 1,3,5-trimethoxybenzene as internal standard. [c] Yield of isolated product shown in brackets.

4. General procedures

General procedure for the Cu/Pd allylboration of alkynes with vinyl epoxides (General procedure A)

In a dry vial CuCl (10 mol%, 3 mg, 0.1 mmol,) and Tricyclohexylphosphine (10 mol%, 23.8 mg, 0.1 mmol) were suspended in dry THF (0.3 mL) and stirred during 15 minutes at room temperature. After this time the corresponding alkyne (1 equiv, 0.3 mmol) was added over the previous solution. The final solution was added to the reaction vial containing B_2pin_2 (1.3 equiv, 99.1 mg, 0.39 mmol) and NaO^tBu (20 mol%, 5.8 mg, 0.06 mmol) to afford a red suspension. In a separate vial $Pd(dba)_2$ (5 mol%, 8.62 mg, 0.015 mmol) and dppf (5 mol%, 8.32 mg, 0.015 mmol) were stirred in dry THF (0.3 mL) for 15 minutes at room temperature and added to the reaction vial. Then, a solution of vinyl epoxide (2 equiv, 0.6 mmol) in dry THF (0.5 ml) was added dropwise over 1 hour using a syringe pump. When the addition was completed, the mixture was stirred for additional 14 h at 30 °C in an oil bath. After this time, the reaction was quenched by addition of saturated aqueous solution of NH_4Cl (10 mL) and extracted with EtOAc (2 x 10 mL). The organic layer was dried over Na_2SO_4, filtered and solvent was removed under vacuum. The final product was purified by silica gel column chromatography using the indicated mixture of eluents for each case.

General procedure for the Cu/Pd allylboration of alkynes with vinyl cyclic carbonates (General procedure B)

In a dry vial CuCl (10 mol%, 9.91 mg, 0.1 mmol,) and Tricyclohexylphosphine (10 mol%, 8.41 mg, 0.03 mmol) were suspended in dry THF (1 mL) and stirred during 15 minutes at room temperature. After this time the corresponding alkyne (1 equiv, 116.6 mg, 1 mmol) was added over the previous solution. The final solution was added to the reaction vial containing B_2pin_2 (1.3 equiv, 328.9 mg, 1.3 mmol) and NaO^tBu (2 equiv, 192.2 mg, 2 mmol) to afford a red suspension. In a separate vial $Pd(dba)_2$ (5 mol%, 28.75 mg, 0.05 mmol) and dppf (5 mol%, 27.95 mg, 0.05 mmol) were stirred in dry THF (1 mL) for 15 minutes at room temperature and added to the reaction vial. Then, a solution of vinyl cyclic carbonate (2 equiv, 381.8 mg, 2 mmol) in dry THF (1.6 ml) was added dropwise over 1 hour using a syringe pump. When the addition was completed, the mixture was stirred for additional 14 h at 30 °C in an oil bath. After this time, the reaction was quenched by addition of saturated aqueous solution of NH_4Cl (10 mL) and extracted with EtOAc (2 x 10 mL). The organic layer was dried over Na_2SO_4, filtered and solvent was removed under vacuum. The final product was purified by silica gel column chromatography.

5. Compound characterization

(2*E*,5*Z*)-5-Phenyl-6-(4,4,5,5-tetramethyl-1,3,2-dioxaborolan-2-yl)hepta-2,5-dien-1-ol (3)

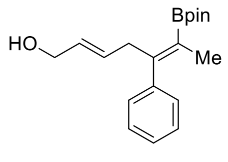

Synthesized from **1**, **2** and B_2pin_2 according to general procedure A. Yellow oil obtained in 70% yield (65.8 mg) after column chromatography (Hexane / EtOAc 4:1).

1 mmol scale: synthesized from **1** (116.1 mg), **2** (140mg) and B_2pin_2 (330.1 mg) according to general procedure A. Yellow oil obtained in 52% yield (165 mg).

^{1}H NMR (500 MHz, $CDCl_3$) δ 7.34 – 7.29 (m, 2H), 7.23 – 7.18 (m, 1H), 7.09 – 7.02 (m, 2H), 5.59 – 5.54 (m, 2H), 3.99 (d, *J* = 4.7 Hz, 2H), 3.43 – 3.38 (d, d, *J* = 4.7 Hz, 2H), 1.58 (s, 3H), 1.32 (s, 12H). **^{13}C NMR** (126 MHz, $CDCl_3$) δ 152.4 (C), 142.9 (C), 131.4 (CH) 129.80 (CH), 128.1 (2 x CH), 127.9 (2 x CH), 126.40 (CH), 83.2 (2 x C), 63.8 (CH_2), 40.8 (CH_2), 24.9 (4 x CH_3), 18.3 (CH_3) **^{11}B NMR** (160 MHz, $CDCl_3$) δ 31.45. **HRMS** (APCI) m/z: [$M-H_2O+H^+$]: Calc. for $C_{19}H_{26}BO_2$ 297.2020; found 297.2022.

(2*E*,5*Z*)-6-(4,4,5,5-Tetramethyl-1,3,2-dioxaborolan-2-yl)-5-(p-tolyl)hepta-2,5-dien-1-ol (4)

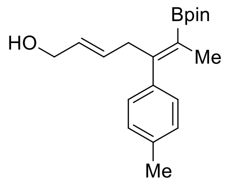

Synthesized from **1**, **30** and B_2pin_2 according to general procedure A. Yellow oil obtained in 57% yield (65.8 mg) after column chromatography (Hexane / EtOAc 4:1).

^{1}H NMR (500 MHz, $CDCl_3$) δ 7.15 – 7.13 (m, 2H), 7.00 – 6.98 (m, 2H), 5.61 – 5.58 (m, 2H), 4.03 – 4.00 (m, 2H), 3.43 – 3.40 (m, 2H), 2.36 (s, 3H), 1.61 (s, 3H), 1.34 (s, 12H). **^{13}C NMR** (126 MHz, $CDCl_3$) δ 152.3 (C), 139.9 (C), 135.9 (C), 131.6 (CH), 129.6 (CH), 128.6 (2 x CH), 128.0 (2 x CH), 83.2 (2 x C), 63.8 (CH_2), 40.8 (CH_2), 24.9 (4 x CH_3), 21.2 (CH_3), 18.4 (CH_3). **^{11}B NMR** (160 MHz, $CDCl_3$) δ 31.42. **HRMS** (APCI) m/z: [$M-H_2O+H^+$]: Calc. for $C_{20}H_{28}BO_2$ 311.2177; found 311.2181.

(2*E*,5*Z*)-6-(4,4,5,5-Tetramethyl-1,3,2-dioxaborolan-2-yl)-5,6-di-p-tolylhexa-2,5-dien-1-ol (5)

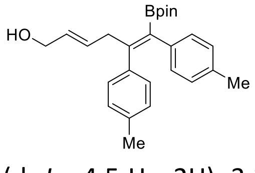

Synthesized from **1**, **37** and B_2pin_2 according to general procedure A. Yellow oil obtained in 40% yield (56.0 mg) after column chromatography (Hexane / EtOAc 4:1).

^{1}H NMR (500 MHz, $CDCl_3$) δ 6.88 – 6.71 (m, 8H), 5.60 – 5.56 (m, 2H), 3.93 (d, *J* = 4.5 Hz, 2H), 3.38 (d, *J* = 4.4 Hz, 2H), 2.16 (s, 3H), 2.13 (s, 3H), 1.24 (s, 12H).**^{13}C NMR** (75 MHz, $CDCl_3$) δ 149.9 (C), 138.9 (C), 138.4 (C) 135.9 (C), 134.7 (C), 130.9 (CH), 130.3 (CH), 129.5 (2 x CH), 129.1 (2 x CH), 128.4 (2 x CH), 128.3 (2 x CH), 83.68 (2 x C), 63.7 (CH_2), 41.4 (CH_2), 29.70, 24.8 (4 x CH_3), 21.1 (CH_3), 21.1 (CH_3). **^{11}B NMR** (160 MHz, $CDCl_3$) δ 30.80. **HRMS** (APCI) m/z: [$M-H_2O+H^+$]: Calc. for $C_{26}H_{32}BO_2$ 387.2490; found 387.2492.

(2*E*,5*Z*)-5-(4-Methoxyphenyl)-6-(4,4,5,5-tetramethyl-1,3,2-dioxaborolan-2-yl)-6-(4-(trifluoromethyl)phenyl)hexa-2,5-dien-1-ol (6) and **(2*E*,5*Z*)-6-(4-Methoxyphenyl)-6-(4,4,5,5-tetramethyl-1,3,2-dioxaborolan-2-yl)-5-(4-(trifluoromethyl)phenyl)hexa-2,5-dien-1-ol (6')**

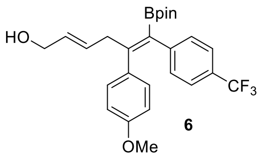

6

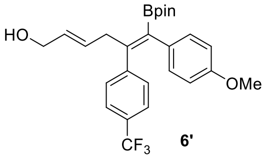

6'

Synthesized from **1**, **39** and B_2pin_2 according to general procedure A. Yellow oil obtained in 43% yield (61.0 mg) after column chromatography (Hexane / EtOAc 4:1) as a 2:1 mixture of regioisomers **6** and **6'**.

^{1}H NMR (300 MHz, $CDCl_3$) δ 7.38 (d, *J* = 8.0 Hz, 2H, **6'**), 7.33 (d, *J* = 8.1 Hz, 2H, **6**), 7.11 (d, *J* = 8.3 Hz, 2H, **6'**), 7.05 (d, *J* = 8.0 Hz, 2H, **6**), 6.90 – 6.82 (m, 2H, **6** + **6'**), 6.70 – 6.57 (m, 2H, **6** + **6'**), 5.73 – 5.55 (m, 2H, **6** + **6'**), 4.17 – 3.96 (m, 2H, **6** + **6'**), 3.74 (s, 3H, **6**), 3.72 (s, 3H, **6'**), 3.58 – 3.46 (m, 2H, **6** + **6'**), 1.35 (s, 12H, **6'**), 1.34 (s, 12H, **6**). **^{13}C NMR** (75 MHz, $CDCl_3$) δ 158.3 (C, **6**), 157.7 (C, **6'**), 153.0 (C, **6** + **6'**), 148.2 (C, **6'**), 145.9 (C, **6**), 135.2 (**6'**), 133.6 (C, **6**), 132.9 (C, **6'**), 132.2 (CH, **6'**), 130.9 (CH, **6'**), 130.6 (CH, **6**), 130.3 (CH, **6**), 129.9 (2 x CH, **6**), 129.8 (2 x CH, **6'**), 129.5 (2 x CH, **6**), 124.66 (q, *J* = 3.8 Hz, 2 x CH, **6'**), 124.4 (q, *J* = 3.8 Hz, 2 x CH, **6)**, 113.2 (2 x CH, **6**), 83.9 (2 x C, **6'**), 83.8 (2 x C, **6**), 63.6 (CH_2, **6**), 63.4 (CH_2, **6'**), 55.05 (CH_3, **6**), 55.0 (CH_3, **6'**), 41.1 (CH_2, **6**), 40.9 (CH_2, **6'**), 24.7 (4 x CH_3, **6** + **6'**). **^{19}F NMR** (282 MHz, $CDCl_3$) δ -62.26 (**6**), -62.44 (**6'**). **^{11}B NMR** (160 MHz, $CDCl_3$) δ 31.29. **HRMS** (APCI) m/z: [M-H_2O+H^+]: Cal. for $C_{26}H_{29}BF_3O_3$ 457.2156; found 457.2168.

(2*E*,5*E*)-6-(4,4,5,5-Tetramethyl-1,3,2-dioxaborolan-2-yl)-5-(trimethylsilyl)hexa-2,5-dien-1-ol (7)

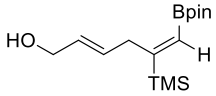

Synthesized from **1**, **33** and B_2pin_2 according to general procedure A. Yellow oil obtained in 56% yield (49.5 mg) after column chromatography (Hexane / EtOAc 4:1).

^{1}H NMR (500 MHz, $CDCl_3$) δ 5.93 (s, 1H), 5.67 – 5.64 (m, 2H), 4.08 (d, *J* = 3.9 Hz, 2H), 3.31 – 3.28 (m, 2H), 1.28 (s, 12H), 0.08 (s, 9H). **^{13}C NMR** (126 MHz, $CDCl_3$) δ 168.2 (C), 132.7 (CH), 129.5 (CH), 83.0 (2 x C), 63.9 (CH_2), 37.5 (CH_2), 24.9 (4 X CH_3), 1.2 (3 x CH_3). **^{11}B NMR** (160 MHz, $CDCl_3$) δ 28.80. **HRMS** (APCI) m/z: [M-H_2O+H^+]: Calc. for $C_{15}H_{28}BO_2Si$ 279.1946; found 279.1941.

(2*E*,5*Z*)-1,5-Diphenyl-6-(4,4,5,5-tetramethyl-1,3,2-dioxaborolan-2-yl)hepta-2,5-dien-1-ol (8)

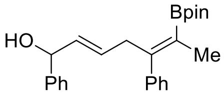

Synthesized from **2**, **40** and B_2pin_2 according to general procedure A at 50 °C. Yellow oil obtained in 71% yield (83 mg) after column chromatography (Hexane / EtOAc 4:1).

^{1}H NMR (500 MHz, $CDCl_3$) δ 7.26 – 7.18 (m, 5H), 7.16 – 7.11 (m, 3H), 6.99 – 6.93 (m, 2H), 5.58 (dtd, *J* = 14.9, 6.9, 1.0 Hz, 1H), 5.49 – 5.41 (m, 1H), 4.99 (d, *J* = 7.0 Hz, 1H), 3.43 – 3.28 (m, 2H), 1.51 (s, 3H), 1.23 (s, 12H). **^{13}C NMR** (126 MHz, $CDCl_3$) δ 152.4 (C), 143.1 (C), 142.9 (C), 133.3 (CH), 128.3 (2 x CH), 128.1 (2 x CH), 127.9 (2 x CH), 127.3 (CH), 126.3 (CH), 126.2 (2 x CH), 83.2 (2 x C), 74.9 (CH), 40.7 (CH_2), 24.9 (4 x CH_3), 18.4 (CH_3). **^{11}B NMR** (160 MHz, $CDCl_3$) δ 32.49. **HRMS** (APCI) m/z: [M-H_2O+H^+]: Calc. for $C_{25}H_{30}BO_2$ 373.2333; found 373.2348.

(2*E*,5*Z*)-1,5,6-Triphenyl-6-(4,4,5,5-tetramethyl-1,3,2-dioxaborolan-2-yl)hexa-2,5-dien-1-ol (9)

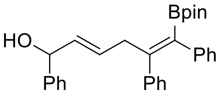

Synthesized from **35**, **40** and B_2pin_2 according to general procedure A at 50 °C. Yellow oil obtained in 46% yield (62.4 mg) after column chromatography (Hexane / EtOAc 4:1).

^{1}H NMR (300 MHz, $CDCl_3$) δ 7.32 – 7.19 (m, 5H), 7.10 – 6.95 (m, 10H), 5.73 (ddd, *J* = 34.4, 15.3, 7.7 Hz, 2H), 5.11 (d, *J* = 6.5 Hz, 1H), 3.55 (dd, *J* = 6.02, 2.3, 2H), 1.35 (s, 12H). **^{13}C NMR** (75 MHz, $CDCl_3$) δ 151.0 (C), 141.9(C), 141.4 (C), 134.0 (C), 129.9 (CH), 129.7 (2 x CH), 129.3 (2 x CH), 128.3 (2 x CH), 127.7 (2 x CH), 127.5 (2 x CH), 127.4 (CH), 126.4 (CH), 126.3 (2 x CH), 125.5 (CH), 83.8 (2 x C), 74.7 (CH), 41.3 (CH_2), 24.8 (4 x CH_3). **^{11}B NMR** (160 MHz, $CDCl_3$) δ 30.5. **HRMS** (APCI) m/z: [M-H_2O+H^+]: Calc. for $C_{30}H_{32}BO_2$ 435.2490; found 435.2496.

(2*E*,5*Z*)-5,6-Bis(4-bromophenyl)-1-phenyl-6-(4,4,5,5-tetramethyl-1,3,2-dioxaborolan-2-yl)hexa-2,5-dien-1-ol (10)

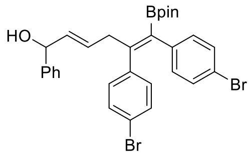

Synthesized from **36**, **40** and B_2pin_2 according to general procedure A at 50 °C. Yellow oil obtained in 26% yield (47.4 mg) after column chromatography (Hexane / EtOAc 4:1).

^{1}H NMR (500 MHz, $CDCl_3$) δ 7.26 – 7.05 (m, 9H), 6.73 – 6.68 (m, 3H), 5.67 – 5.47 (m, 2H), 5.01 (dd, *J* = 6.7, 3.2 Hz, 1H), 3.41 (qd, *J* = 14.5, 6.6 Hz, 2H), 1.21 (s, 11H). **^{13}C NMR** (126 MHz, $CDCl_3$) δ 149.9 (C), 141.8 (C), 139.4 (C), 139.1 (C), 133.4 (CH), 130.3 (2 x CH), 130.0 (2 x CH), 129.8 (2 x CH), 129.8 (2 x CH), 128.0 (CH), 127.4 (2 x CH), 126.5 (CH), 125.1 (2 x CH), 119.7 (C), 118.7 (C), 82.9 (2 x C), 73.7 (CH), 39.9 (CH_2), 23.7 (4 x CH_3).**^{11}B NMR** (160 MHz, $CDCl_3$) δ 30.97. **HRMS** (APCI) m/z: [M-H_2O+H^+]: Calc. for $C_{30}H_{30}BBr_2O_2$ 591.0700; found 591.0693.

(2*E*,5*Z*)-1-Phenyl-6-(4,4,5,5-tetramethyl-1,3,2-dioxaborolan-2-yl)-5,6-di-p-tolylhexa-2,5-dien-1-ol (11)

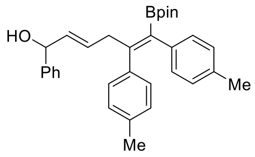

Synthesized from **40**, **37** and B_2pin_2 according to general procedure A at 50 °C. Yellow oil obtained in 59% yield (73.9 mg) after column chromatography (Hexane / EtOAc 4:1).

^{1}H NMR (300 MHz, $CDCl_3$) δ 7.34 – 7.15 (m, 4H), 6.96 – 6.81 (m, 9H), 5.85 – 5.57 (m, 2H), 5.09 (d, *J* = 6.7 Hz, 1H), 3.50 (d, *J* = 5.7 Hz, 2H), 2.26 (s, 3H), 2.23 (s, 3H), 1.32 (s, 12H). **^{13}C NMR** (75 MHz, $CDCl_3$) δ 150.1 (C), 143.1 (C), 138.9 (C), 138.5 (C), 135.8 (C), 134.7 (C), 133.8 (CH), 130.2 (CH), 129.5 (2 x CH), 129.2 (2 x CH), 128.4 (2 x CH), 128.3 (2 x CH), 128.2 (2 x CH), 127.3 (CH), 126.3 (2 x CH), 83.7 (2 x C), 74.8 (CH), 41.4 (CH_2), 24.8 (4 x CH_3), 21.13 (2 x CH). **^{11}B NMR** (160 MHz, $CDCl_3$) δ 30.35. **HRMS** (APCI) m/z: [M-H_2O+H^+]: Calc. for $C_{32}H_{36}BO_2$ 463.2803; found 463.2803.

(2*E*,5*Z*)-1-Phenyl-6-(4,4,5,5-tetramethyl-1,3,2-dioxaborolan-2-yl)-5,6-bis(4-(trifluoromethyl)phenyl)hexa-2,5-dien-1-ol (12)

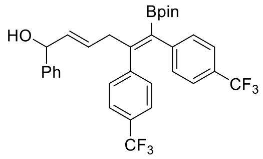

Synthesized from **38**, **40** and B_2pin_2 according to general procedure A at 50 °C. Yellow oil obtained in 46% yield (81.2 mg) after column chromatography (Hexane / EtOAc 4:1).

^{1}H NMR (300 MHz, $CDCl_3$) δ 7.61 – 7.21 (m, 5H), 7.17 (d, *J* = 9.5 Hz, 1H), 7.03 (d, *J* = 7.2 Hz, 4H), 5.82 – 5.55 (m, 2H), 5.11 (d, *J* = 6.1 Hz, 1H), 3.69 – 3.48 (m, 2H), 1.33 (s, 12H).**^{13}C NMR** (75 MHz, $CDCl_3$) δ 151.9 (C), 145.2 (C), 144.9 (C), 142.9 (C), 134.8 (CH), 129.8 (2 x CH), 129.4 (2 x CH), 128.5 (CH), 128.4 (2 x CH), 127.6 (CH), 126.1 (2 x CH), 124.7 (q, *J* = 4.0 Hz, 2 x CH), 124.61 (q, *J* = 4.0 Hz, 2 x CH), 84.1 (2 x C), 74.7 (CH_2), 40.9 (CH_2), 24.7 (4 x CH_3). **^{19}F NMR** (282 MHz, $CDCl_3$) δ -62.39, -62.51. **^{11}B NMR** (160 MHz, $CDCl_3$) δ 30.31. **HRMS** (APCI) m/z: [M-H_2O+H^+]: Calc. for $C_{32}H_{30}BF_6O_2$ 571.2238; found 571.2245.

(2*E*,5*Z*)-5-Phenyl-6-(4,4,5,5-tetramethyl-1,3,2-dioxaborolan-2-yl)-1-(4 (trifluoromethyl)phenyl)hepta-2,5-dien-1-ol (13)

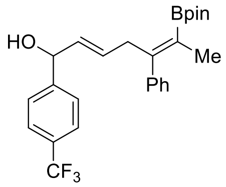

Synthesized from **2**, **41** and B_2pin_2 according to general procedure A at 50 °C. Yellow oil obtained in 83% yield (114 mg) after column chromatography (Hexane / EtOAc 4:1).

^{1}H NMR (300 MHz, $CDCl_3$) δ 7.58 – 7.52 (m, 2H), 7.35 – 7.23 (m, 5H), 7.08 – 7.02 (m, 2H), 5.74 – 5.64 (m, 1H), 5.47 (ddt, *J* = 15.2, 7.2, 1.3 Hz, 1H), 5.11 (d, *J* = 7.57 Hz, 1H), 3.56 – 3.35 (m, 2H), 1.61 (s, 3H), 1.31 (s, 12H). **^{13}C NMR** (75 MHz, $CDCl_3$) δ 152.0 (C), 146.9 (C), 142.7 (C), 132.7 (CH), 131.8 (CH), 128.1 (2 x CH), 128.0 (2 x CH), 126.5 (CH) 126.4 (2 X CH), 126.4 (CH), 125.1 (q, *J* = 3.4 Hz, 2 x CH), 83.3 (2 x C), 74.4 (CH), 40.6 (CH_2), 24.8 (4 x CH_3), 18.4 (CH_3). **^{19}F NMR** (282 MHz, $CDCl_3$) δ -62.90. **^{11}B NMR** (160 MHz, $CDCl_3$) δ 30.93. **HRMS** (APCI) m/z: [M-H_2O+H^+]: Calc. $C_{26}H_{29}BF_3O_2$ 441.2211; found 441.2213

(2*E*,5*Z*)-5-(4-Methoxyphenyl)-6-(4,4,5,5-tetramethyl-1,3,2-dioxaborolan-2-yl)-1-(4-(trifluoromethyl)phenyl)hepta-2,5-dien-1-ol (14)

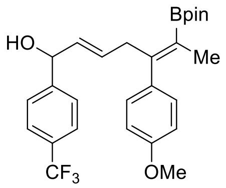

Synthesized from **31**, **41** and B_2pin_2 according to general procedure A at 50 °C. Yellow oil obtained in 67% yield (98 mg) after column chromatography (Hexane / EtOAc 4:1).

^{1}H NMR (500 MHz, $CDCl_3$) δ 7.55 – 7.52 (m, 2H), 7.32 – 7.29 (m, 2H), 6.99 – 6.96 (m, 2H), 6.84 – 6.82 (m, 2H), 5.67 (dtd, *J* = 15.0, 6.9, 0.9 Hz, 1H), 5.46 (ddt, *J* = 15.2, 7.3, 1.4 Hz, 1H), 5.11 (d, *J* = 7.3 Hz, 1H), 3.80 (s, 3H), 3.49 (dd, *J* = 14.1, 6.6 Hz, 1H), 3.40 – 3.34 (m, 1H), 1.63 (s, 3H), 1.30 (s, 12H). **^{13}C NMR** (126 MHz**,** $CDCl_3$**)** δ 158.1 (C), 151.6 (C), 146.9 (C), 134.9 (C), 132.6 (CH, 131.9 (CH), 129.3 (2 x CH), 126.4 (2 x CH), 125.1 (q, *J* = 3.8 Hz, 2 x CH), 113.3 (2 x CH), 83.2 (2 x C), 74.4 (CH), 55.1 (CH_3), 40.8 (CH_2), 24.8 (4 x CH_3), 18.5 (CH_3). **^{19}F NMR** (282 MHz, $CDCl_3$) δ -62.54. **^{11}B NMR** (160 MHz, $CDCl_3$) δ 30.84. **HRMS** (APCI) m/z: [M-H_2O+H^+]: Calc. for $C_{27}H_{31}F_3O_3$ 471.2313; found 471.2315.

(2*E*,5*Z*)-6-(4,4,5,5-Tetramethyl-1,3,2-dioxaborolan-2-yl)-5,6-di-p-tolyl-1-(4-(trifluoromethyl)phenyl)hexa-2,5-dien-1-ol (15)

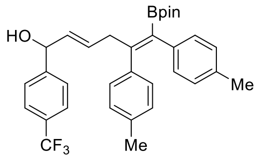

Synthesized from **37**, **41** and B_2pin_2 according to general procedure A at 50 °C. Yellow oil obtained in 63% yield (104 mg) after column chromatography (Hexane / EtOAc 4:1).

^{1}H NMR (300 MHz, $CDCl_3$) δ 7.49 (d, *J* = 8.0 Hz, 2H), 7.27 (d, *J* = 7.8 Hz, 2H), 6.91 – 6.86 (m, 8H), 5.76 (dt, *J* = 13.9, 6.6 Hz, 1H), 5.55 (dd, *J* = 15.3, 7.2 Hz, 1H), 5.10 (d, *J* = 7.3 Hz, 1H), 3.59 – 3.45 (m, 2H), 2.27 (s, 3H), 2.25 (s, 3H), 1.34 (s, 12H). **^{13}C NMR** (75 MHz, $CDCl_3$) δ 149.7 (C), 146.9 (C), 138.8 (C), 138.3 (C), 135.9 (C), 134.8 (C), 133.3 (CH), 129.5 (2 x CH), 129.2 (2 x CH), 128.4 (2 x CH), 128.4 (2 x CH), 126.5 (2 x CH), 125.1 (q, *J* = 3.8 Hz, 2 x CH), 83.7 (2 x C), 74.3 (CH), 41.3 (CH_2), 24.7 (4 x CH_3), 21.1 (2 x CH_3).**^{19}F NMR** (282 MHz, $CDCl_3$) δ -62.45. **^{11}B NMR** (160 MHz, $CDCl_3$) δ 30.91. **HRMS** (APCI) m/z: [$M-H_2O+H^+$]: Cal. for $C_{33}H_{35}BF_3O_2$ 531.2677; found 531.2687.

(2*E*,5*Z*)-6-(4,4,5,5-Tetramethyl-1,3,2-dioxaborolan-2-yl)-5-(thiophen-3-yl)-1-(4-(trifluoromethyl)phenyl)hepta-2,5-dien-1-ol (16)

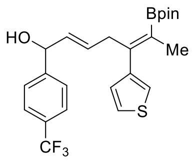

Synthesized from **32**, **41** and B_2pin_2 according to general procedure A at 50 °C. Yellow oil obtained in 53% yield (61.6 mg) after column chromatography (Hexane / EtOAc 4:1).

^{1}H NMR (500 MHz, $CDCl_3$) δ 7.49 (d, *J* = 8.3 Hz, 2H), 7.28 (d, *J* = 8.3 Hz, 2H), 7.22 – 7.16 (m, 1H), 6.92 (d, *J* = 3.0 Hz, 1H), 6.85 (d, *J* = 5.0 Hz, 1H), 5.68 – 5.59 (m, 1H), 5.46 (dd, *J* = 15.3, 7.3 Hz, 1H), 5.07 (d, *J* = 7.2 Hz, 1H), 3.46 – 3.27 (m, 2H), 1.67 (s, 3H), 1.23 (s, 12H). **^{13}C NMR** (75 MHz, $CDCl_3$) δ 147.0 (C), 146.5 (C), 142.7 (C), 132.6 (CH), 132.0 (CH), 128.2 (CH), 126.4 (2 x CH), 125.2 (q, *J* = 3.8 Hz, 2 x CH), 124.5 (CH), 122.1 (CH), 83.3 (2 x C), 74.37, 40.4 (CH_2), 24.8 (4 x CH_3), 18.6 (CH_3). **^{11}B NMR** (160 MHz, $CDCl_3$) δ 31.19.**^{19}F NMR** (282 MHz, $CDCl_3$) δ -62.46. **HRMS** (APCI) m/z: [$M-H_2O+H^+$]: Cal. for $C_{24}H_{27}BF_3O_2S$ 447.1771 found 447.1780.

(2*E*,5*Z*)-6-(4,4,5,5-Tetramethyl-1,3,2-dioxaborolan-2-yl)-5,6-di(thiophen-3-yl)-1-(4-(trifluoromethyl)phenyl)hexa-2,5-dien-1-ol (17)

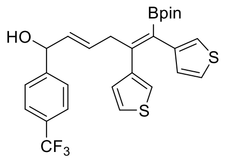

Synthesized from **34**, **41** and B_2pin_2 according to general procedure A at 50 °C. Yellow oil obtained in 45% yield (72.7 mg) after column chromatography (Hexane / EtOAc 4:1).

^{1}H NMR (500 MHz, $CDCl_3$) δ 7.48 (d, *J* = 8.1 Hz, 2H), 7.27 (d, *J* = 8.1 Hz, 2H), 7.01 (dd, *J* = 5.0, 3.1 Hz, 1H), 6.98 (dd, *J* = 4.9, 3.1 Hz, 1H), 6.85 (dd, *J* = 3.0, 1.2 Hz, 1H), 6.82 (dd, *J* = 2.9, 1.2 Hz, 1H), 6.58 (d, *J* = 5.0, 1H), 6.55 (d, *J* = 5.0, 1H), 5.73 (dt, *J* = 15.0, 6.6 Hz, 1H), 5.56 (dd, *J* = 15.4, 7.2 Hz, 1H), 5.09 (d, *J* = 7.2 Hz, 1H), 3.41 (qd, *J* = 14.7, 6.6 Hz, 2H), 1.24 (s, 12H). **^{13}C NMR** (75 MHz, $cdcl_3$) δ 146.9 (C), 144.8 (C), 142.4 (C), 141.3 (C), 133.3 (CH), 131.1 (CH), 129.0 (CH), 128.6 (CH), 126.4 (CH), 125.2 (q, *J* = 3.82 Hz, 2 x CH), 124.3 (CH), 123.8 (CH), 123.2 (CH), 122.4 (CH), 83.8 (CH), 74.2 (CH), 40.9 (CH_2), 24.7 (4 x CH_3). **^{19}F NMR** (282 MHz, $CDCl_3$) δ -62.46.

^{11}B NMR (160 MHz, $CDCl_3$) δ 30.62. **HRMS** (APCI) m/z: [M-H_2O+H^+]: Cal. for $C_{27}H_{27}BF_3O_2S_2$ 515.1492 found 515.1512.

(2E,5Z)-1-(furan-2-yl)-5-phenyl-6-(4,4,5,5-tetramethyl-1,3,2-dioxaborolan-2-yl)hepta-2,5-dien-1-ol (18)

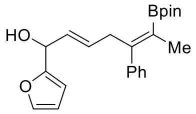

Synthesized from **2**, **42** and B_2pin_2 according to general procedure A at 50 °C. Yellow oil obtained in 39% yield (44.3 mg) after column chromatography (Hexane / EtOAc 4:1).

^{1}H NMR (300 MHz, $CDCl_3$) δ 7.41 – 7.20 (m, 4H), 7.06 (d, *J* = 7.6 Hz, 2H), 6.27 (dd, *J* = 3.2, 1.7 Hz, 1H), 6.01 (d, *J* = 3.3 Hz, 1H), 5.67 (qd, *J* = 15.5, 7.6 Hz, 2H), 5.08 (d, *J* = 6.4 Hz, 1H), 3.46 (d, *J* = 5.6 Hz, 2H), 1.59 (s, 3H), 1.31 (s, 12H). **^{13}C NMR** (75 MHz, $CDCl_3$) δ 155.6 (C), 152.2 (C), 142.9 (C), 142.1 (CH), 131.9 (CH), 129.7 (CH), 128.1 (2 x CH), 127.9 (2 x CH), 126.4 (CH), 110.1 (CH), 106.3 (CH), 83.2 (2 x C), 68.5 (CH), 40.7 (CH_2), 24.9 (4 x CH_3), 18.4 (CH_3). **^{11}B NMR** (160 MHz, $CDCl_3$) δ 31.32. **HRMS** (APCI) m/z: [M-H_2O+H^+]: Cal. for $C_{23}H_{28}BO_3$ 363.2126 found 363.2142.

(2*E*,5*Z*)-2-Methyl-5-phenyl-6-(4,4,5,5-tetramethyl-1,3,2-dioxaborolan-2-yl)hepta-2,5-dien-1-ol (19)

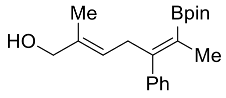

Synthesized from **2**, **43** and B_2pin_2 according to general procedure A. Yellow oil obtained in 36% yield (35.5 mg) after column chromatography (Hexane / EtOAc 4:1).

^{1}H NMR (500 MHz, $CDCl_3$) δ 7.34 – 7.31 (m, 2H), 7.25 – 7.22 (m, 1H), 7.10 – 7.06 (m, 2H), 5.31 (t, *J* = 6.3, 1H), 3.91 (s, 2H), 3.45 (d, *J* = 7.4 Hz, 2H), 1.59 (s, 3H), 1.51 (s, 3H), 1.35 (s, 12H). **^{13}C NMR** (126 MHz, $CDCl_3$) δ 153.2 (C), 143.1 (C), 135.0 (C), 128.0 (2 x CH), 127.9 (2 x CH), 126.3 (CH), 124.6 (CH), 83.2 (2 x C), 69.1 (CH_2), 36.4 (CH_2), 24.9 (4 x CH_3), 18.3 (CH_3), 13.7 (CH_3). **^{11}B NMR** (160 MHz, $CDCl_3$) δ 31.42. **HRMS** (APCI) m/z: [M-H_2O+H^+]: Calc. for $C_{20}H_{28}BO_2$ 311.2153; found 311.2159.

(2*E*,5*Z*)-2-Methyl-6-(4,4,5,5-tetramethyl-1,3,2-dioxaborolan-2-yl)-5,6-di-p-tolylhexa-2,5-dien-1-ol (20)

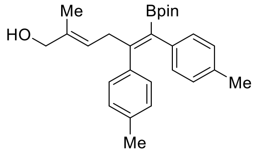

Synthesized from **37**, **43** and B_2pin_2 according to general procedure A. Yellow oil obtained in 48% yield (60.2 mg) after column chromatography (Hexane / EtOAc 4:1).

^{1}H NMR (300 MHz, $CDCl_3$) δ 6.96 – 6.79 (m, 8H), 5.39 (t, *J* = 6.3, 1H), 3.92 (s, 2H), 3.49 (d, *J* = 6.9 Hz, 2H), 2.25 (s, 3H), 2.23 (s, 3H) 1.61 (s, 3H), 1.34 (s, 12H). **^{13}C NMR** (75 MHz, $CDCl_3$) δ 150.9 (C), 139.1 (C), 138.6 (C), 135.7 (C), 135.4 (C), 134.6 (C), 129.5 (2 x CH), 129.0 (2 x CH), 128.3 (2 x CH), 128.2 (2 x CH), 124.5 (CH), 83.6 (2 x C), 69.0 (CH_2), 37.2 (CH_2), 24.8 (4 x CH_3), 21.1 (CH_3), 13.9 (CH_3).**HRMS** (APCI) m/z: [M-H_2O+H^+]: Calc. for $C_{27}H_{34}BO_2$ 401.2646; found 401.2659.

(2*E*,5*Z*)-2,5-Diphenyl-6-(4,4,5,5-tetramethyl-1,3,2-dioxaborolan-2-yl)hepta-2,5-dien-1-ol (21) and **(2*Z*,5*Z*)-2,5-diphenyl-6-(4,4,5,5-tetramethyl-1,3,2-dioxaborolan-2-yl)hepta-2,5-dien-1-ol (21’)**

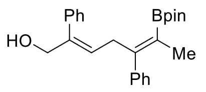

21

+

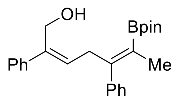

21'

Synthesized from **2**, **44** and B_2pin_2 according to general procedure A. Yellow oil obtained in 28% yield (32.8 mg) after column chromatography (Hexane / EtOAc 4:1) as a 1:1 mixture of stereoisomers **21** and **21’**.

^{1}H NMR (500 MHz, $CDCl_3$) δ 7.38 – 7.19 (m, *J* = 8.0 Hz, 12H, **21** + **21’**), 7.13 (d, *J* = 7.0 Hz, 2H), 7.00 (d, *J* = 8.3 Hz, 2H), 6.89 (d, *J* = 7.7 Hz, 2H), 5.79 (t, *J* = 7.8 Hz, 1H, **21’**), 5.62 (s, 1H, **21**), 4.36 (d, *J* = 5.1 Hz, 2H, **21’**), 4.22 (s, 2H, **21**), 3.68 (d, *J* = 7.8 Hz, 2H, **21’**), 3.41 (d, *J* = 7.1 Hz, 2H, **21**), 1.61 (s, 3H, **21’**), 1.58 (s, 3H, **21**), 1.38 (s, 12H), 1.25 (s, 12H). **^{13}C NMR** (126 MHz, $CDCl_3$) δ 152.7 (C), 152.7 (C), 142.8 (C), 142.6 (C), 141.6 (C), 140.3 (C), 139.0 (C), 138.2 (C), 129.54 (CH), 128.7 (2 x CH), 128.3 (2 x CH), 128.2 (2 x CH), 128.1 (2 x CH), 128.1 (2 x CH), 127.9 (2 x CH), 127.3 (CH), 126.9 (2 x CH), 126.7 (CH), 126.3 (CH), 126.2 (2 x CH), 83.5 (CH), 83.2 (CH), 68.3 (CH_2), 59.8 (CH_2) , 37.5 (CH_2), 37.3 (CH_2), 24.8 (4 x CH_3), 24.7 (4 x CH_3), 18.5 (CH_3), 18.3 (CH_3). **^{11}B NMR** (160 MHz, $CDCl_3$) δ 30.81. **HRMS** (APCI) m/z: [$M-H_2O+H^+$]: Calc. for $C_{25}H_{30}BO_2$ 373.2333; found 373.2335.

(2*Z*,5*Z*)-2,5-Diphenylhepta-2,5-dien-1-ol (24)

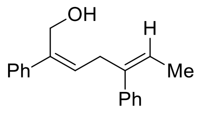

Synthesized from **2**, **23** and B_2pin_2 according to general procedure B. Yellow oil obtained in 19% yield (55.9 mg) after column chromatography (Hexane / EtOAc 4:1).

^{1}H-NMR (500 MHz, $CDCl_3$) δ 7.44-7.20 (m, 10H), 5.92 (t, *J*= 7.9 Hz, 1H), 5.67 (m, 1H), 4.45 (s, 2H), 3.33 (d, *J*= 8Hz, 2H), 1.61 (d, *J*=7 Hz, 3H). **^{13}C NMR** (126 MHz, $CDCl_3$) 141.0 (C), 140.7 (C), 139.9 (C), 139.8 (C), 129.6 (CH), 128.7 (CH), 128.5 (CH), 128.4 (CH), 128.3 (CH), 128.3 (CH), 128.1 (CH), 128.0 (CH), 127.2 (CH), 126.8 (CH), 126.3 (CH), 122.6 (CH), 59.7 (CH_2), 38.2 (CH_2), 24.7 (CH_3). **HRMS** (APCI) m/z: [$M-H_2O+H^+$]: Calc. for $C_{19}H_{19}$ 247.1480; found 247.1480.

(2*Z*,5*E*,8*Z*)-5-Methyl-2,6,9-triphenyldeca-2,5,8-triene-1,10-diol (25)

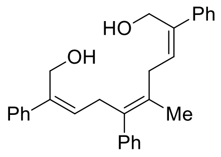

Synthesized from **2**, **23** and B_2pin_2 according to general procedure B. Yellow oil obtained in 43% yield (188.6 mg) after column chromatography (Hexane / EtOAc 4:1).

^{1}H-NMR (500 MHz, $CDCl_3$) δ 7.49 (m, 2H), 7.39-7.36 (m, 8H), 7.32-7.25 (m, 4H), 7.19-7.17 (m, 2H), 5.95 (t, *J* = 7.2 Hz, 1H), 5.82 (t, *J* = 7.4 Hz, 1H), 4.69 (s, 2H), 4.37 (s, 2H), 3.45 (d, *J*=7.4 Hz, 2H), 3.31(d, *J*=7.4 Hz, 2H), 1.67 (s, 3H). **^{13}C-NMR** (126 MHz, $CDCl_3$) δ 143.1 (C), 141.0 (C), 140.8 (C), 139.5 (C), 138.9 (C), 134.7 (C), 131.4 (C), 130.0 (CH), 129.4 (CH), 129.0 (2 x CH), 128.6 (2 x CH), 128.4 (2 x CH), 128.3 (2 x CH), 127.3 (CH), 127.1 (CH), 126.5 (CH), 126.4 (2 x CH), 126.3 (2 x CH), 59.7 (CH_2), 59.6 (CH_2), 33.8 (CH_2), 33.3 (CH_2), 20.5 (CH_3). **HRMS** (APCI) m/z: [$M-H_2O+H^+$]: Calc. for $C_{29}H_{29}O$ 393.2297; found 393.2211.

6. Synthetic modifications

6.1 Rhenium-catalyzed allylic [1,3] transposition: synthesis of dihydroborininols

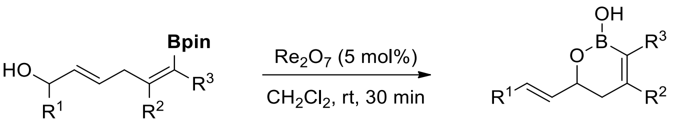

Following described procedures for the rhenium-catalyzed [1, 3]-transposition of allylic alcohols,[5] a solution of the corresponding bifunctional skipped diene (0.2 mmol, 1 equiv) in 1 ml of dry dichloromethane was stirred in the presence of Re_2O_7 (4.85 mg, 5 mol%) under Argon atmosphere for 30 minutes. After this time, the solvent was evaporated, and the mixture was filtered through a small portion of silica gel to afford the corresponding dihydroborininol.

Note: these compounds were not stable enough for HRMS analysis.

(*E*)-5,6-Diphenyl-3-styryl-3,4-dihydroborinin-1(2H)-ol (26)

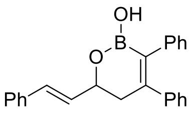

Obtained from diene **8** as a brown oil (57.7 mg, 82% yield).

^{1}H NMR (300 MHz, $CDCl_3$) δ 7.47 – 7.34 (m, 5H), 7.26 – 7.16 (m, 5H), 7.12 – 7.02 (m, 5H), 6.79 (d, *J* = 15.9 Hz, 1H), 6.41 (dd, *J* = 15.9, 5.8 Hz, 1H), 5.05 (dt, *J* = 10.4, 5.1 Hz, 1H), 3.06 – 2.82 (m, 2H). **^{13}C NMR** (75 MHz, $CDCl_3$) δ 153.6 (C), 141.1 (C), 139.9 (C), 136.7 (C), 130.8 (CH), 130.4 (C) 129.8 (CH), 129.4 (2 x CH), 128.6 (2 x CH), 128.3 (2 x CH), 128.2 (2 x CH), 127.9 (2 x CH), 127.7 (CH) 127.4 (CH), 126.6 (2 x CH), 126.1 (CH), 73.68 (CH), 40.5 (CH_2). **^{11}B NMR** (160 MHz, $CDCl_3$) δ 22.45.

3-Methyl-4-(p-tolyl)-6-vinyl-5,6-dihydro-2H-1,2-oxaborinin-2-ol (27)

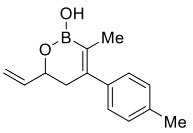

Obtained from diene **4** as a brown oil (19 mg, 41% yield).

^{1}H NMR (500 MHz, $CDCl_3$) δ 7.21 – 7.19 (m, 2H), 7.13 – 7.11 (m, 2H), 5.99 (ddd, *J* = 17.2, 10.5, 5.6 Hz, 1H), 5.37 – 5.32 (ddd, *J* = 17.2, 10.5, 5.6 Hz, 1H), 5.19 (dt, *J* = 10.5, 1.4 Hz, 1H), 4.71 – 4.66 (m, 1H), 4.27 – 4.19 (m, 1H), 2.68 – 2.54 (m, 2H), 2.39 (s, 3H), 1.78 (dd, *J* = 2.2, 1.3 Hz, 3H). **^{13}C NMR** (126 MHz, $CDCl_3$) δ 152.1 (C), 138.9 (CH), 138.6 (C), 136.9 (C), 130.9 (C), 128.83 (2 x CH), 127.5 (2 x CH), 115.3 (CH_2), 74.1 (CH), 40.12 (CH_2), 21.21 (CH_3), 14.9 (CH_3). **^{11}B NMR** (160 MHz, $CDCl_3$) δ 27.95.

6.2 One-pot allylic [1,3] transposition/Suzuki cross-coupling

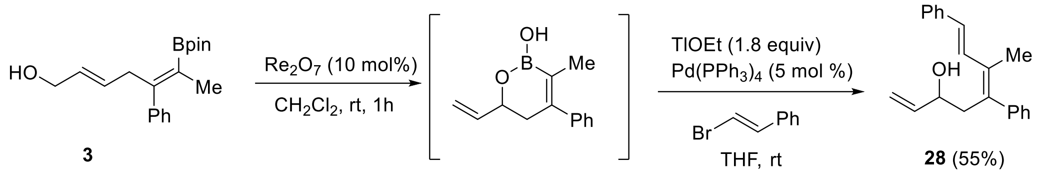

A solution of the difunctionalized diene **3** (62.8 mg, 0.2 mmol, 1 equiv) in 1 ml of dry dichloromethane was stirred in the presence of Re_2O_7 (9.7 mg, 10 mol%) under Argon atmosphere for 1 h. After this time, the solvent was evaporated under vacuum. Then, a solution of β-bromostyrene (43.95 mg, 0.24 mmol, 1.2 equiv) in 1 ml of THF/H_2O (3:1) was added, followed by the addition of $Pd(PPh_3)_4$ (11.56 mg, 5 mol%) and TlOEt (89.8 mg, 0.36 mmol, 1.8 equiv). The resulting solution was stirred at room temperature under argon atmosphere for 1 h. After this time, the mixture was diluted in EtOAc and washed with brine. The organic layer was dried, filtered and the solvents were evaporated under vacuum. The final product was purified by SiO_2 column chromatography using a mixture of Hexane : AcOEt (8:2) as eluent to afford the product **28** as a yellow oil in 55% yield (32.0 mg).

(5*E*,7*E*)-6-Methyl-5,8-diphenylocta-1,5,7-trien-3-ol (28)

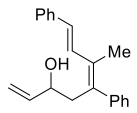

^{1}H NMR (500 MHz, $CDCl_3$) δ 7.50 – 7.47 (m, 1H), 7.45 (d, *J* = 15.9 Hz, 1H), 7.40 – 7.34 (m, 4H), 7.31 – 7.28 (m, 1H), 7.26 – 7.23 (m, 1H), 7.21 – 7.18 (m, 2H), 6.73 (d, *J* = 15.9 Hz, 1H), 5.91 (ddd, *J* = 17.2, 10.5, 5.8 Hz, 1H), 5.23 (dt, *J* = 17.2, 1.5 Hz, 1H), 5.10 (dt, *J* = 10.5, 1.4 Hz, 1H), 4.12– 4.07 (m, 1H), 3.04 (dd, *J* = 13.9, 8.2 Hz, 1H), 2.82 (dd, *J* = 13.9, 8.2 Hz, 1H), 1.85 (s, 3H). **^{13}C NMR** (126 MHz, $CDCl_3$) δ 142.2 (C), 139.3 (CH), 136.9 (C), 136.4 (C), 131.1 (C), 128.6 (CH), 127.9 (2 x CH), 127.6 (2 x CH), 127.2 (2 x CH), 126.7 (CH), 126.4 (CH), 125.7 (CH), 125.5 (2 x CH), 113.7 (CH_2), 70.4 (CH), 40.9 (CH_2), 15.8 (CH_3). **HRMS** (APCI) m/z: [M-H_2O+H^+]: Calc. for $C_{21}H_{21}$ 273.1614; found 273.1610.

6.3 Oxidation[6]: Synthesis of 6-hydroxy-ketones

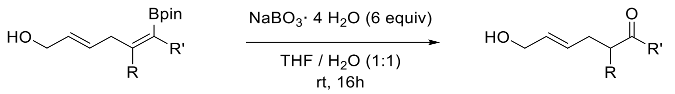

A solution of the corresponding borylated diene (0.2 mmol, 1 equiv) in 1 ml of THF and 1 ml of water was stirred in the presence of $NaBO_3·4H_2O$ (1.2 mmol, 6 equiv) over 16 hours. After this time, the reaction mixture was diluted in Et_2O and washed with brine. The organic layer was dried, filtered and the solvents were evaporated under vacuum. The final product was purified by SiO_2 column chromatography using Hexane / AcOEt (4:1) as eluent to afford the corresponding ketone.

(*E*)-6-Hydroxy-1,2-di-p-tolylhex-4-en-1-one (29)

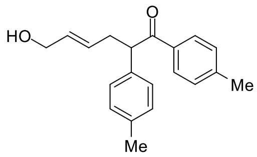

Obtained from diene **5** as a yellow oil (38.3 mg, 65% yield).

^{1}H NMR (300 MHz, $CDCl_3$) δ 7.87 (d, *J* = 8.1 Hz, 2H), 7.18 (d, *J* = 8.7 Hz, 4H), 7.10 (d, *J* = 8.1 Hz, 2H) 5.71 – 5.56 (m, 2H), 4.58 (t, *J* = 7.5 Hz, 1H), 4.03 (s, 2H), 3.02 – 2.87 (m, 1H), 2.60 – 2.54 (m, 1H), 2.36 (s, 3H), 2.29 (s, 3H). **^{13}C NMR** (75 MHz, $CDCl_3$) δ 198.8 (C), 143.7 (C), 136.7 (C), 136.2 (C), 134.1 (C), 131.1, 130.4 (CH), 129.6 (2 x CH), 129.2 (2 x CH), 128.9 (2 x CH), 128.0 (2 x CH), 63.6 (CH_2), 53.1 (CH), 36.6 (CH_2), 21.6 (CH_3), 21.0 (CH_3). **HRMS** (APCI) m/z: [$M-H_2O+H^+$]: Calc. for $C_{20}H_{21}O$ 277.1587; found 277.1587.

(*E*)-6-Hydroxy-2-(4-methoxyphenyl)-1-(4-(trifluoromethyl)phenyl)hex-4-en-1-one (46)

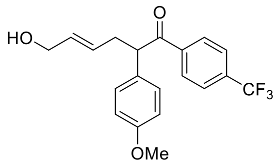

Synthesized from a 2:1 mixture of compounds **6** and **6'**. Formation of a 2:1 mixture of ketones **46** and **46'** was observed by NMR analysis of the reaction crude. After column chromatography, it was possible to isolate the pure major product **46** as a yellow oil in 22% yield (15.0 mg) (see section 9 for further information).

^{1}H NMR (300 MHz, $CDCl_3$) δ 8.04 (d, *J* = 8.1 Hz, 2H), 7.67 (d, *J* = 8.2 Hz, 2H), 7.25 – 7.15 (m, 2H), 6.86 (d, *J* = 8.6 Hz, 2H), 5.67 (q, *J* = 4.9 Hz, 2H), 4.55 (t, *J* = 7.3 Hz, 1H), 4.06 (d, *J* = 4.2 Hz, 2H), 3.78 (s, 3H), 2.95 (dt, *J* = 13.2, 6.3 Hz, 1H), 2.57 (dt, *J* = 13.3, 6.2 Hz, 1H). **^{13}C NMR** (75 MHz, $CDCl_3$) δ 198.3 (C), 158.9 (C), 131.5 (CH), 130.2 (CH), 129.6 (CH), 129.2 (C), 128.9 (C), 125.6 (d, *J* = 3.8 Hz, 2 x CH), 114.6 (2 x CH), 63.5 (CH_2), 55.2 (CH_3), 53.4 (CH), 36.4 (CH_2). **^{19}F NMR** (282 MHz, $CDCl_3$) δ -63.22. **HRMS** (APCI) m/z: [$M-H_2O+H^+$]: Calc. for $C_{20}H_{18}F_3O_2$ 347.1253, found 347.1246.

7. Unsuccessful substrates

Although the transformation showed a broad functional group tolerance as well as the possibility to include different substitution patterns, some substrates resulted unreactive or poorly efficient under standard reaction conditions. Unsuccessful vinyl epoxides and alkynes are shown below.

7.1 Vinyl epoxides

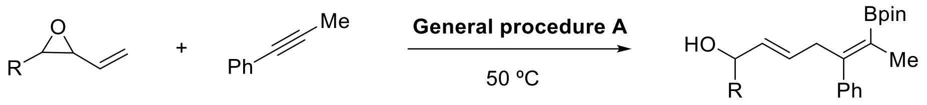

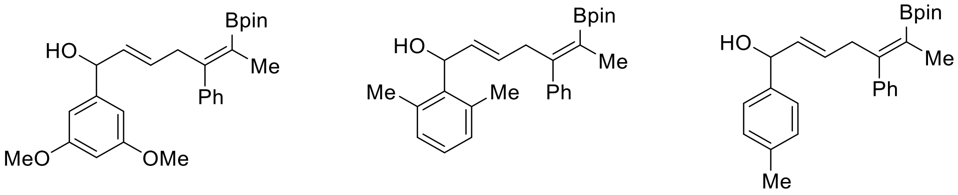

Traces | 0 % | 15%

7.2 Alkynes

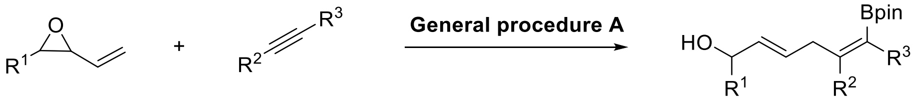

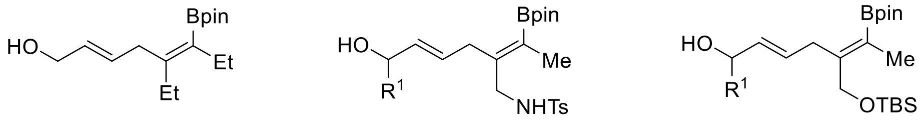

0% | R^1= Ph, H Traces | R^1= Ph, H < 20%

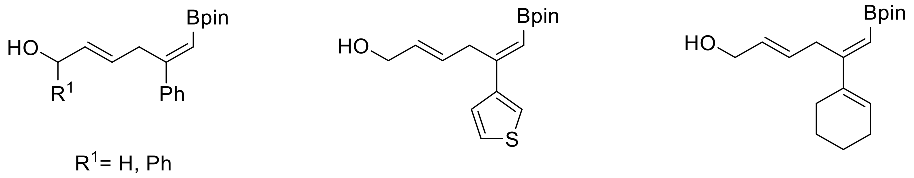

R^1= H, Ph 0% | 0% | 0%

8. NMR Spectra

^{1}H NMR (500 MHz, $CDCl_3$)

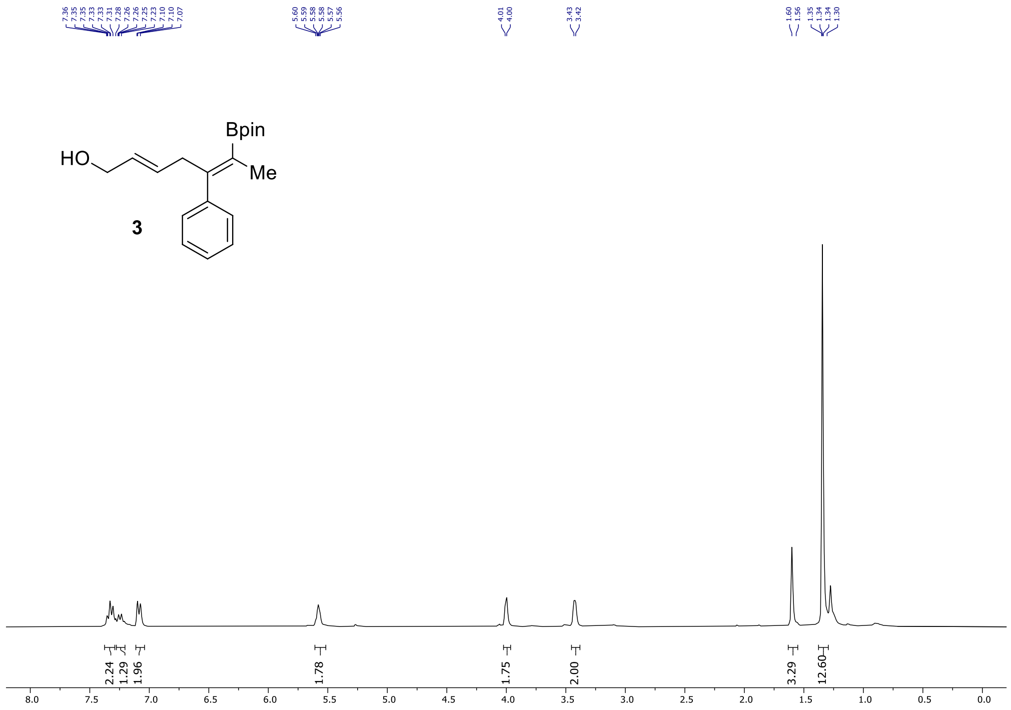

^{13}C NMR (126 MHz, $CDCl_3$)

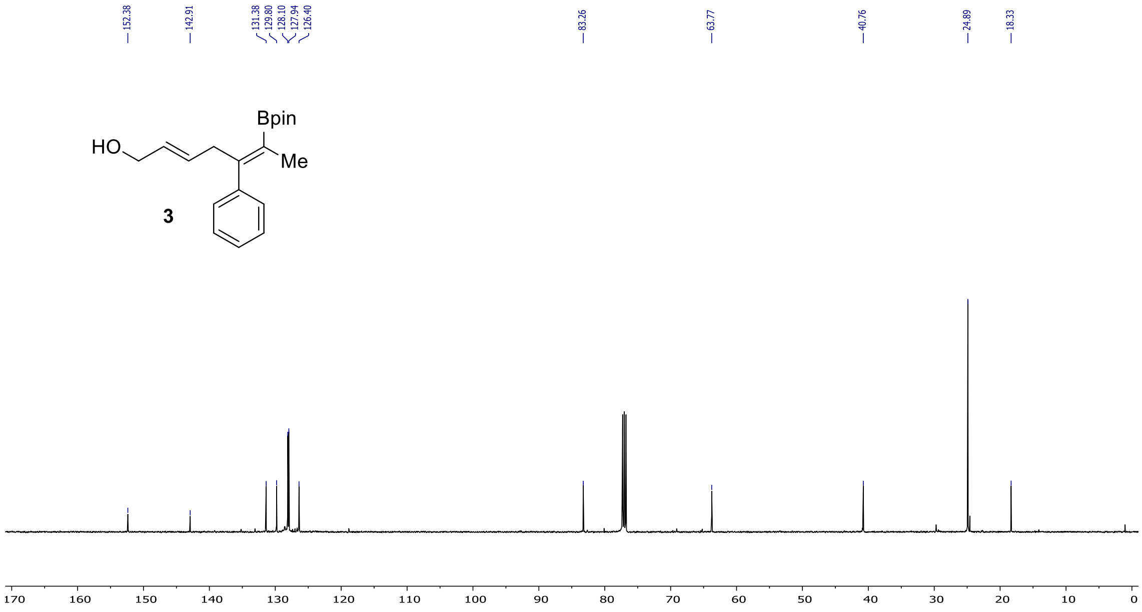

^{1}H NMR (500 MHz, $CDCl_3$)

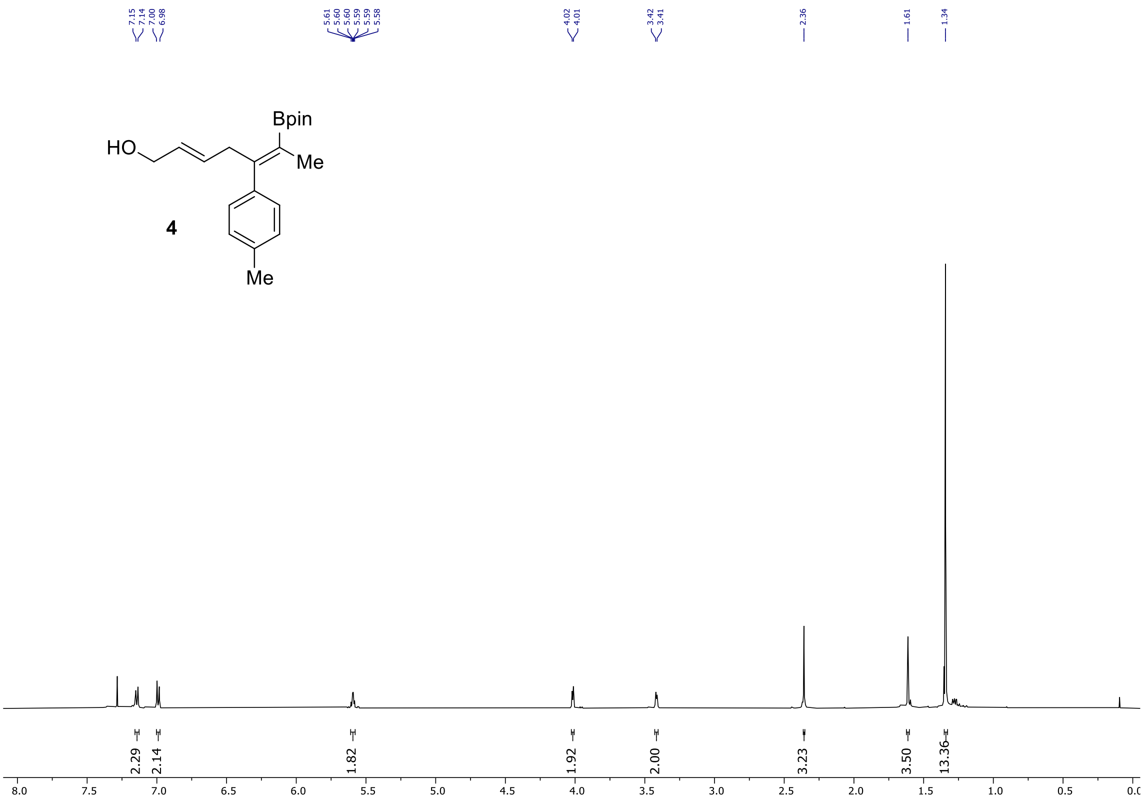

^{13}C NMR (126 MHz, $CDCl_3$)

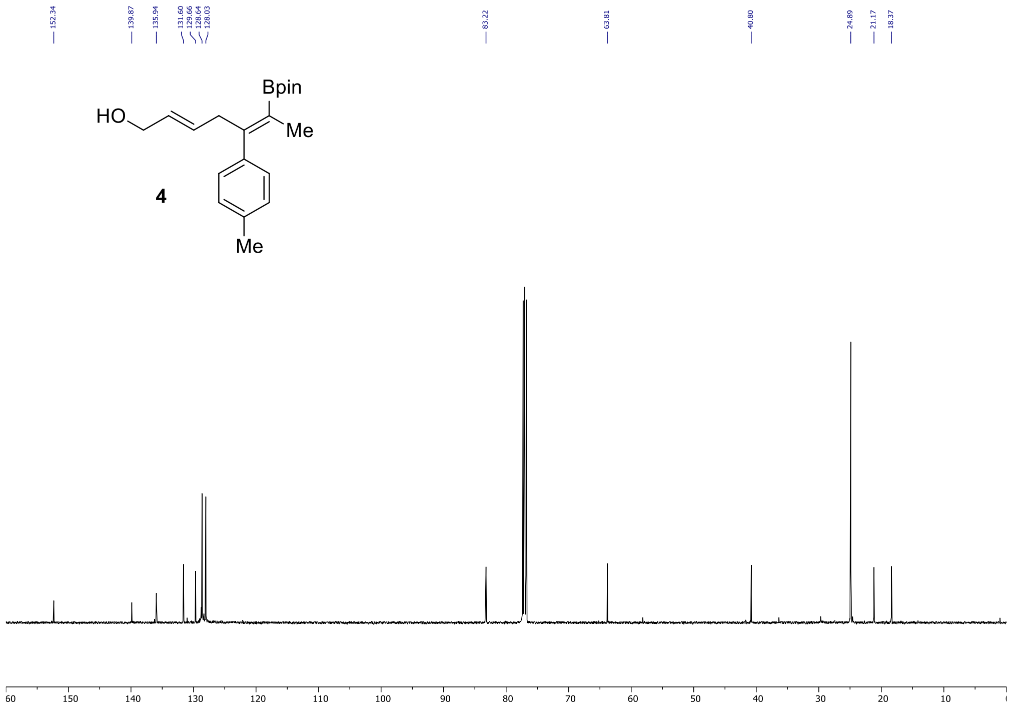

^{1}H NMR (500 MHz, $CDCl_3$)

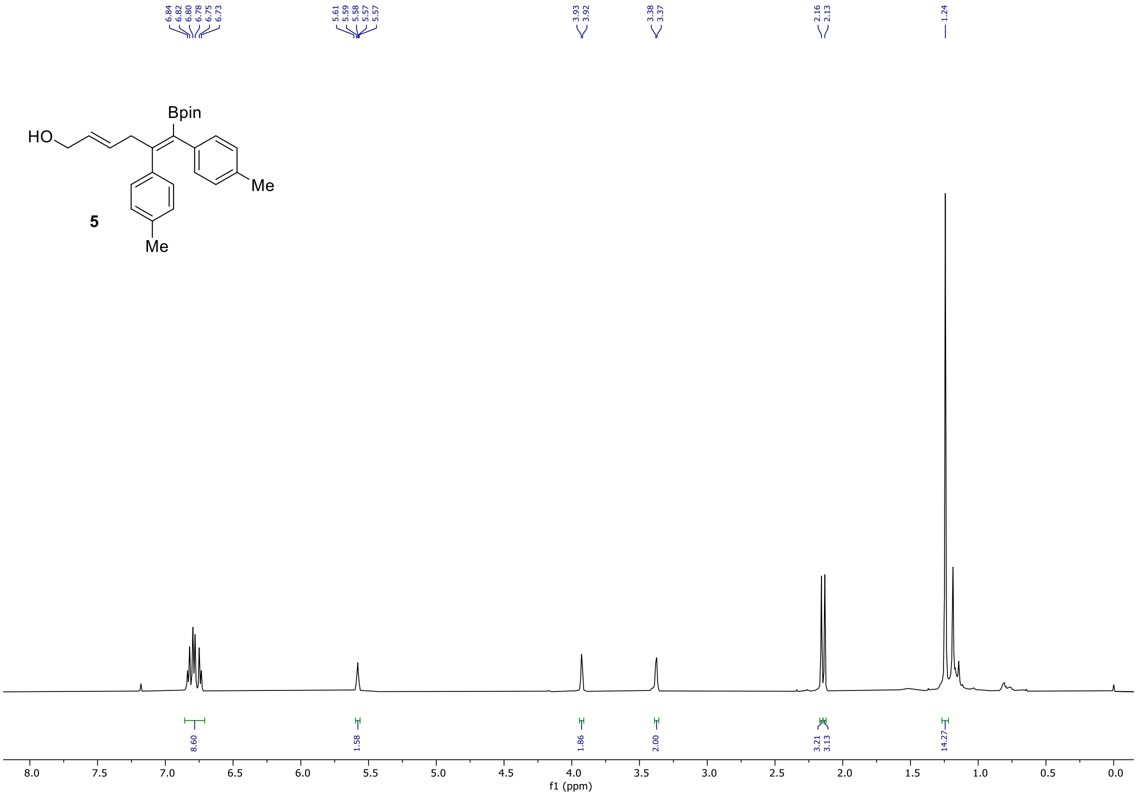

^{13}C NMR (75 MHz, $CDCl_3$)

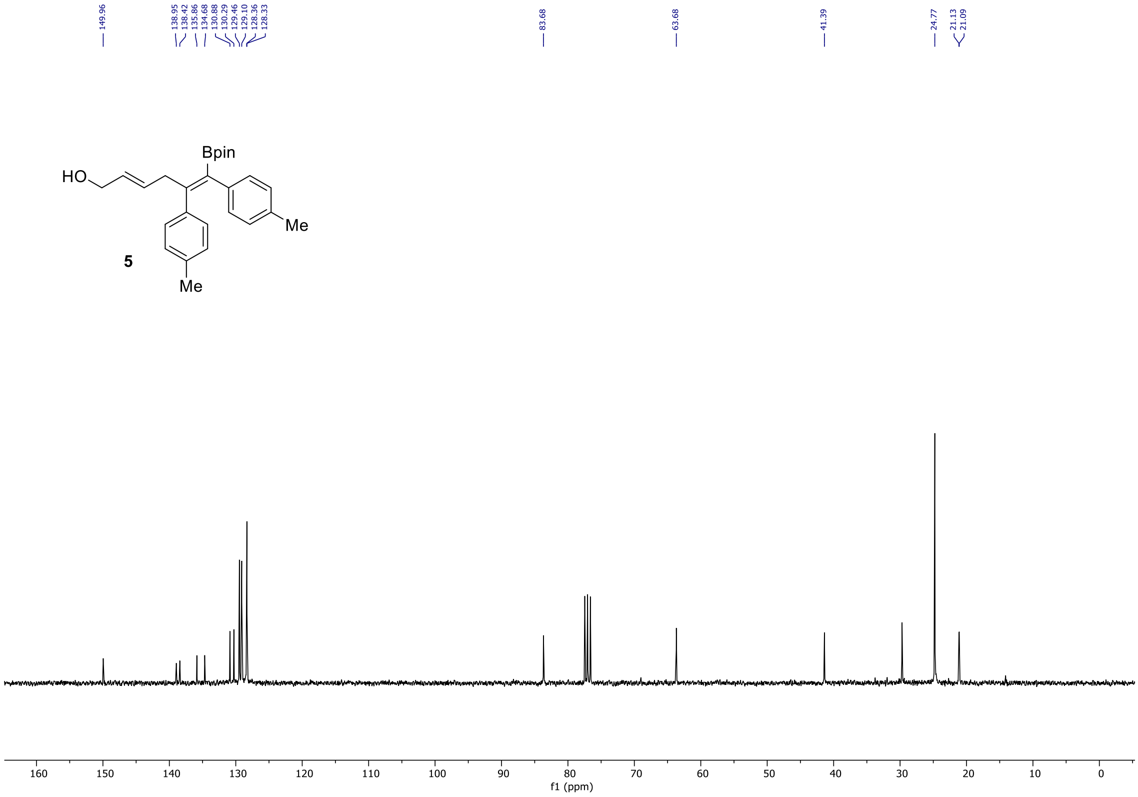

^{1}H NMR (300 MHz, $CDCl_3$)

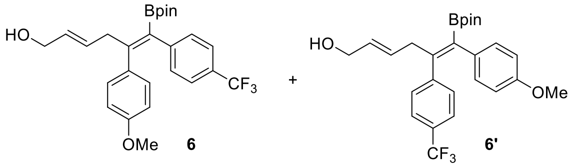

6 + **6'**

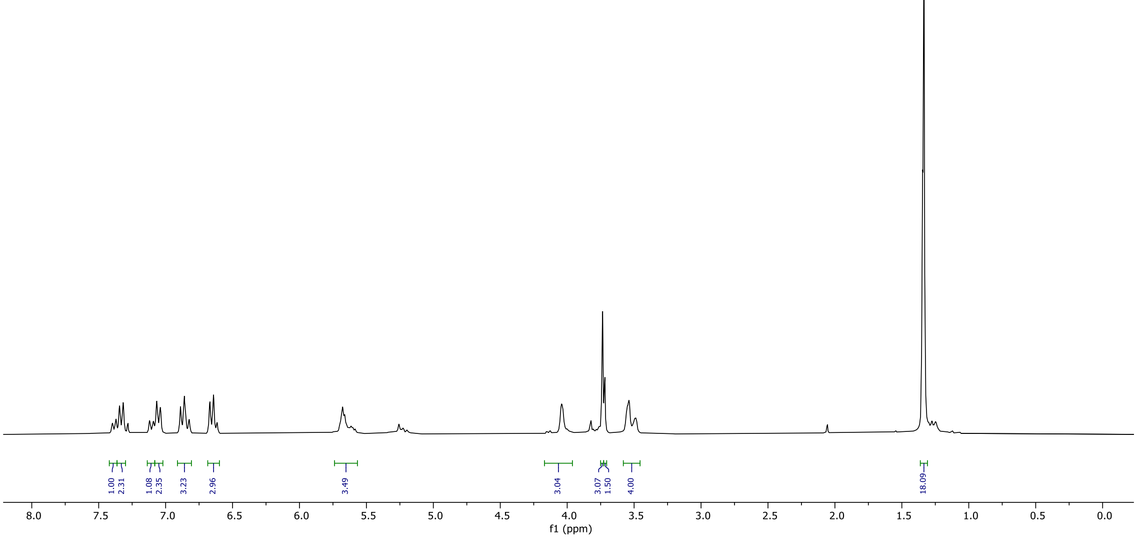

^{13}C NMR (75 MHz, $CDCl_3$)

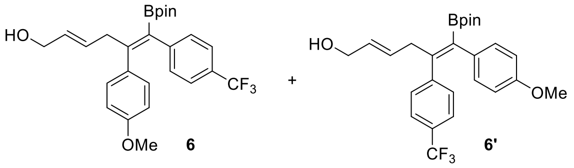

6 + **6'**

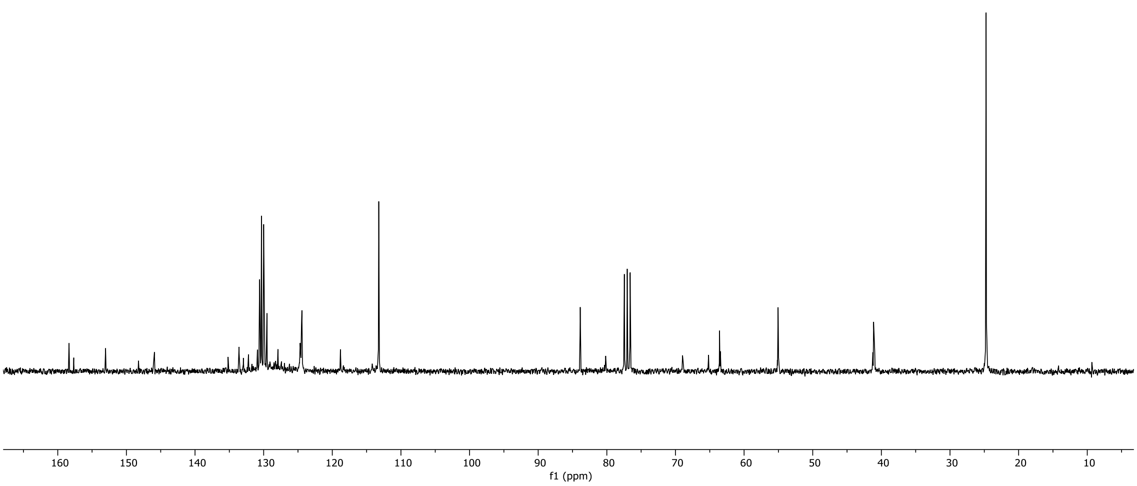

^{19}F NMR (282 MHz, $CDCl_3$)

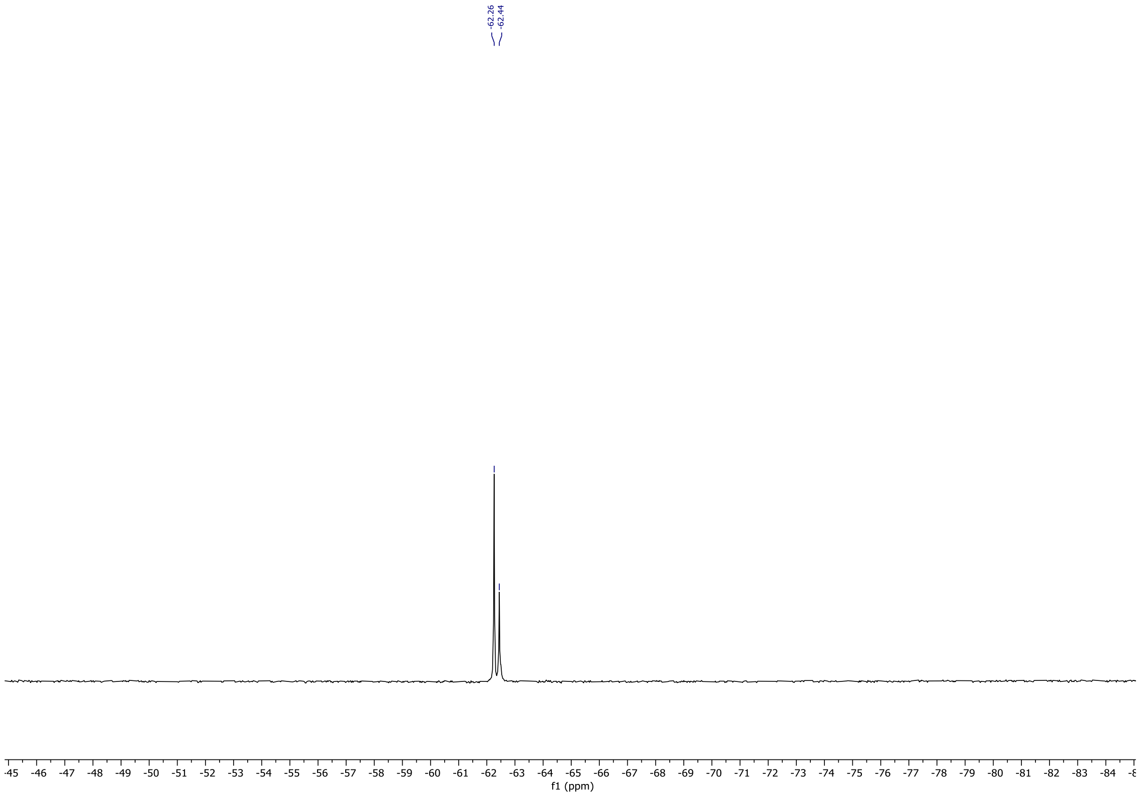

^{1}H NMR (500 MHz, $CDCl_3$)

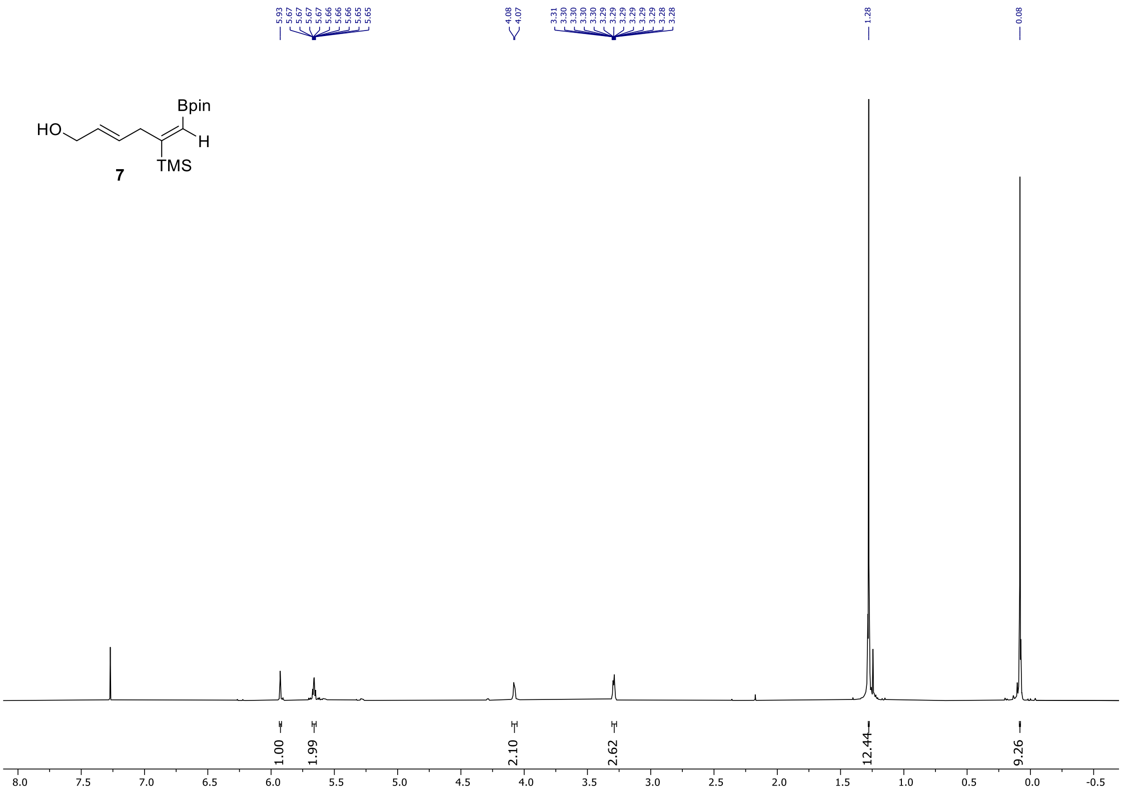

^{13}C NMR (126 MHz, $CDCl_3$)

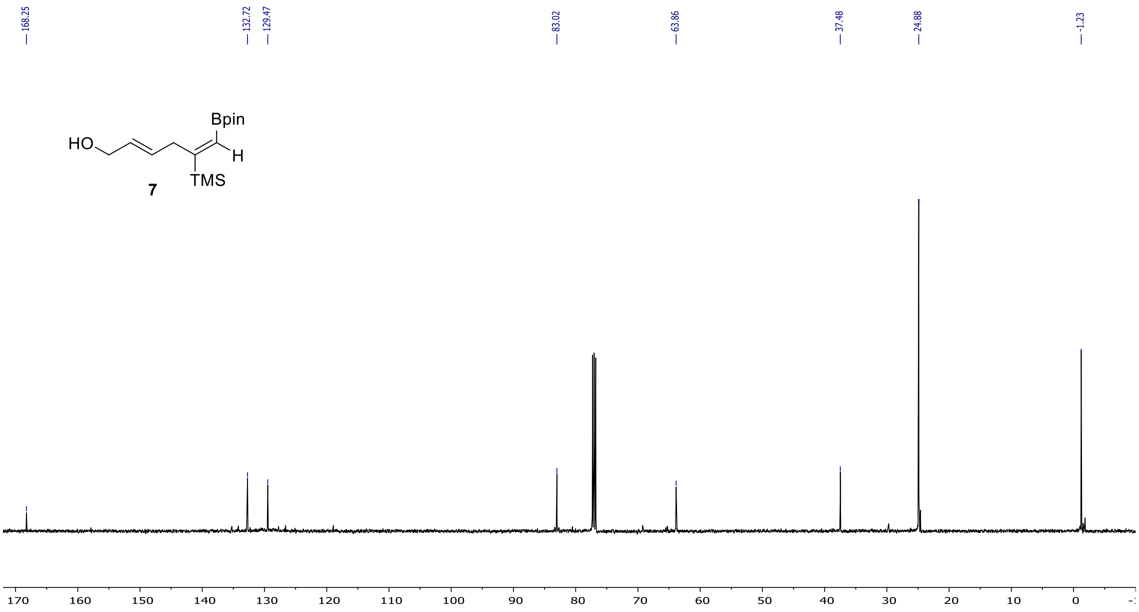

^{1}H NMR (500 MHz, $CDCl_3$)

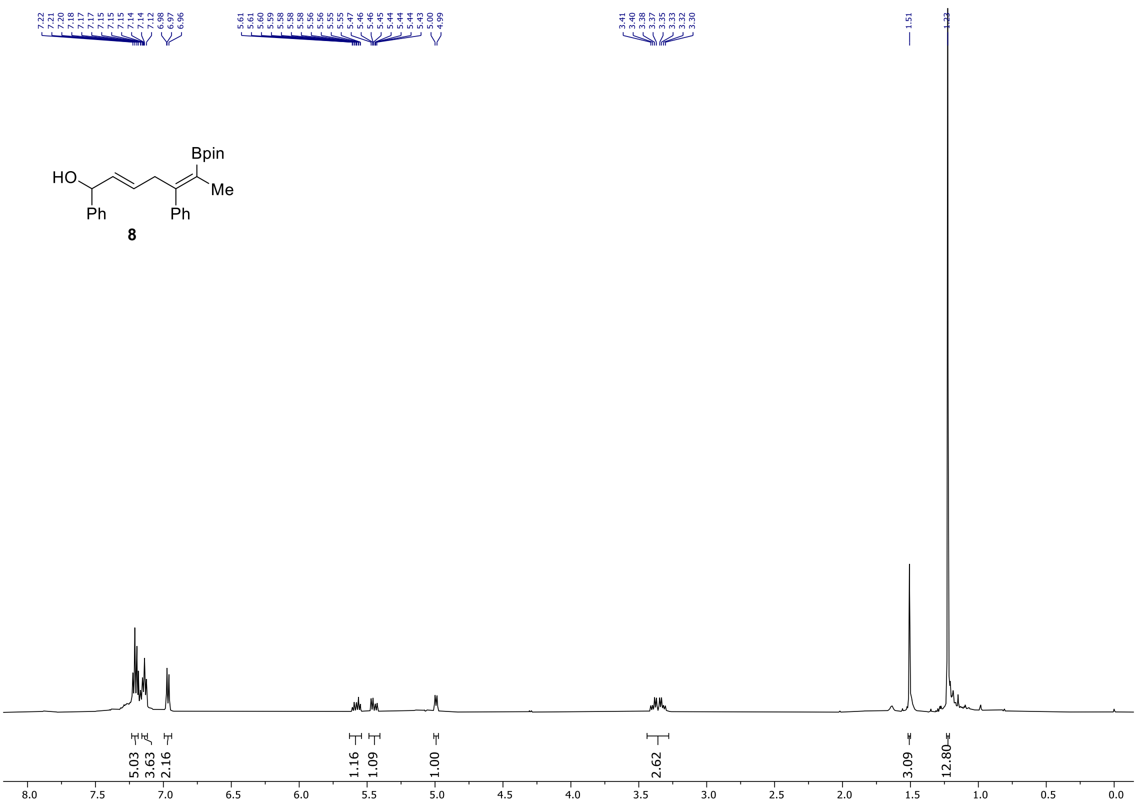

^{13}C NMR (126 MHz, $CDCl_3$)

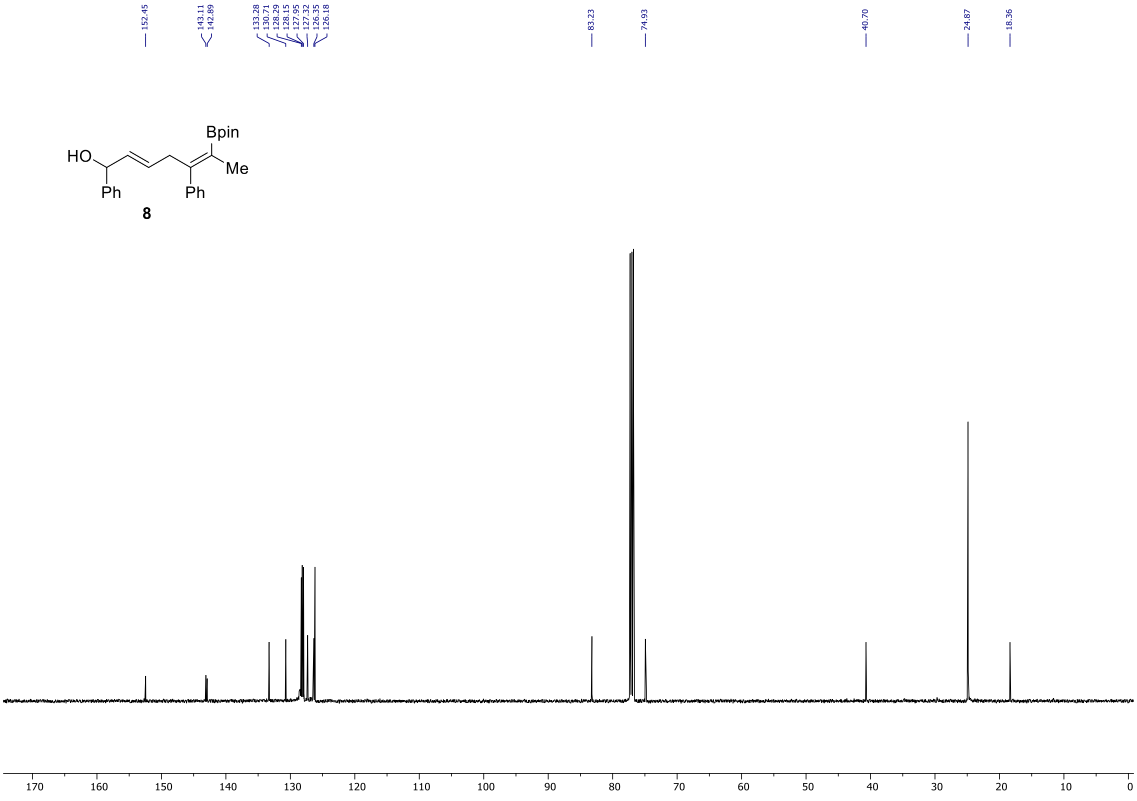

^{1}H NMR (300 MHz, $CDCl_3$)

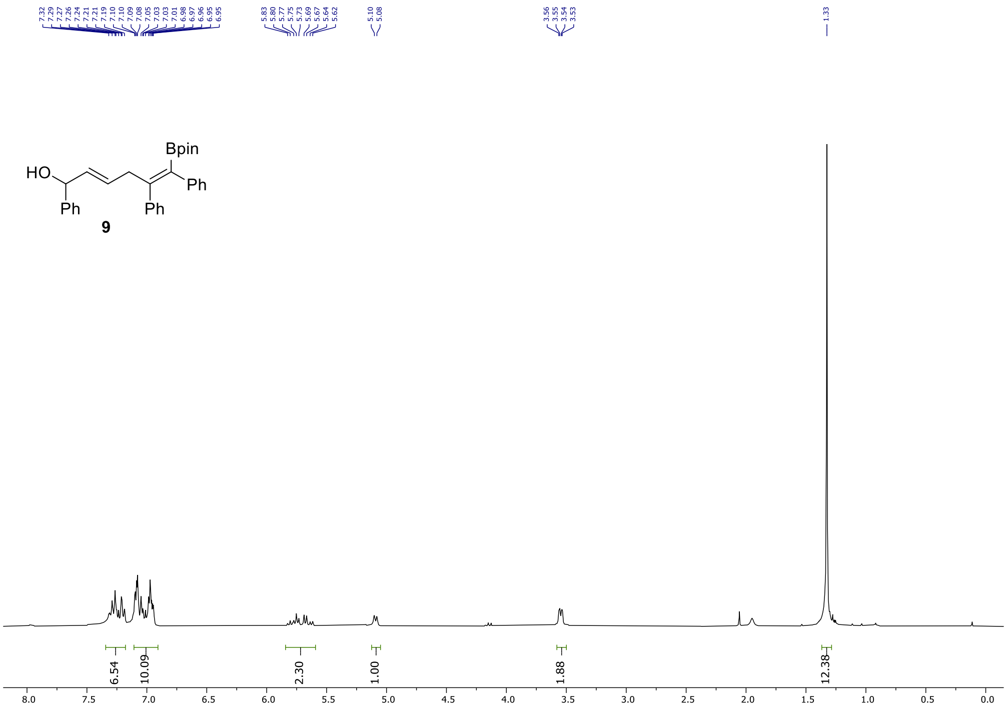

^{13}C NMR (75 MHz, $CDCl_3$)

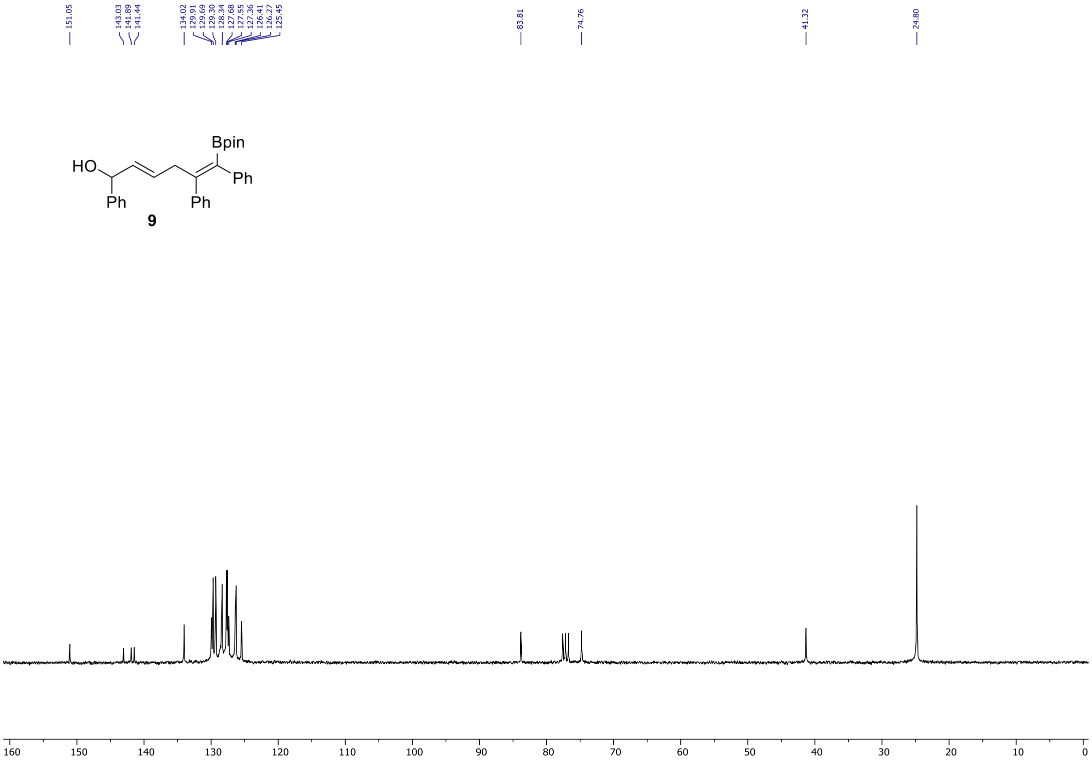

^{1}H NMR (500 MHz, $CDCl_3$)

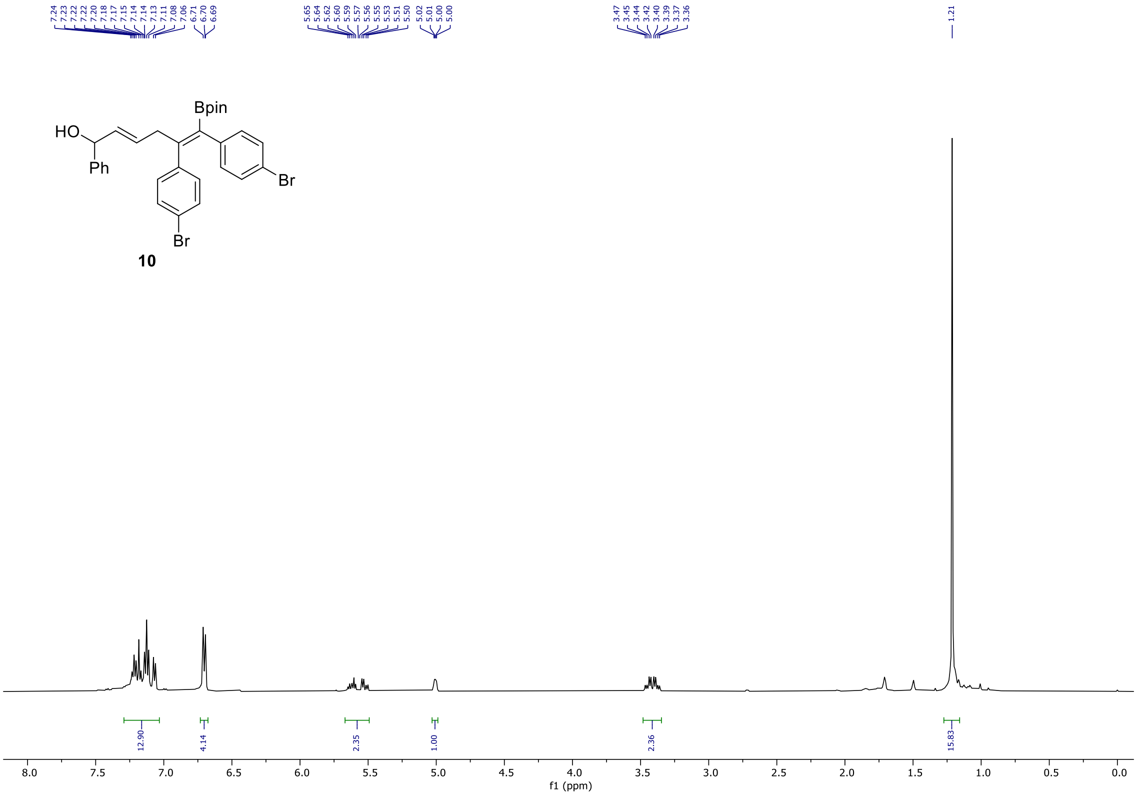

^{1}H NMR (126 MHz, $CDCl_3$)

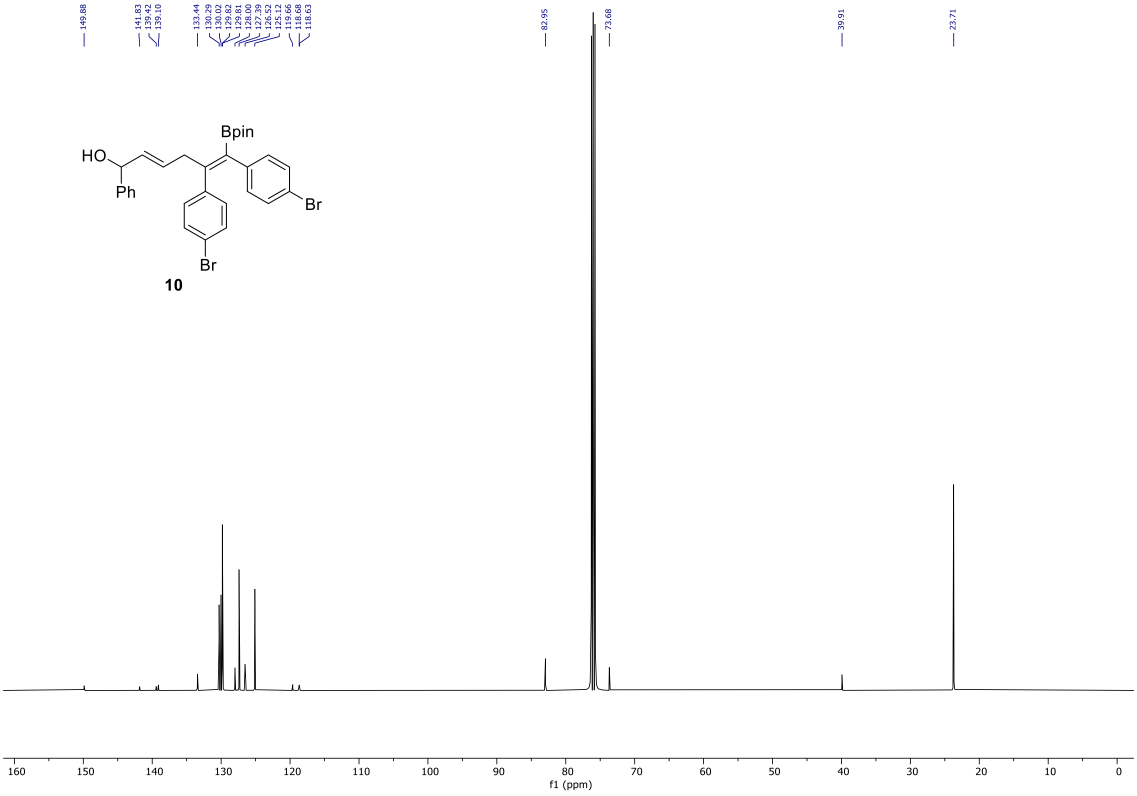

^{1}H NMR (300 MHz, $CDCl_3$)

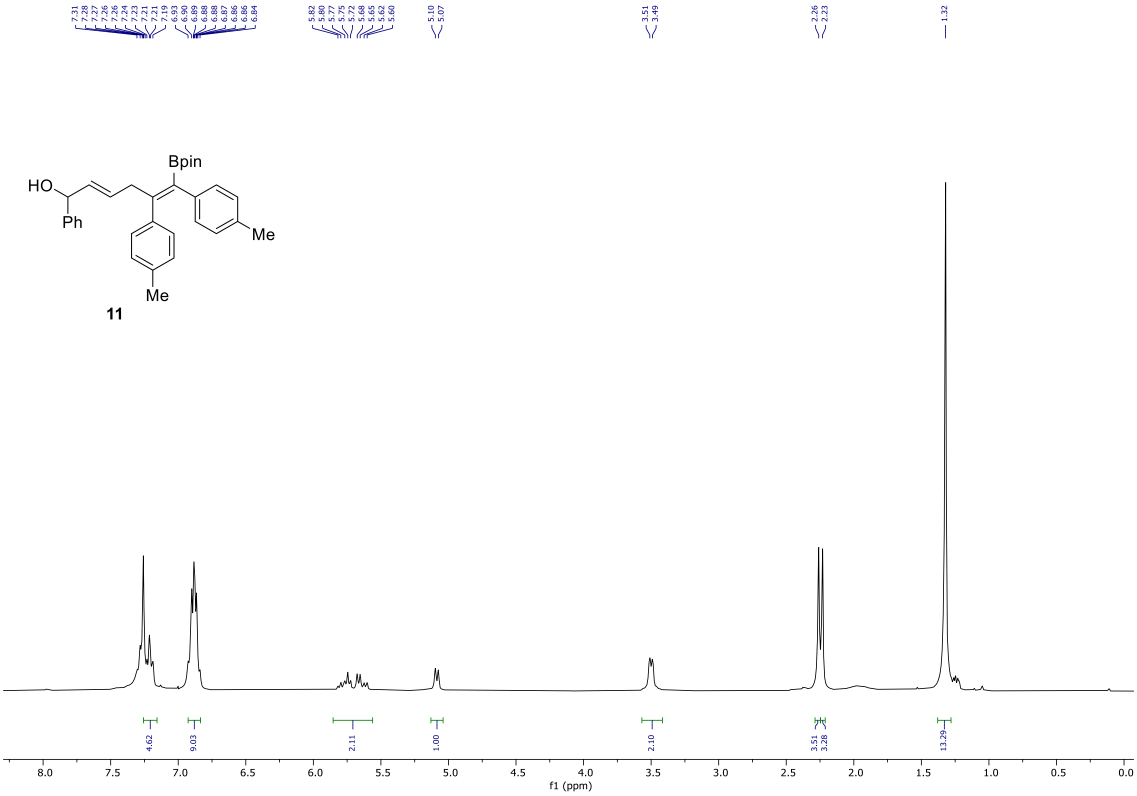

^{13}C NMR (75 MHz, $CDCl_3$)

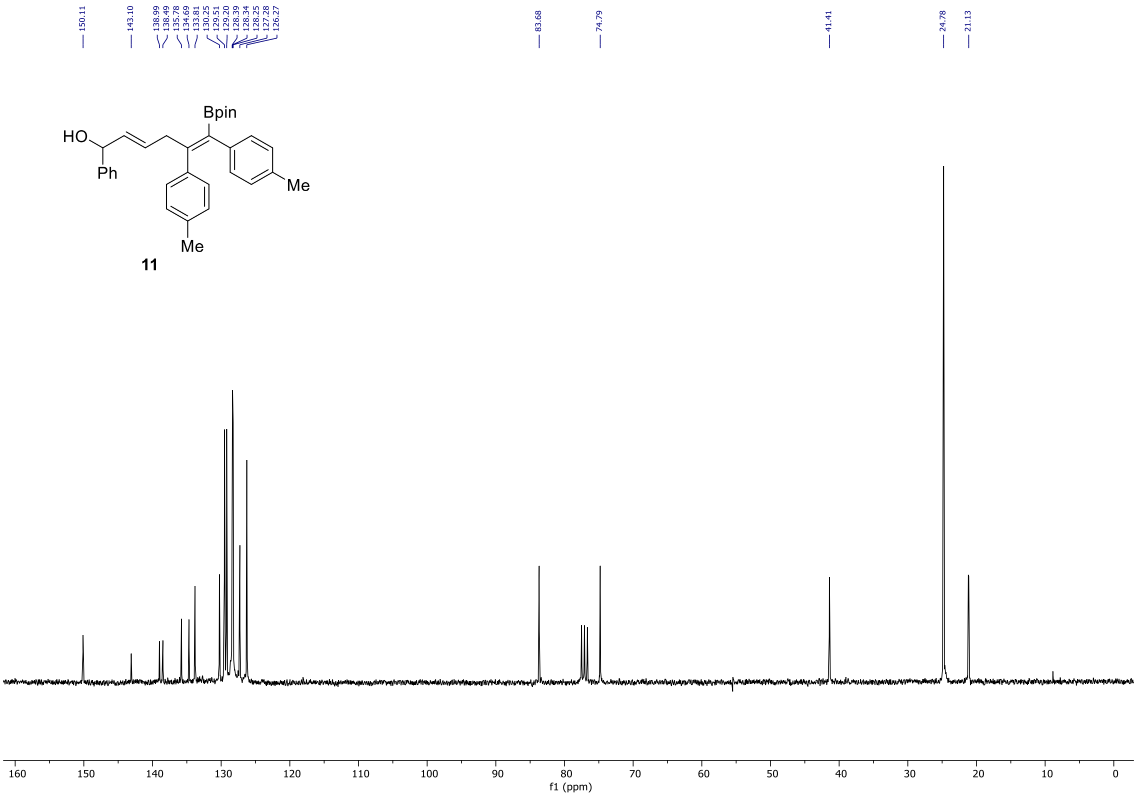

^{1}H NMR (300 MHz, $CDCl_3$)

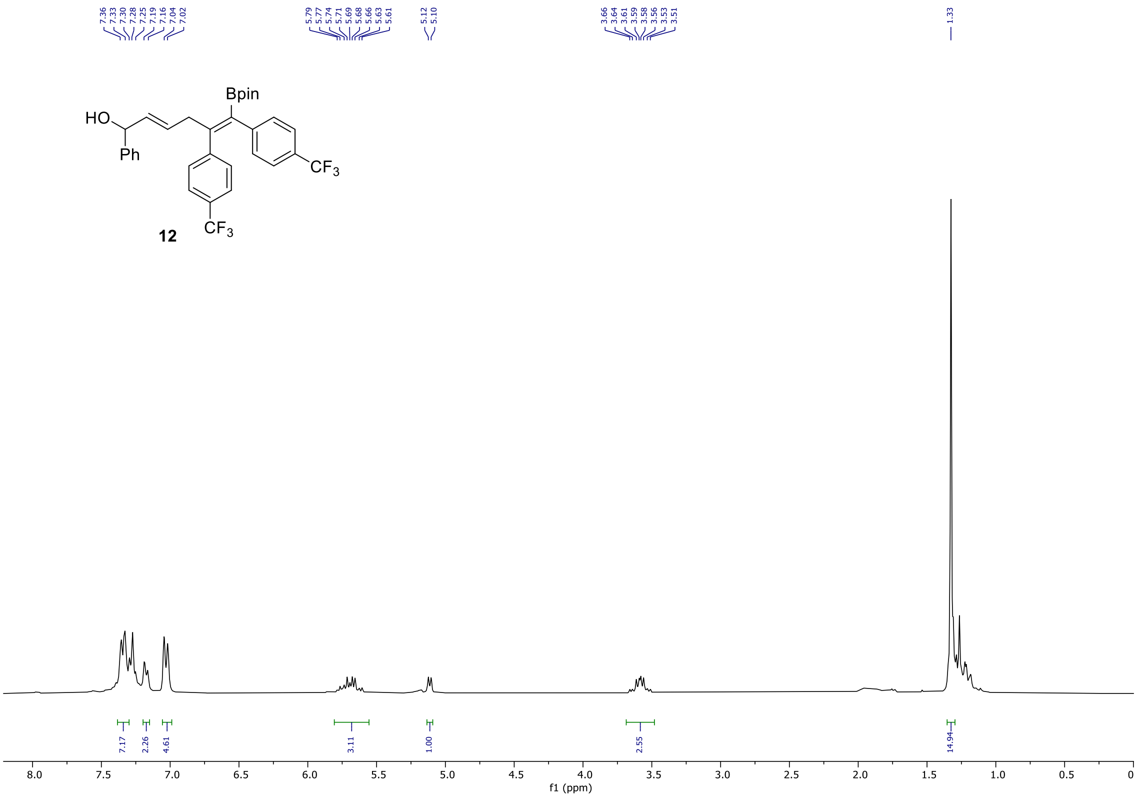

^{13}C NMR (75 MHz, $CDCl_3$)

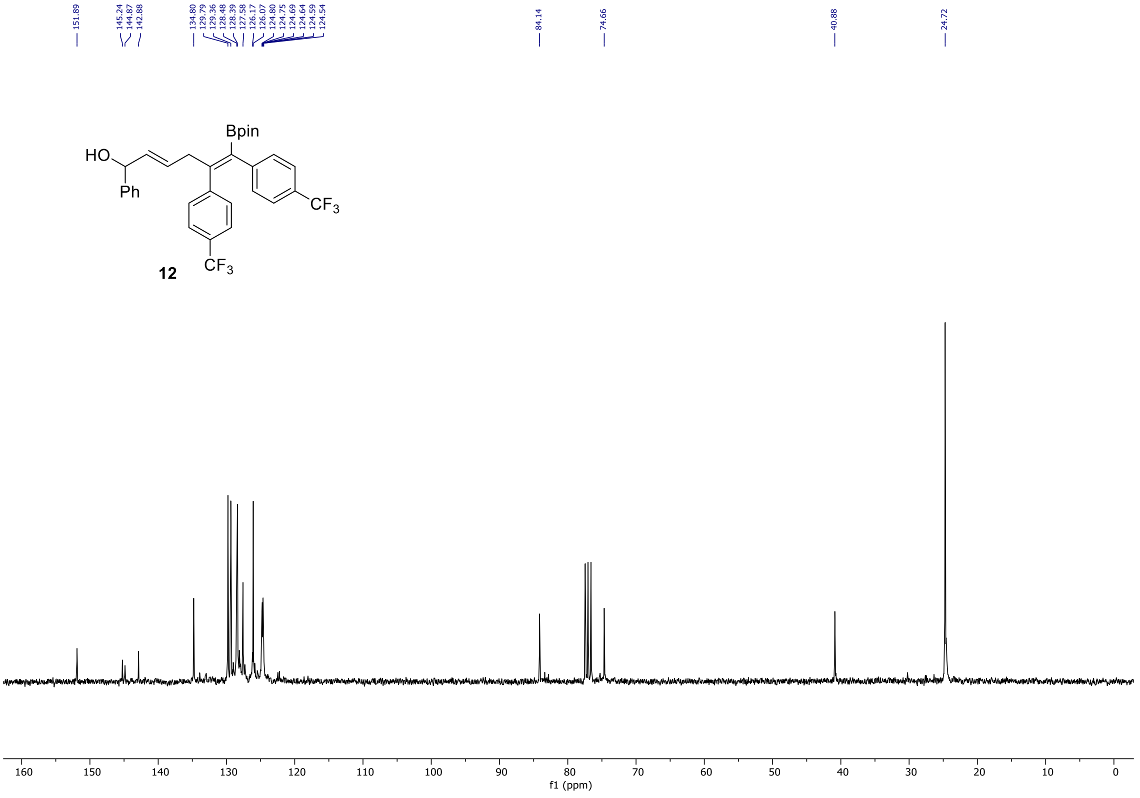

^{19}F NMR (282 MHz, $CDCl_3$)

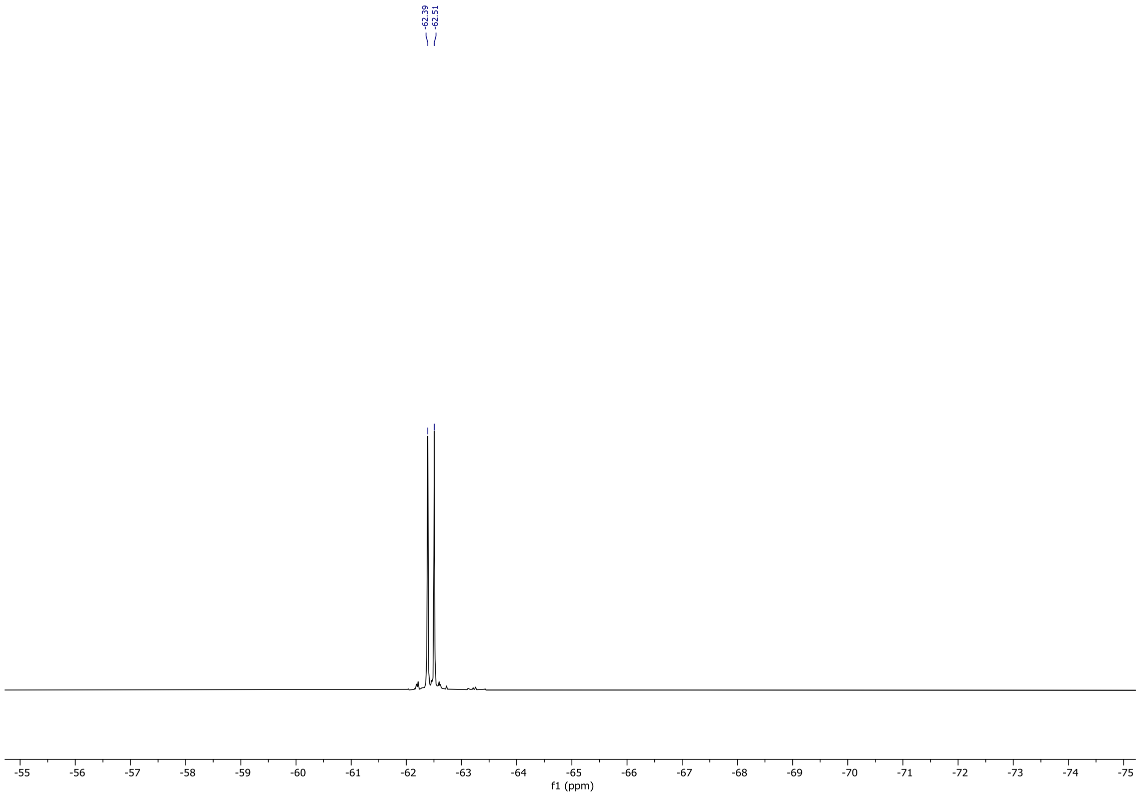

¹H NMR (500 MHz, $CDCl_3$)

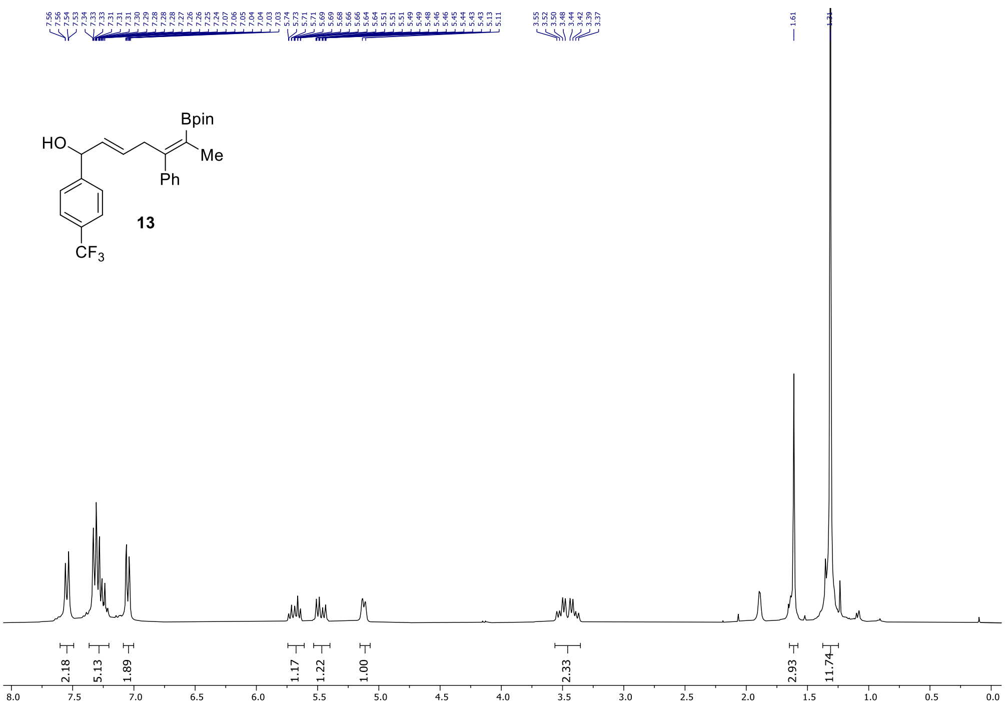

¹³C NMR (162 MHz, $CDCl_3$)

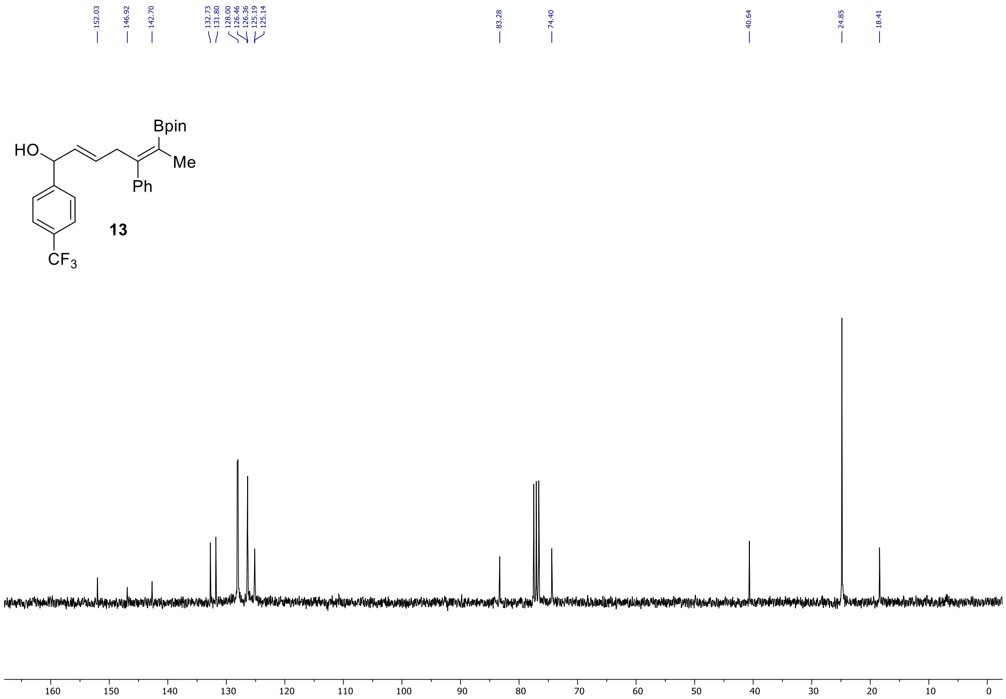

^{19}F NMR (282 MHz, $CDCl_3$)

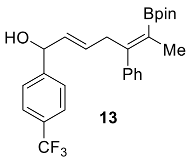

13

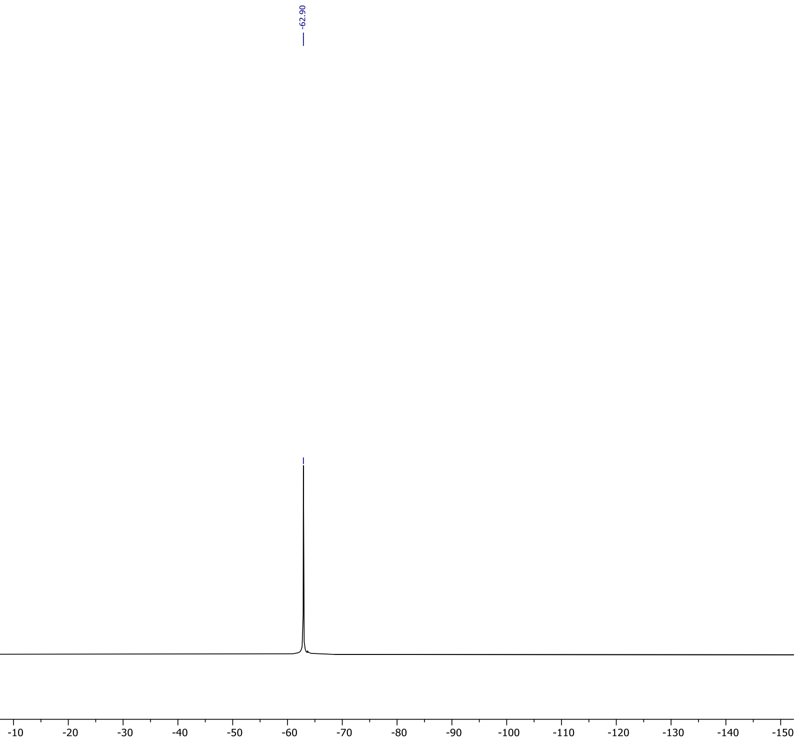

^{1}H NMR (500 MHz, $CDCl_3$)

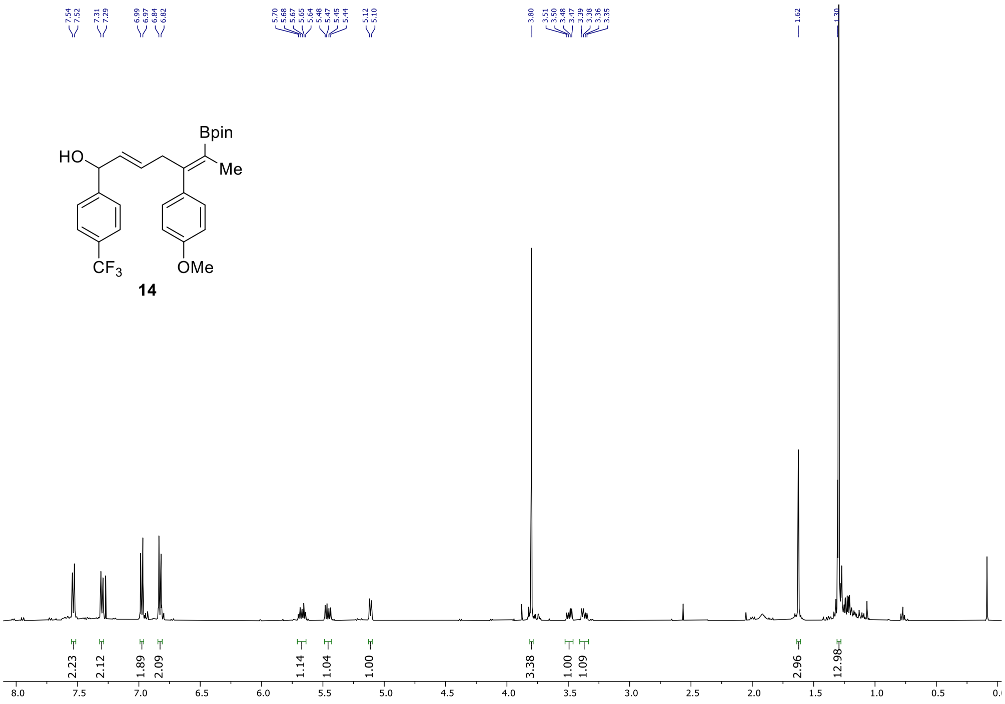

^{13}C NMR (162 MHz, $CDCl_3$)

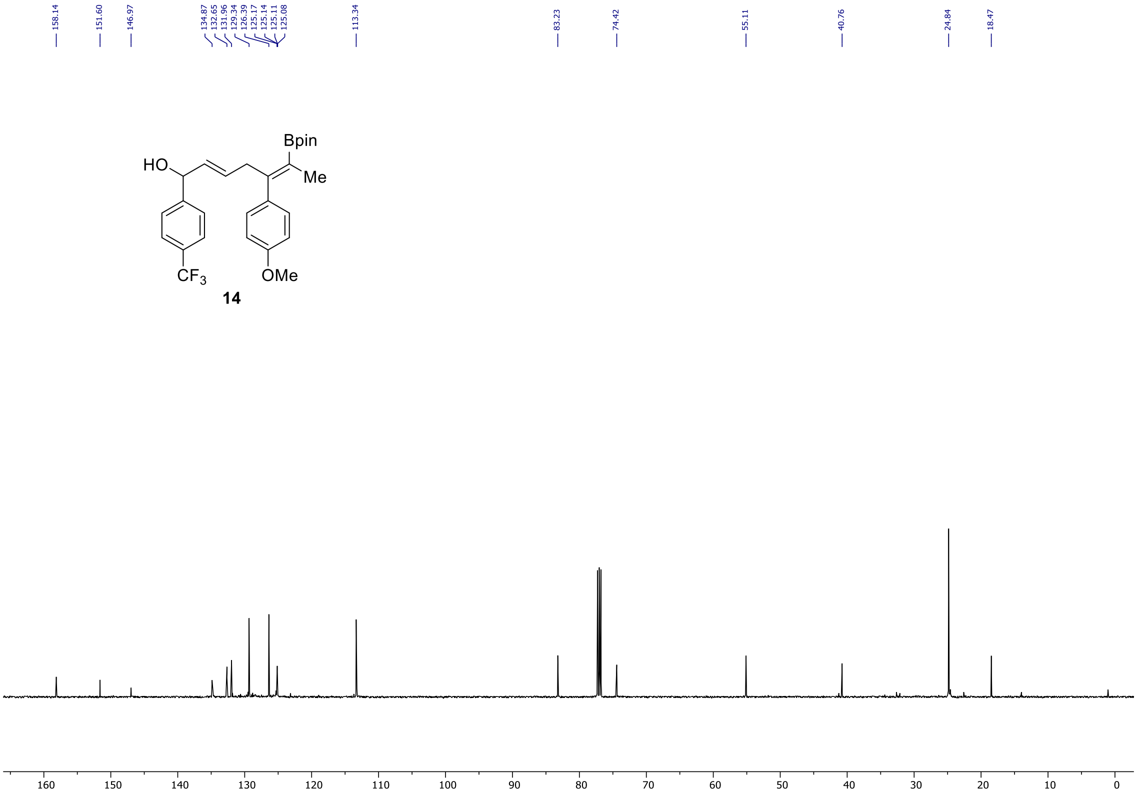

^{19}F NMR (282 MHz, $CDCl_3$)

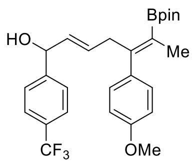

14

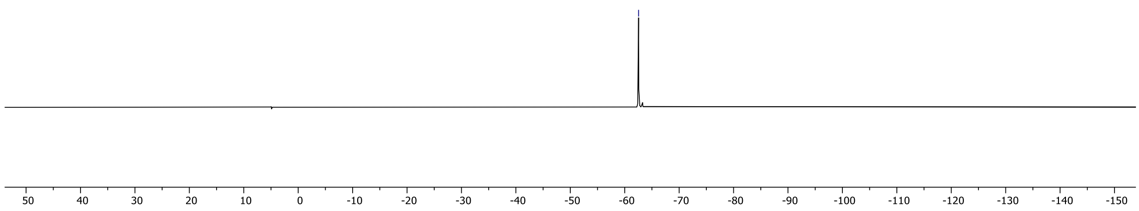

^{1}H NMR (300 MHz, $CDCl_3$)

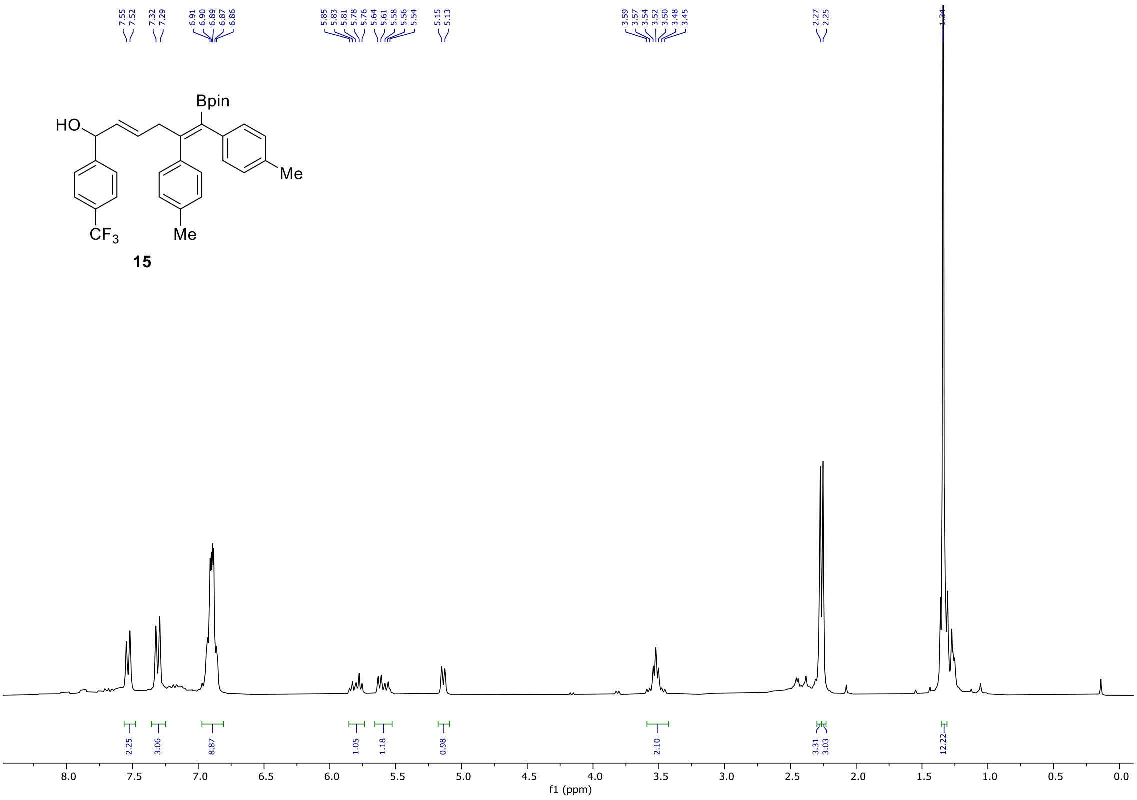

15

^{13}C NMR (75 MHz, $CDCl_3$)

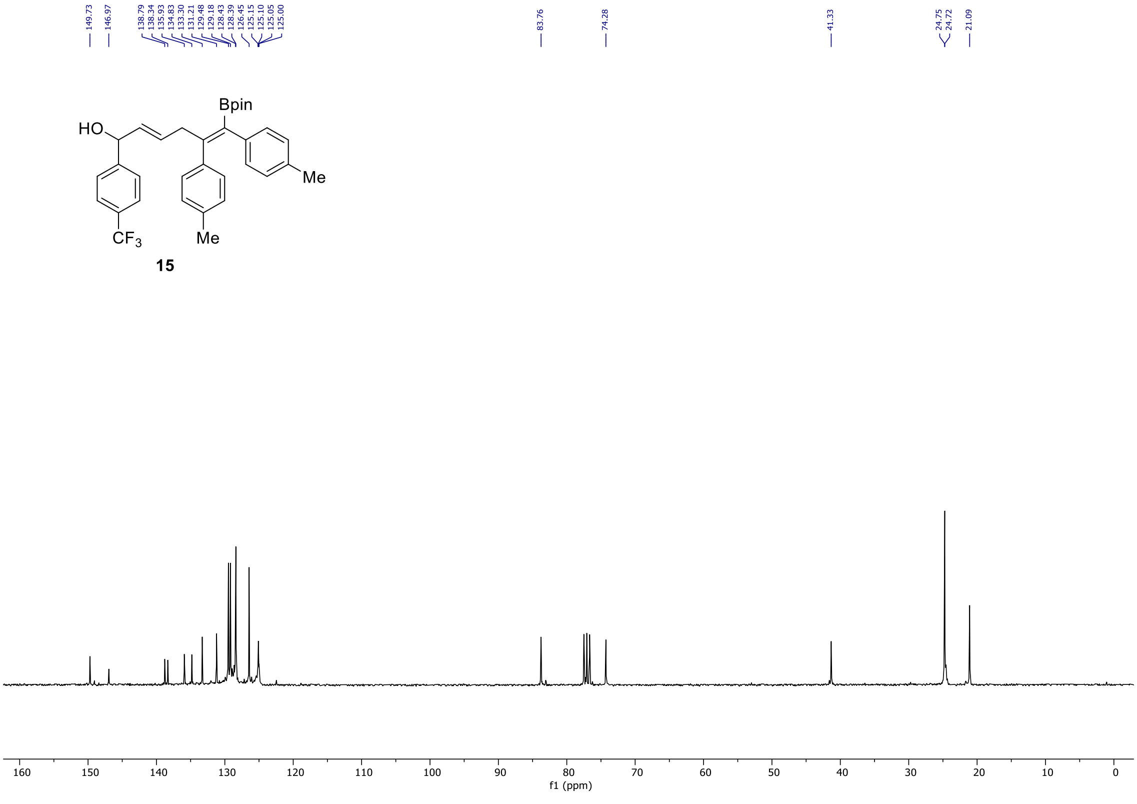

15

^{19}F NMR (282 MHz, $CDCl_3$)

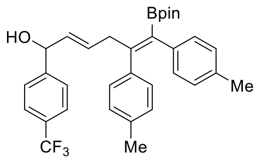

15

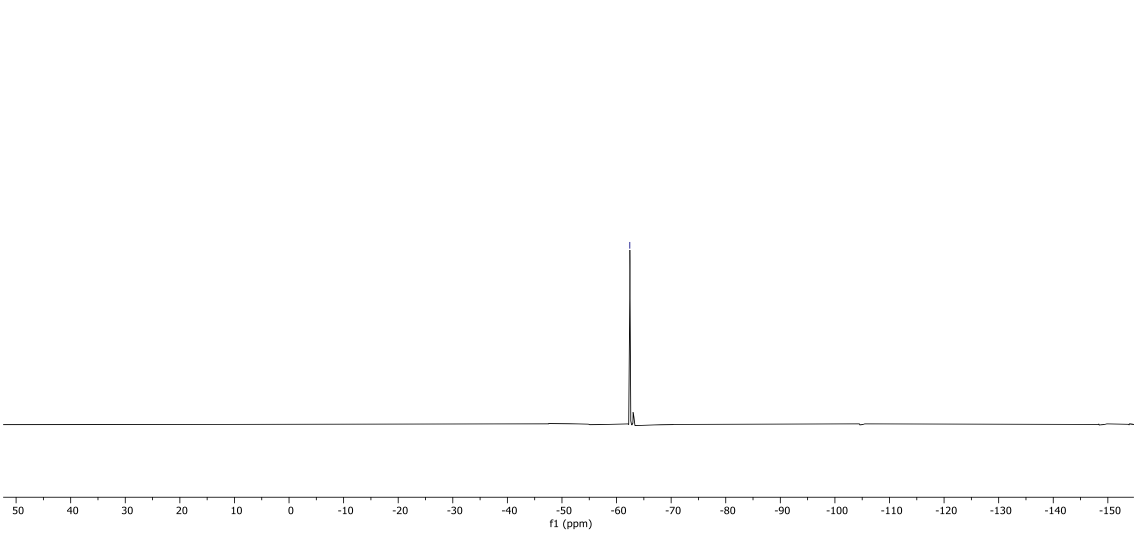

^{1}H NMR (500 MHz, $CDCl_3$)

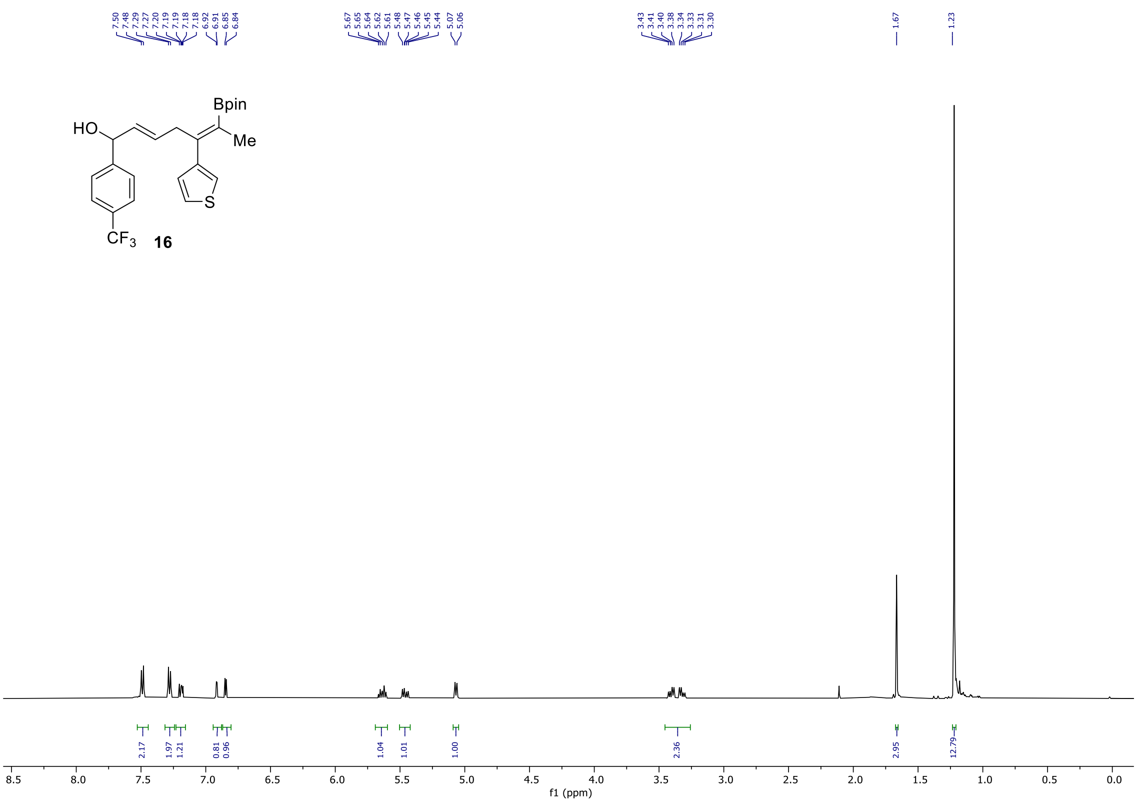

^{13}C NMR (75 MHz, $CDCl_3$)

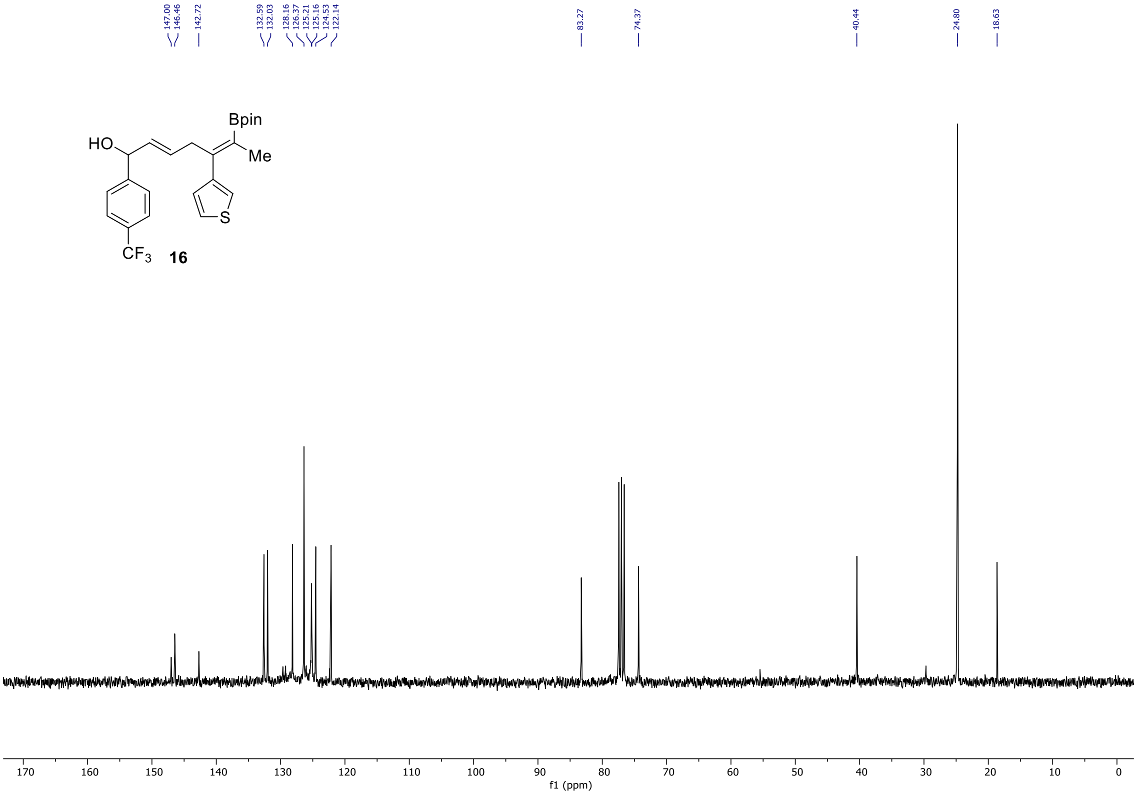

^{19}F NMR (282 MHz, $CDCl_3$)

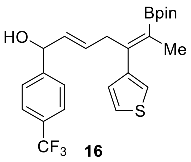

16

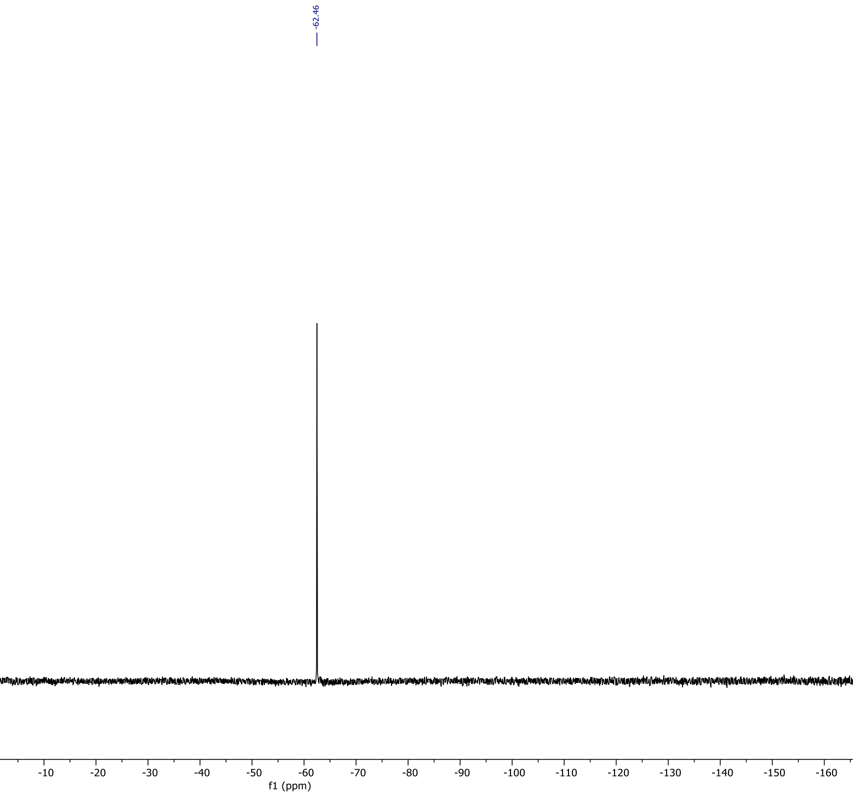

^{1}H NMR (500 MHz, $CDCl_3$)

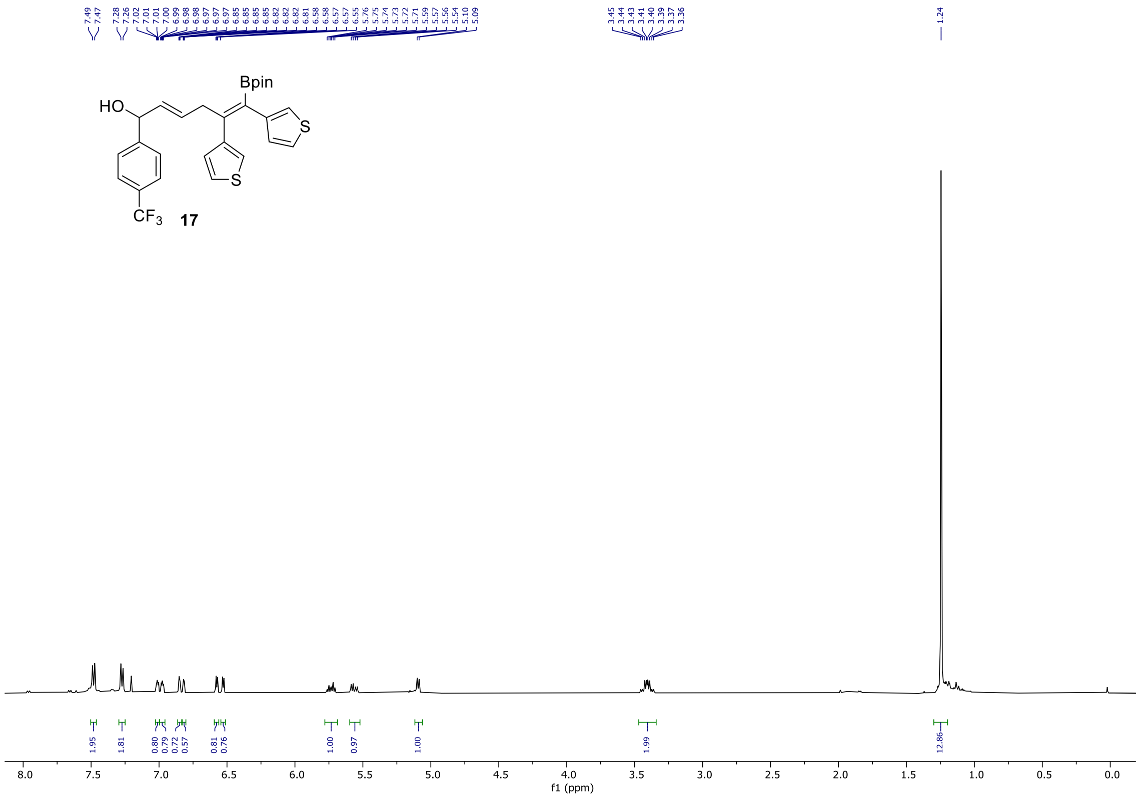

^{13}C NMR (75 MHz, $CDCl_3$)

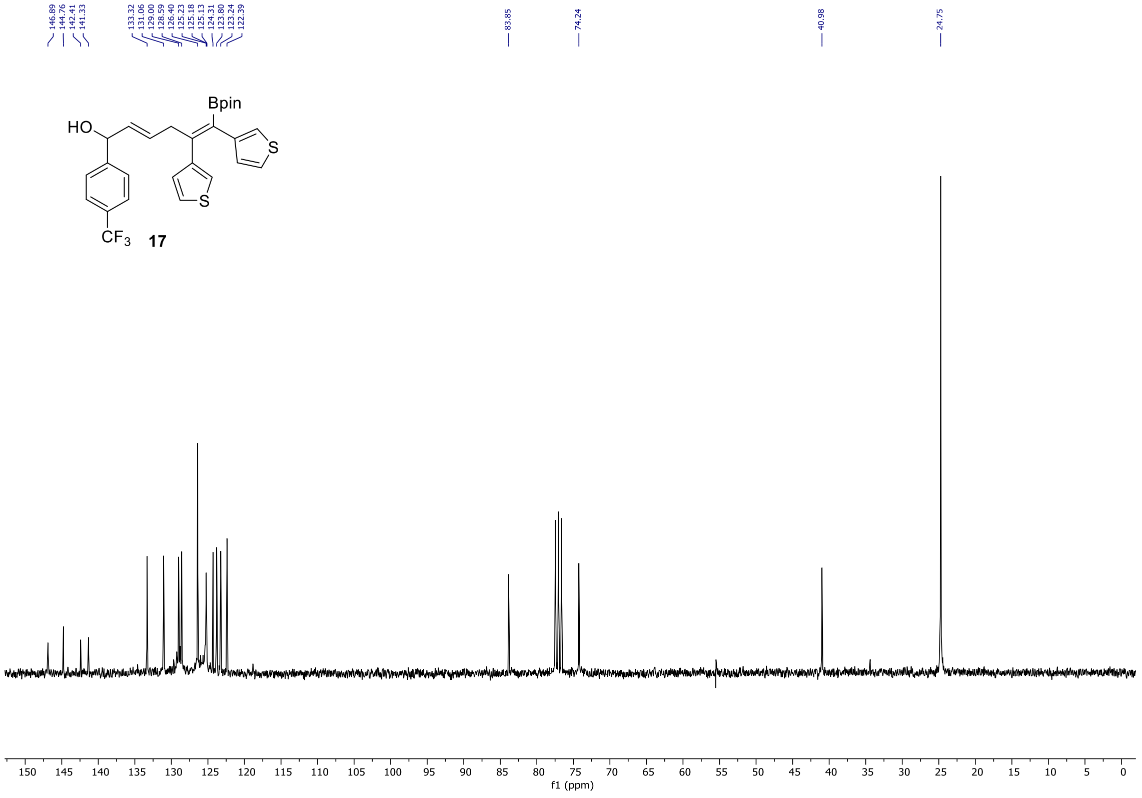

^{19}F NMR (282 MHz, $CDCl_3$)

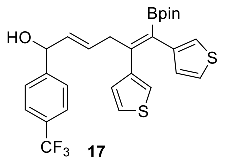

17

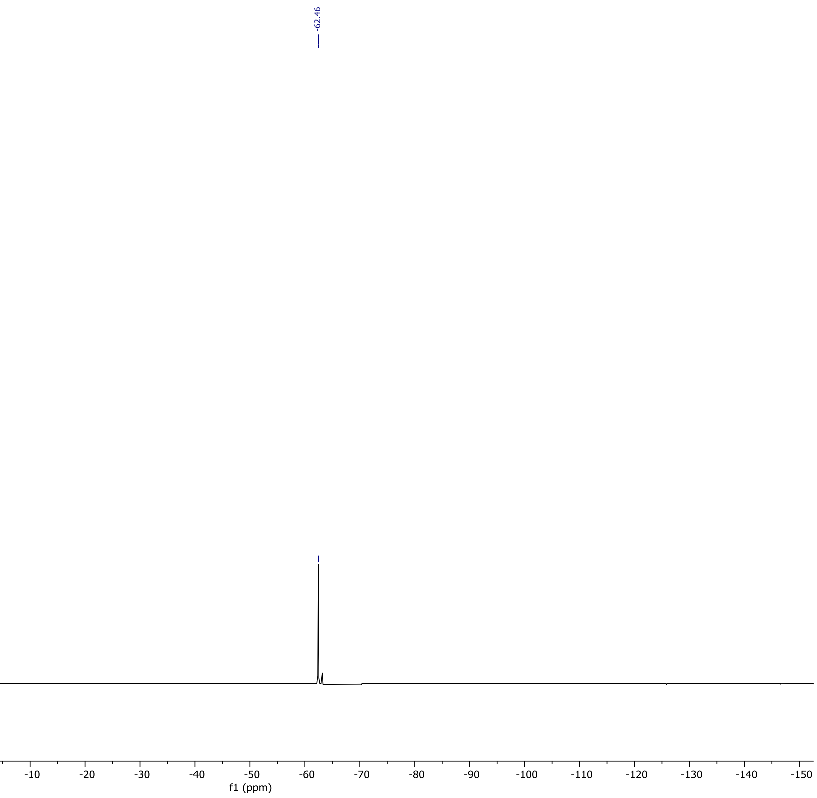

^{1}H NMR (300 MHz, $CDCl_3$)

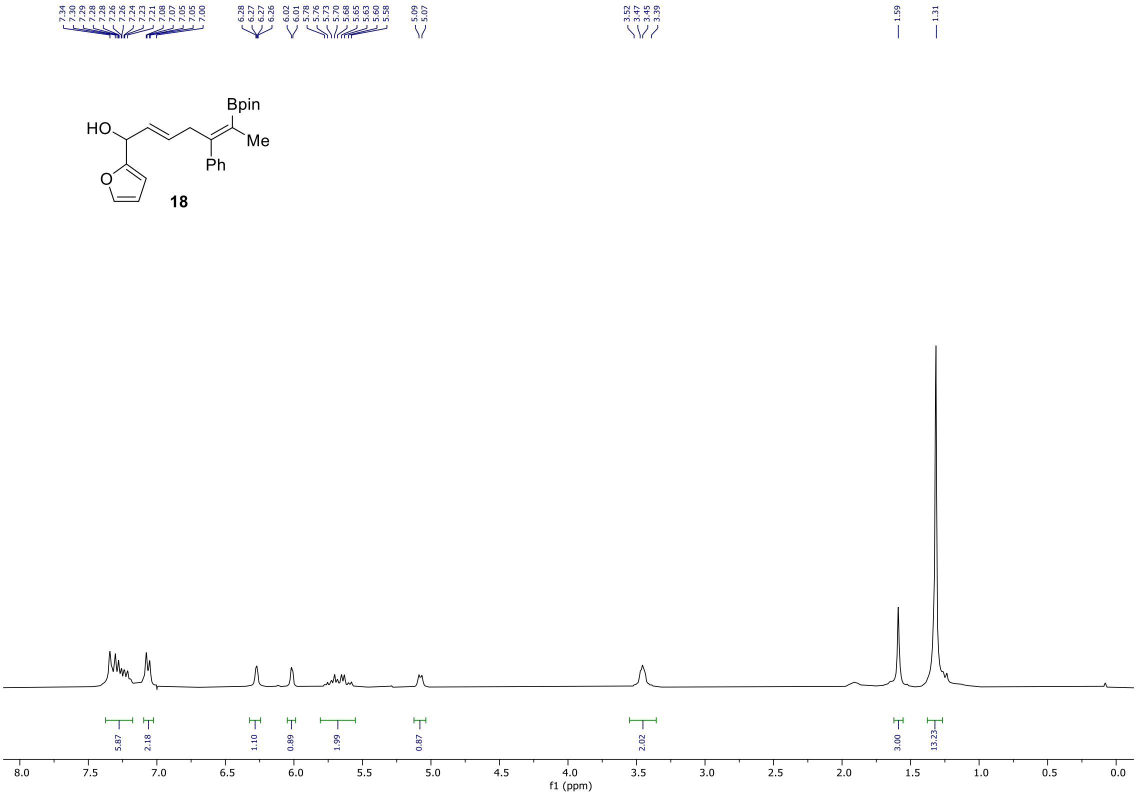

^{13}C NMR (75 MHz, $CDCl_3$)

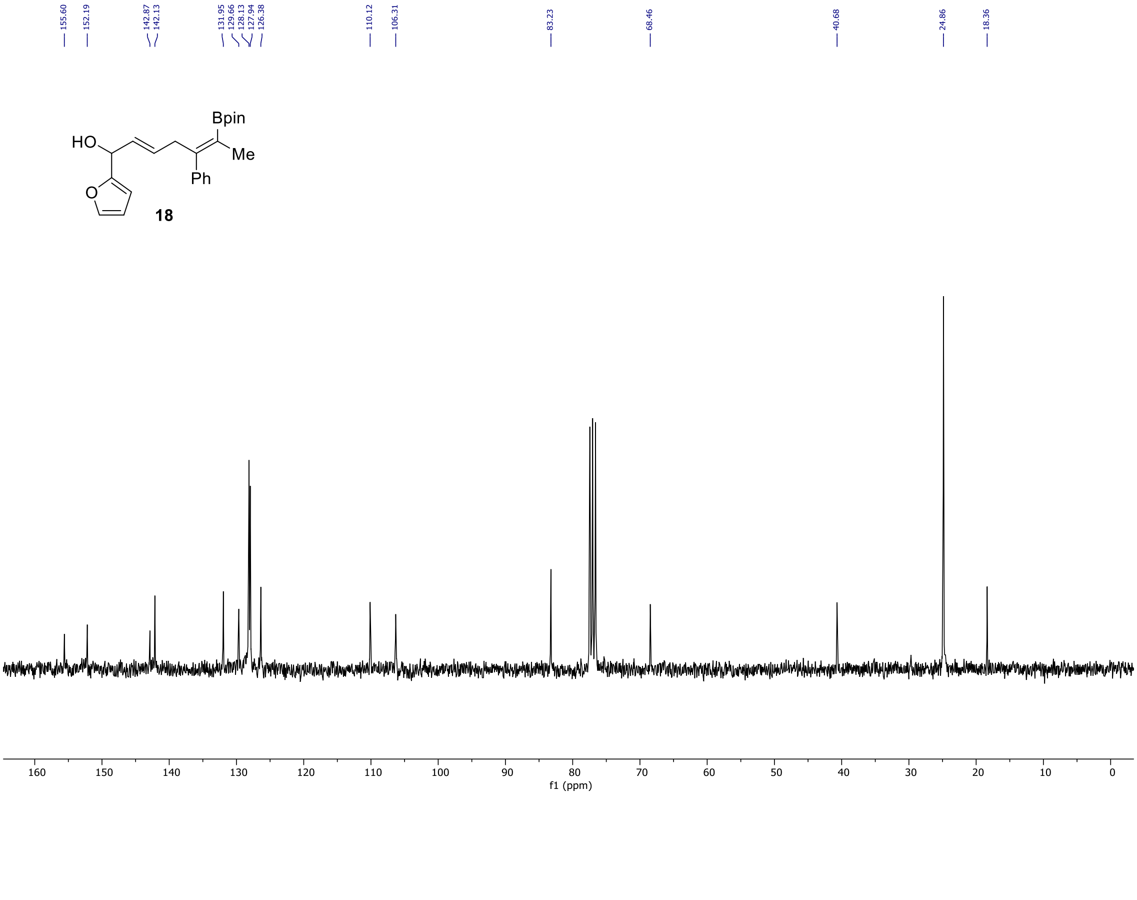

^{1}H NMR (500 MHz, CDCl$_3$)

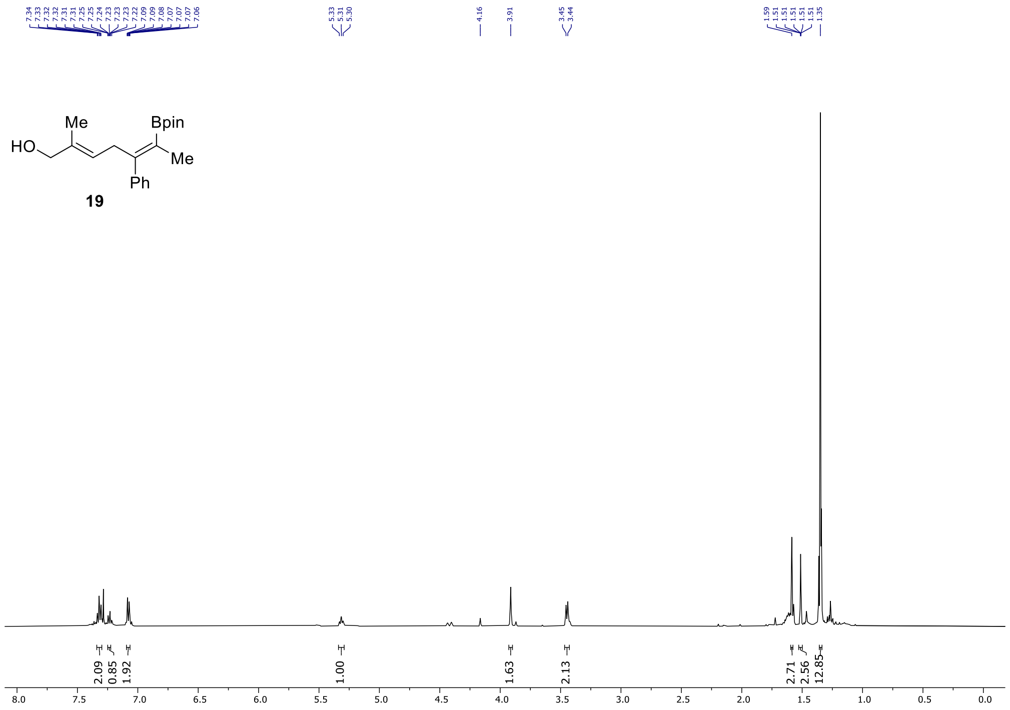

^{13}C NMR (126 MHz, CDCl$_3$)

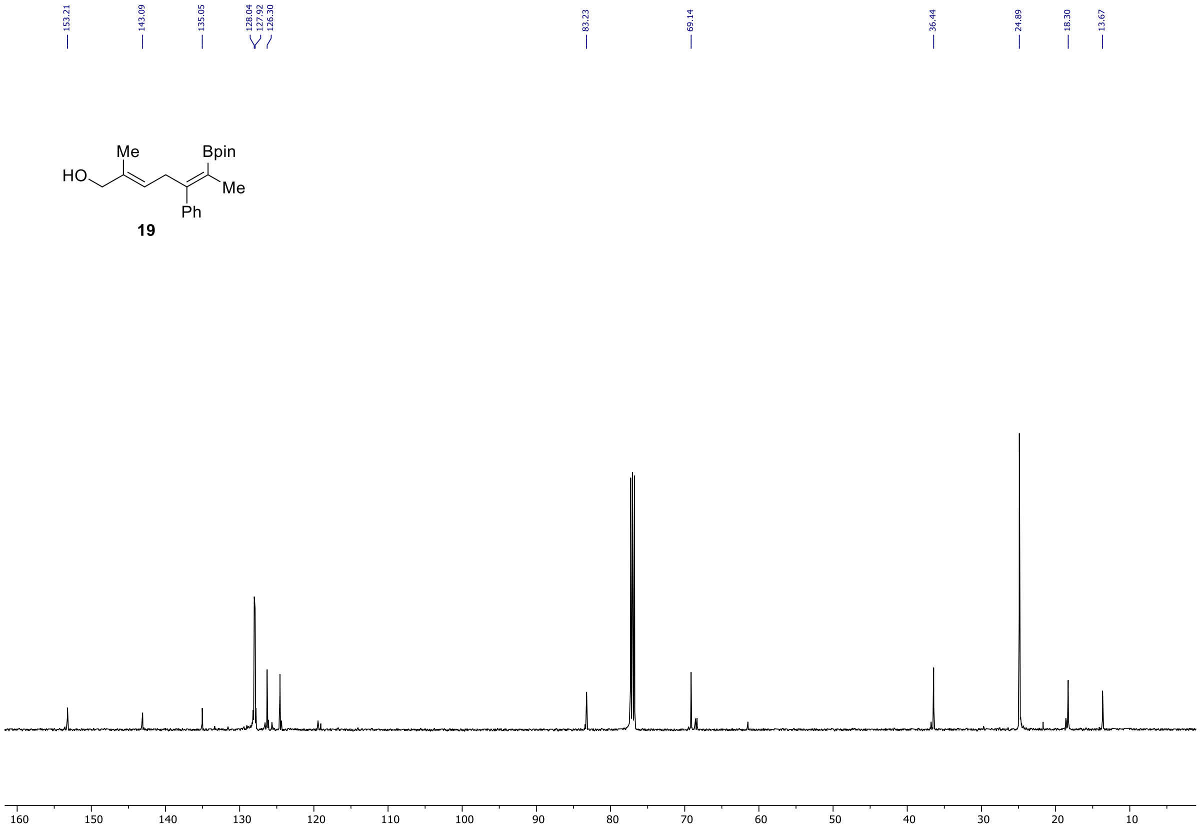

^{1}H NMR (500 MHz, $CDCl_3$)

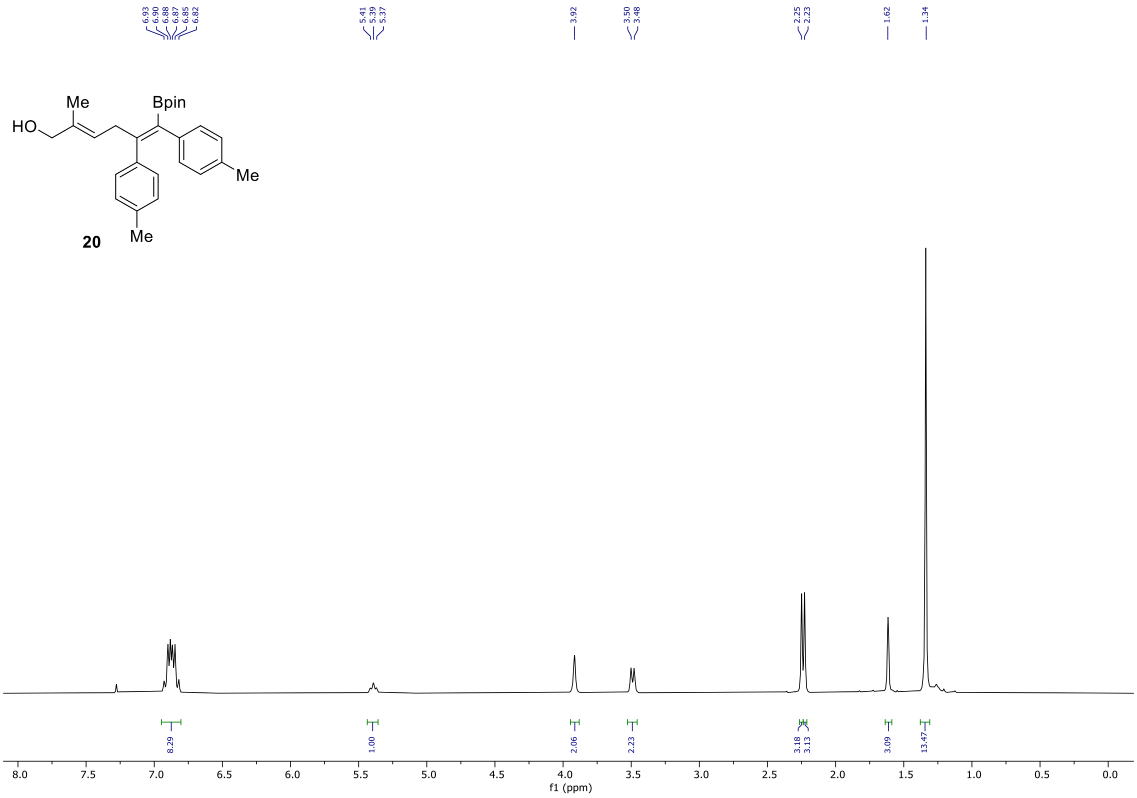

^{13}C NMR (162 MHz, $CDCl_3$)

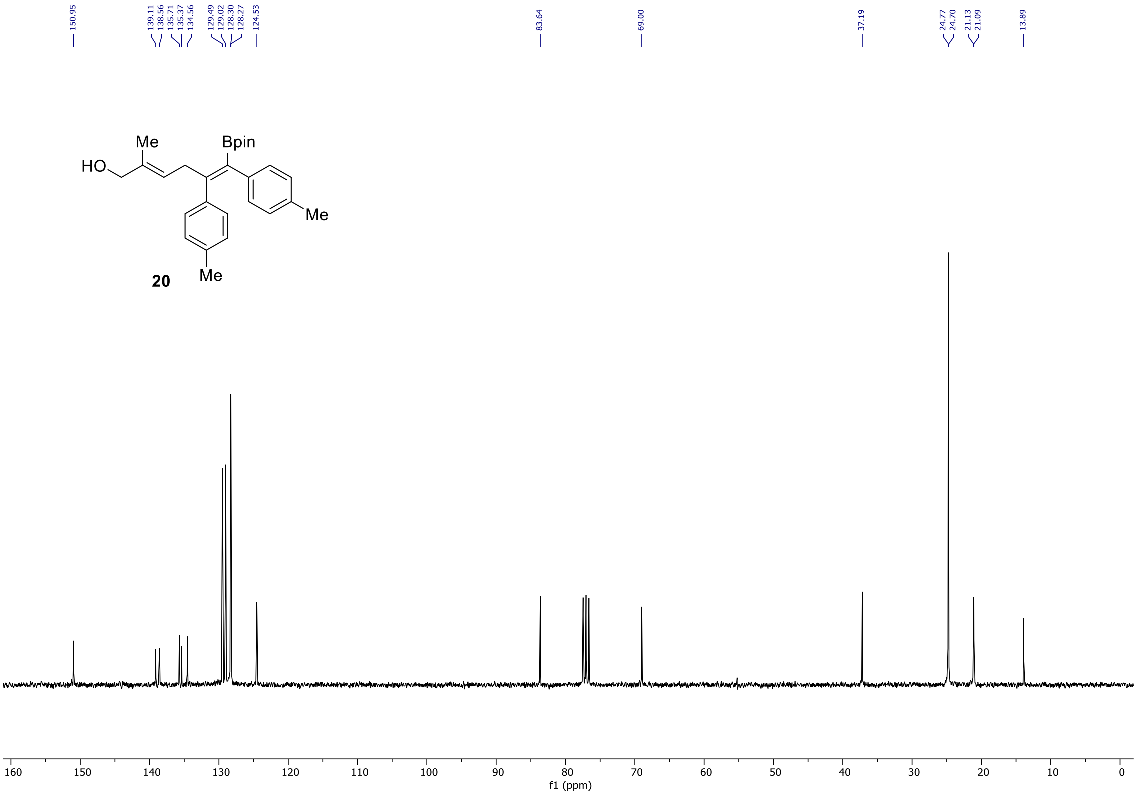

^{1}H NMR (500 MHz, $CDCl_3$)

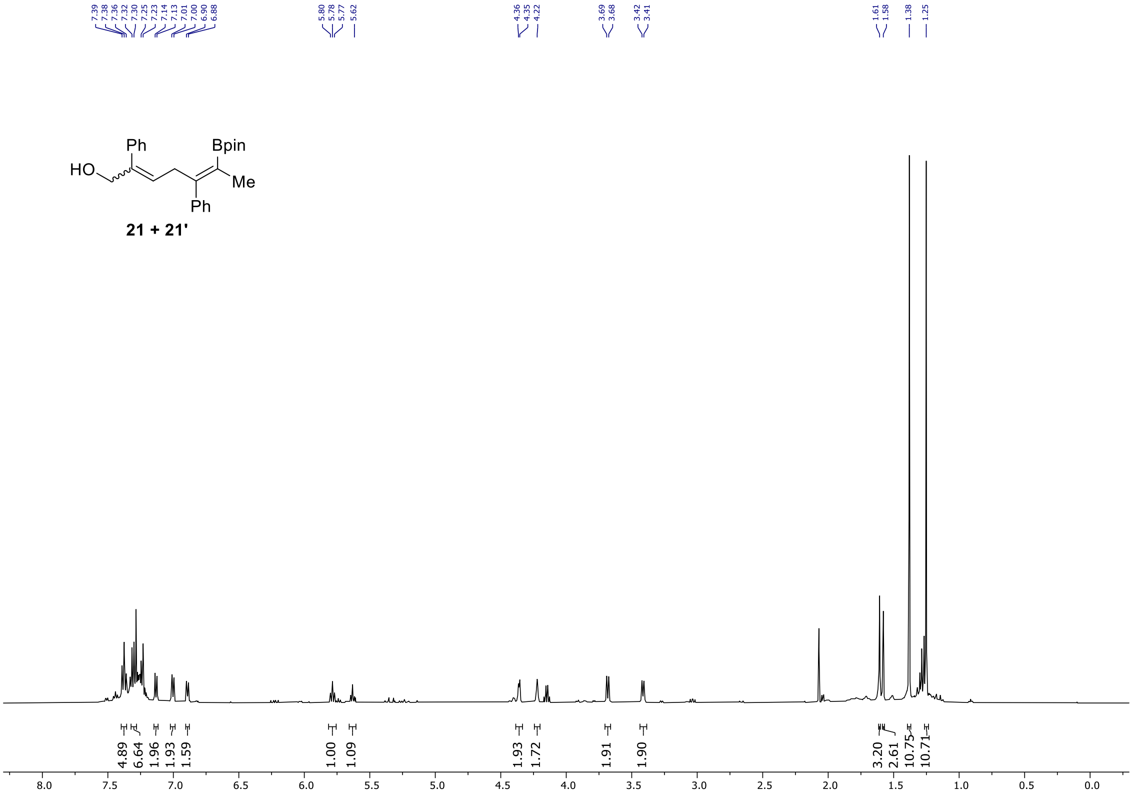

^{13}C NMR (126 MHz, $CDCl_3$)

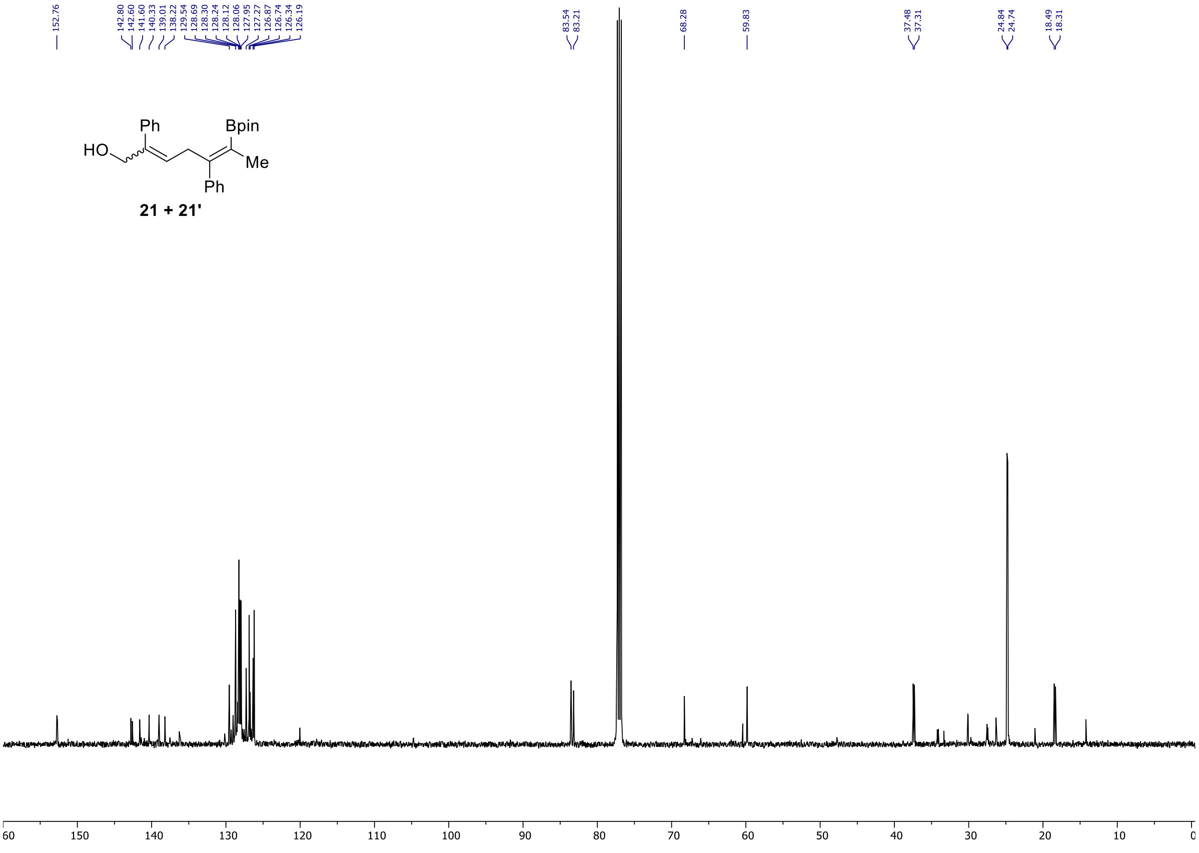

COSY

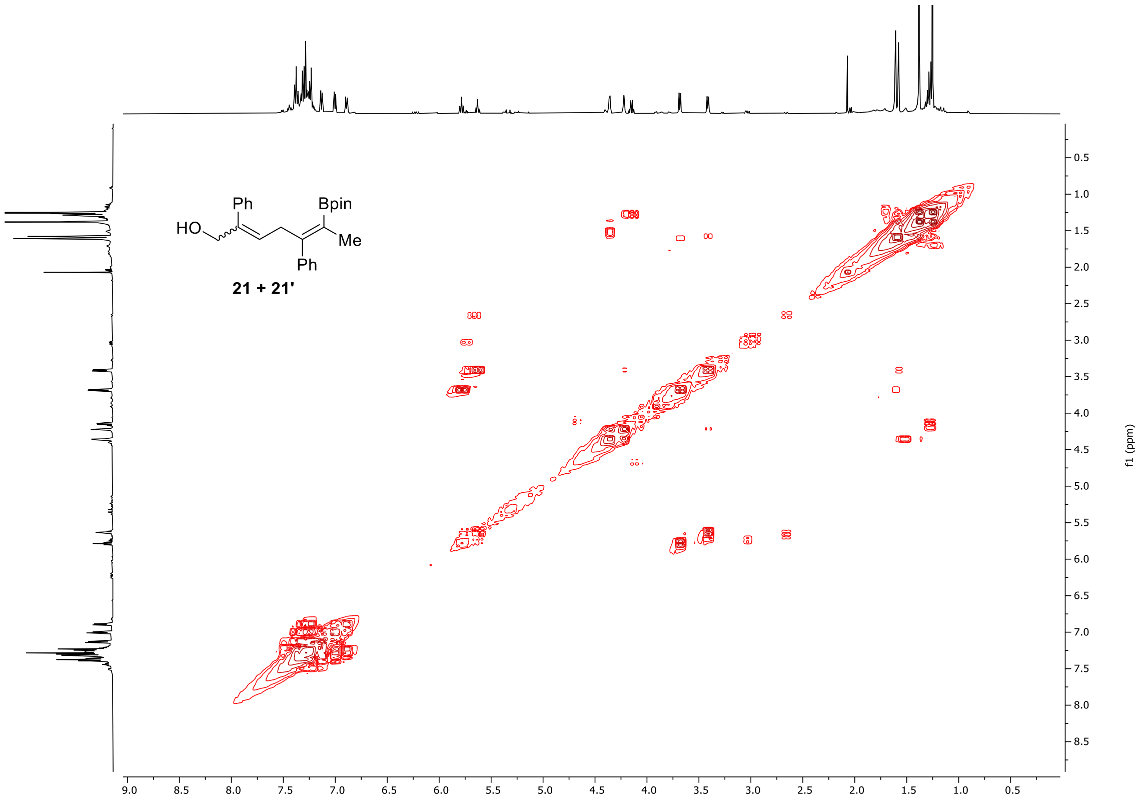

Ph
Bpin
HO
Me
Ph
21 + 21'
f1 (ppm)
9.0
8.5
8.0
7.5
7.0
6.5
6.0
5.5
5.0
4.5
4.0
3.5
3.0
2.5
2.0
1.5
1.0
0.5

^{1}H NMR (500 MHz, $CDCl_3$)

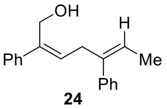

24

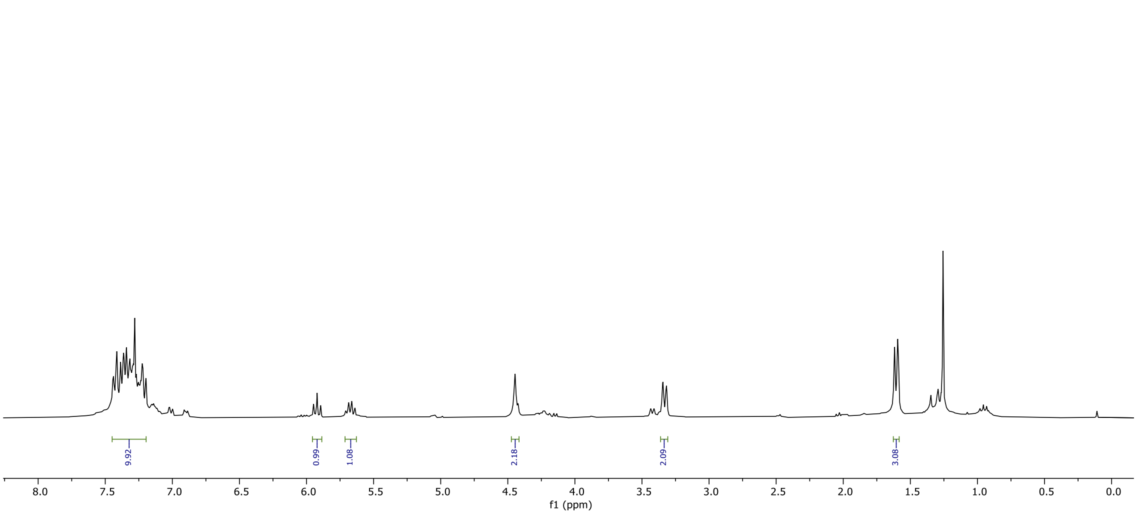

^{13}C NMR (126 MHz, $CDCl_3$)

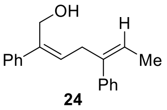

24

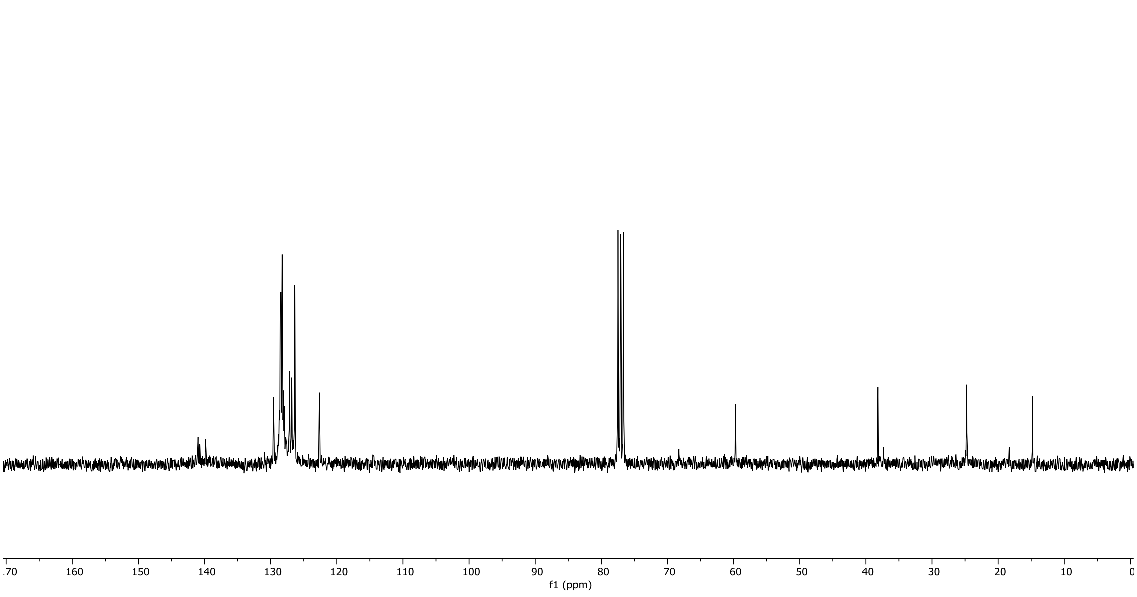

^{1}H NMR (300 MHz, $CDCl_3$)

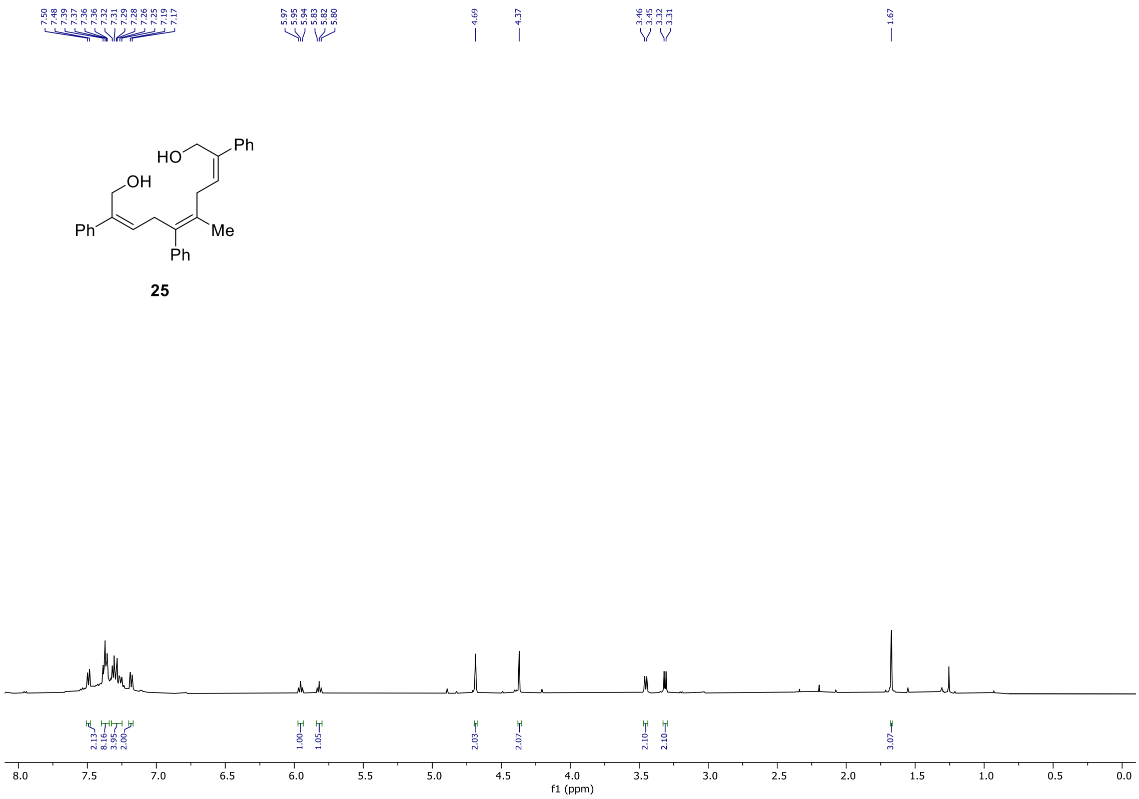

^{13}C NMR (126 MHz, $CDCl_3$)

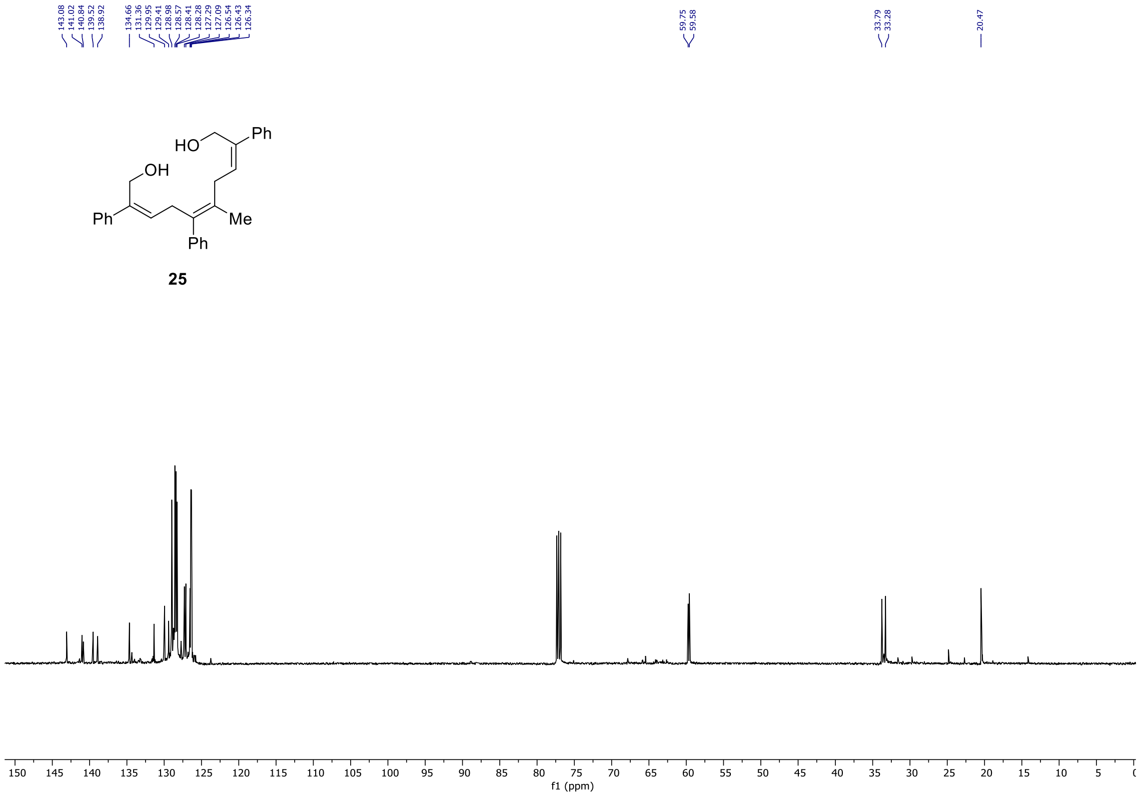

HSQC

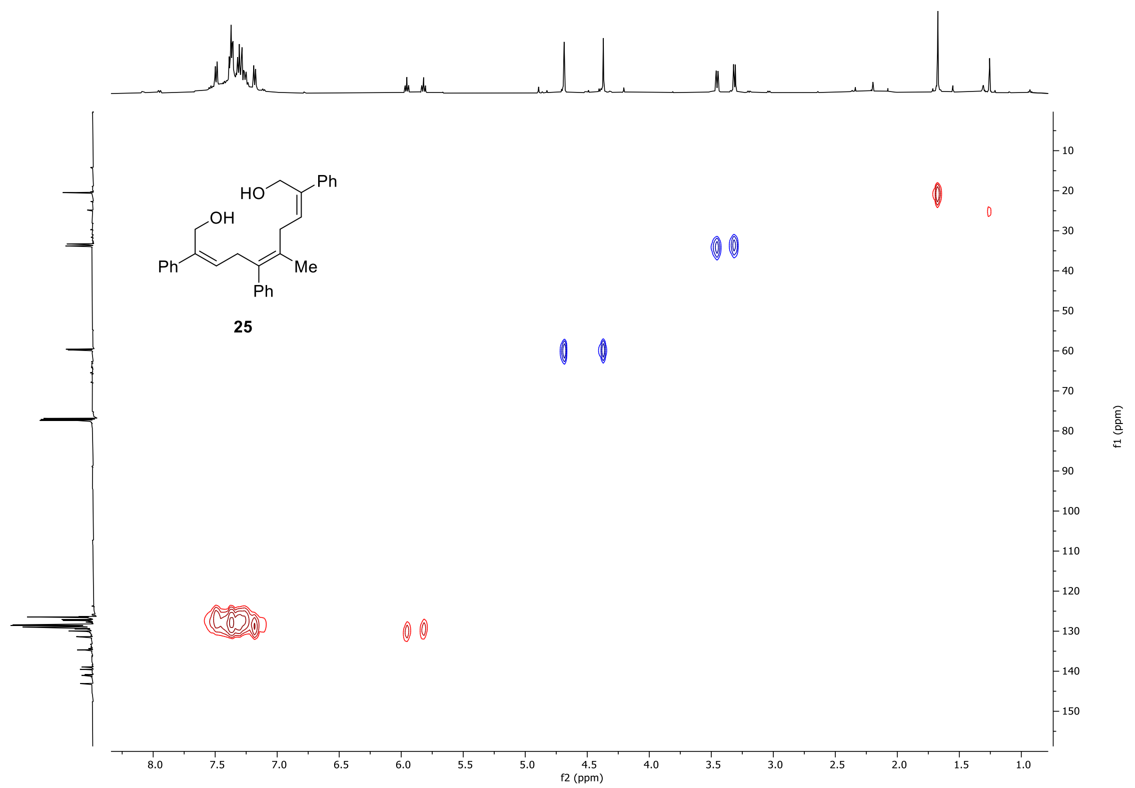

HMBC

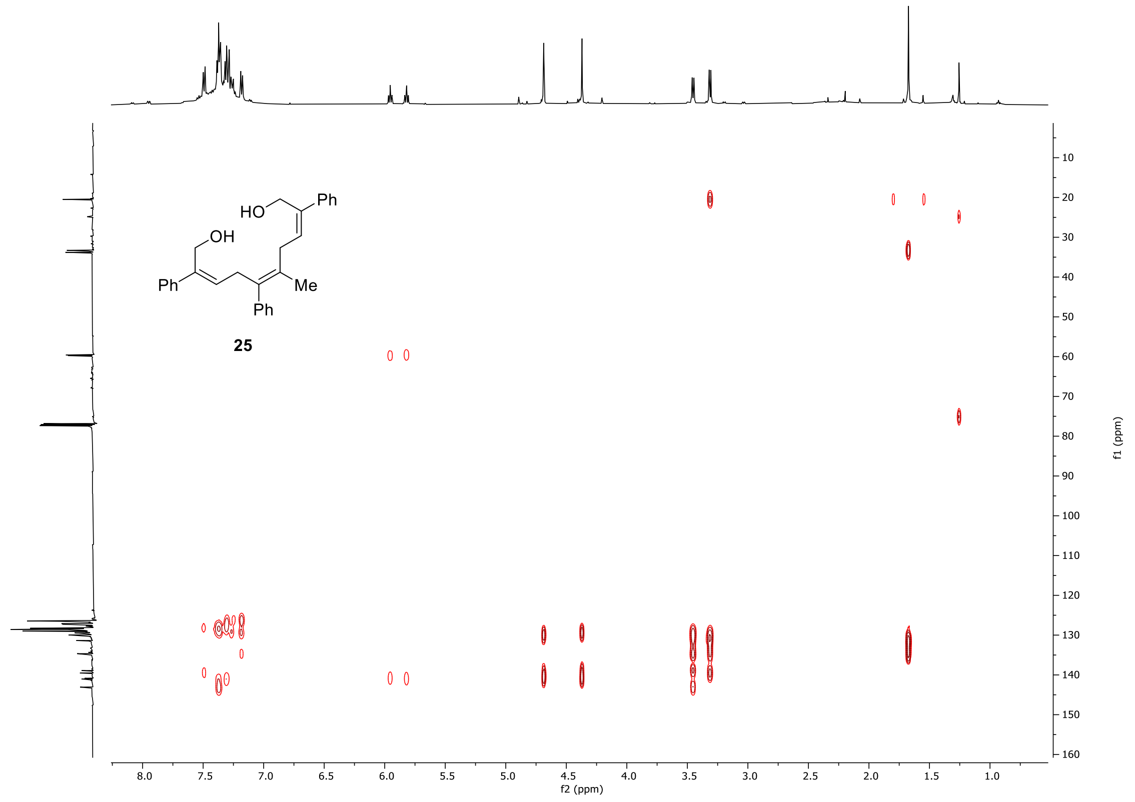

^{1}H NMR (300 MHz, $CDCl_3$)

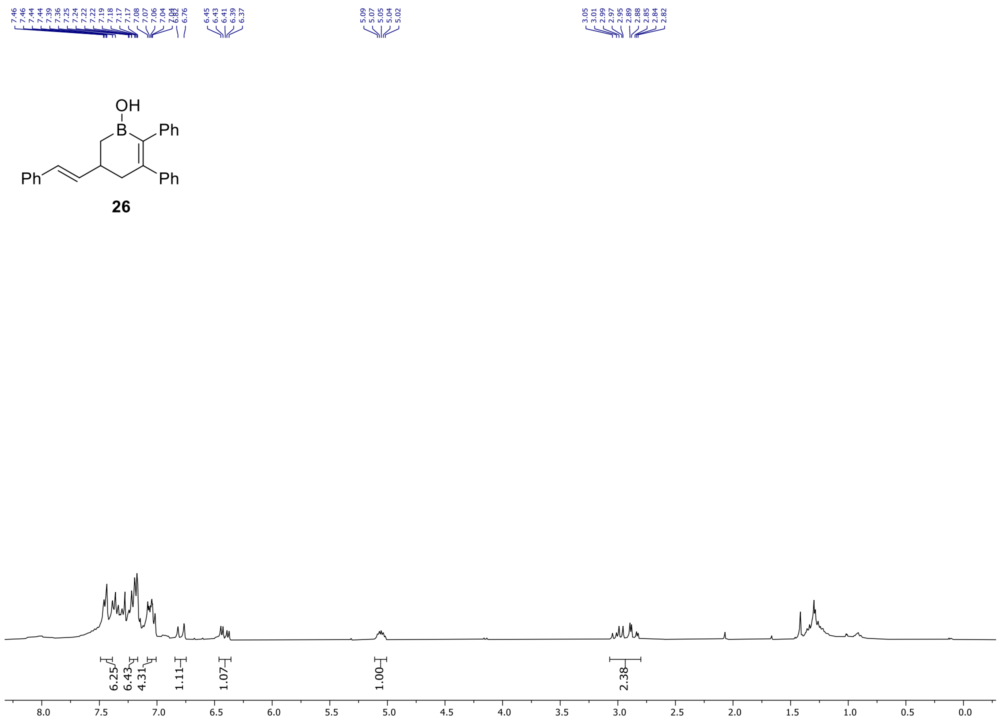

^{13}C NMR (75 MHz, $CDCl_3$)

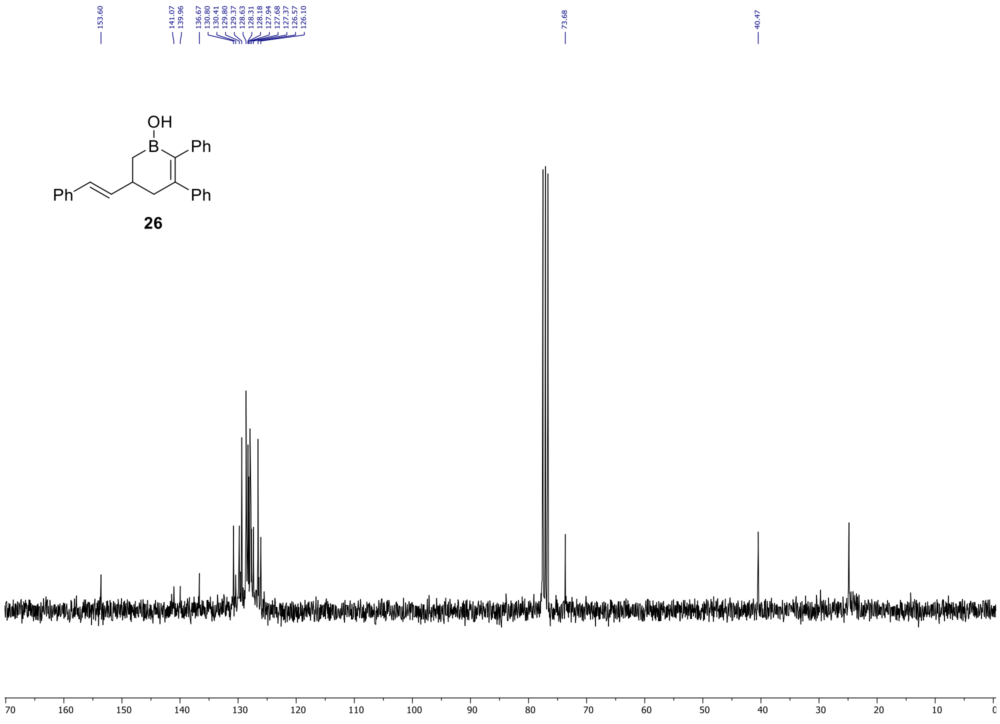

^{1}H NMR (500 MHz, $CDCl_3$)

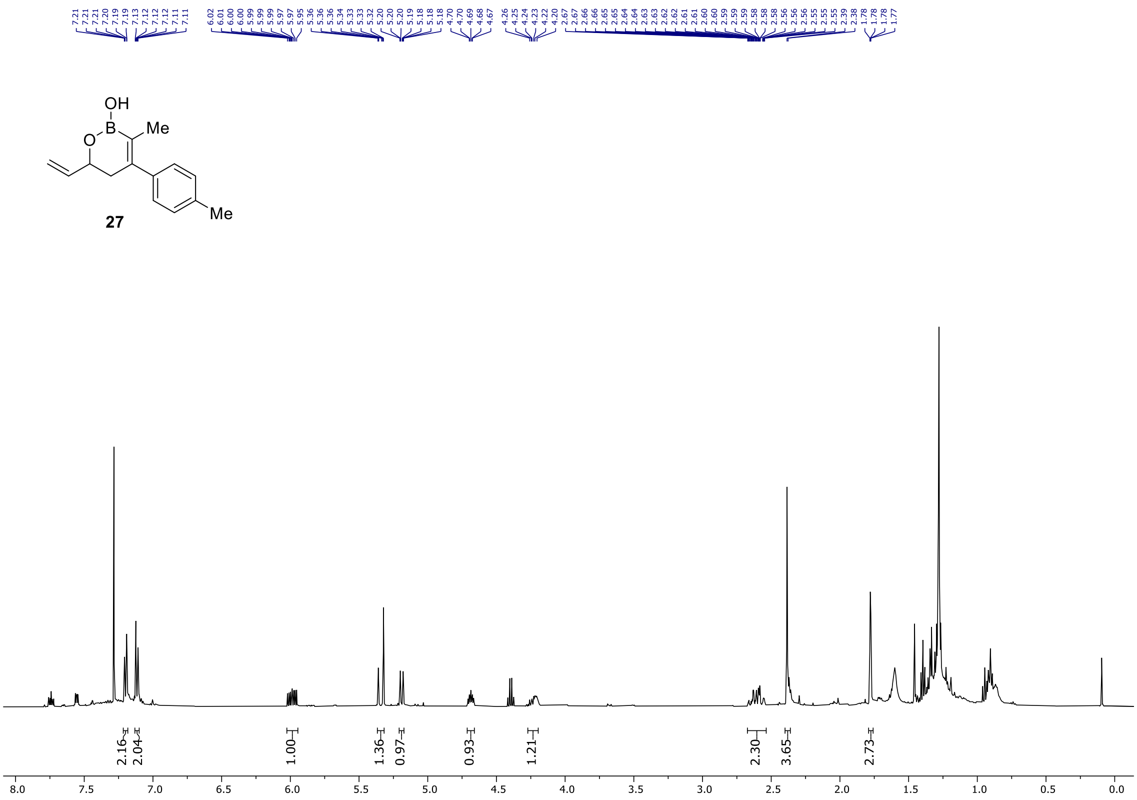

27

^{13}C NMR (126 MHz, $CDCl_3$)

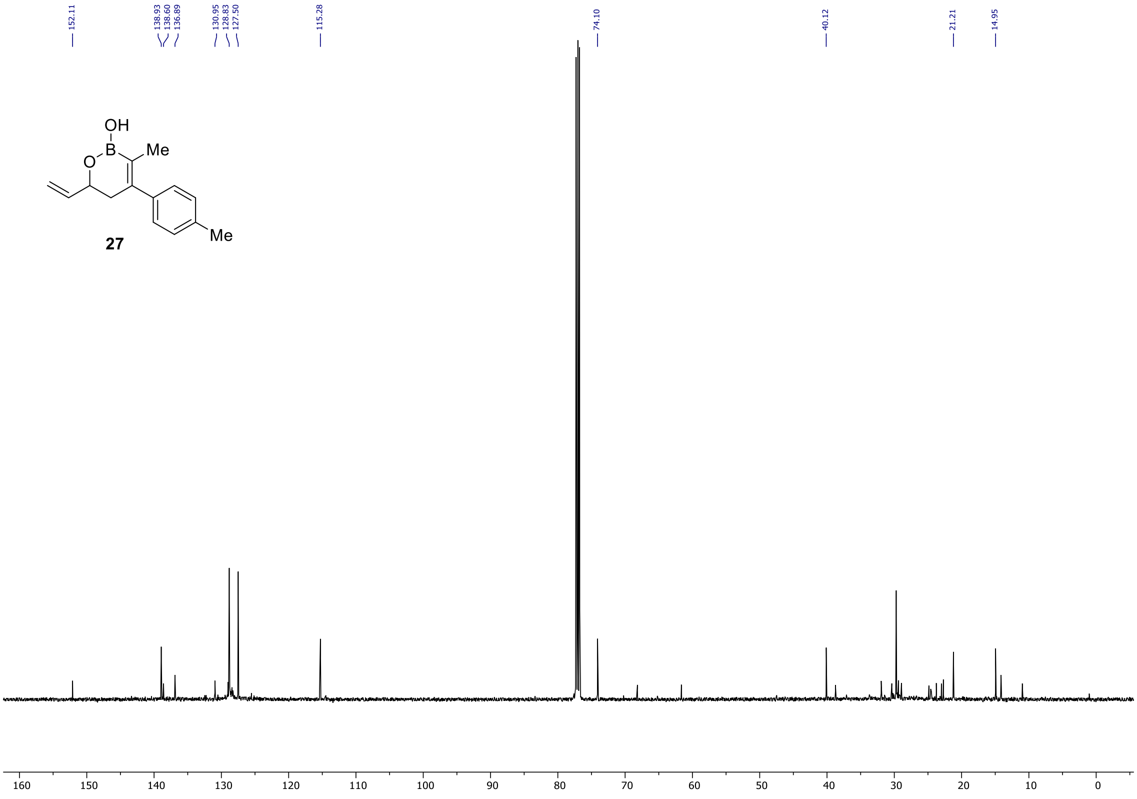

27

^{1}H NMR (500 MHz, $CDCl_3$)

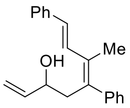

28

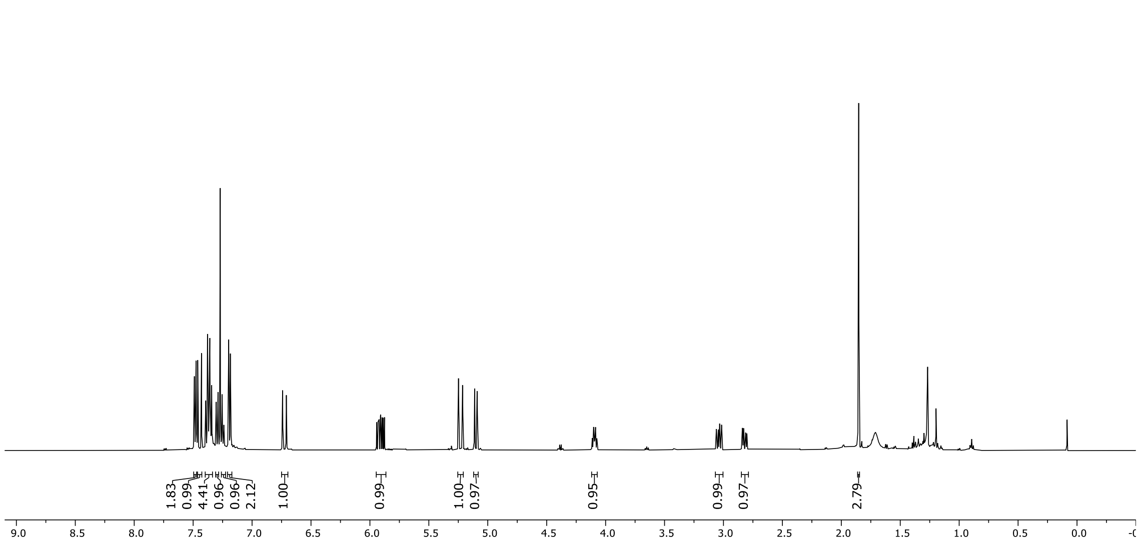

^{13}C-NMR (126 MHz, $CDCl_3$)

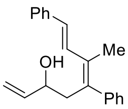

28

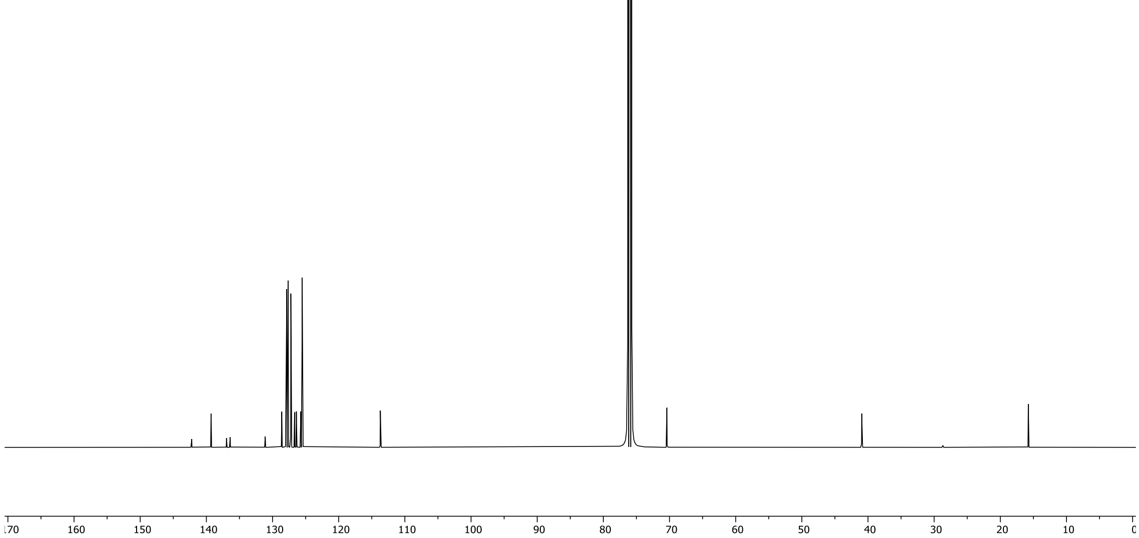

^{1}H NMR (300 MHz, $CDCl_3$)

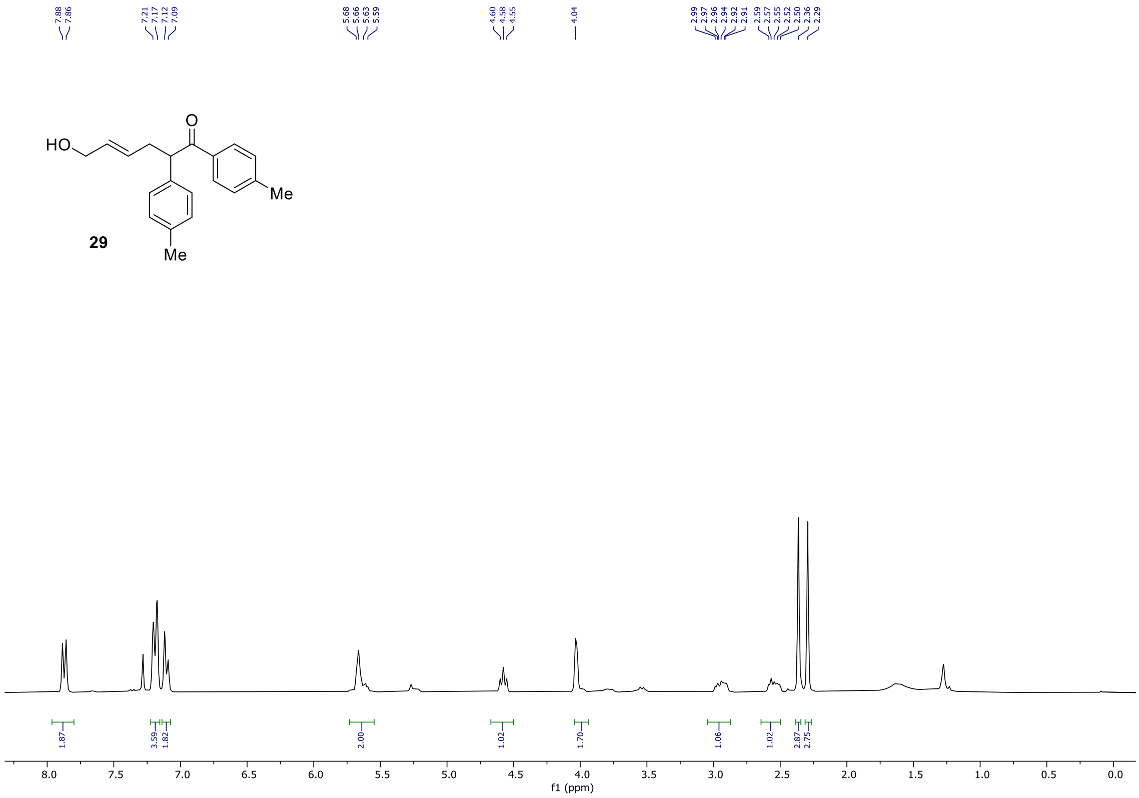

^{13}C NMR (75 MHz, $CDCl_3$)

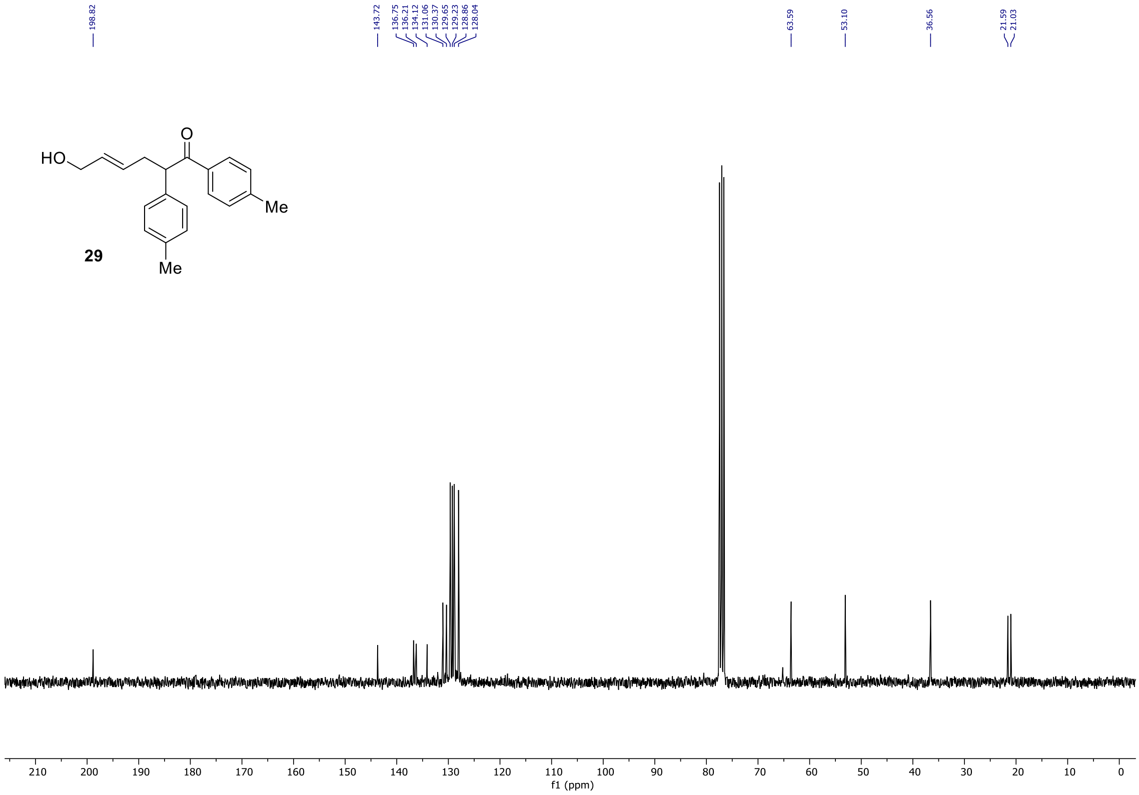

^{1}H NMR (300 MHz, $CDCl_3$)

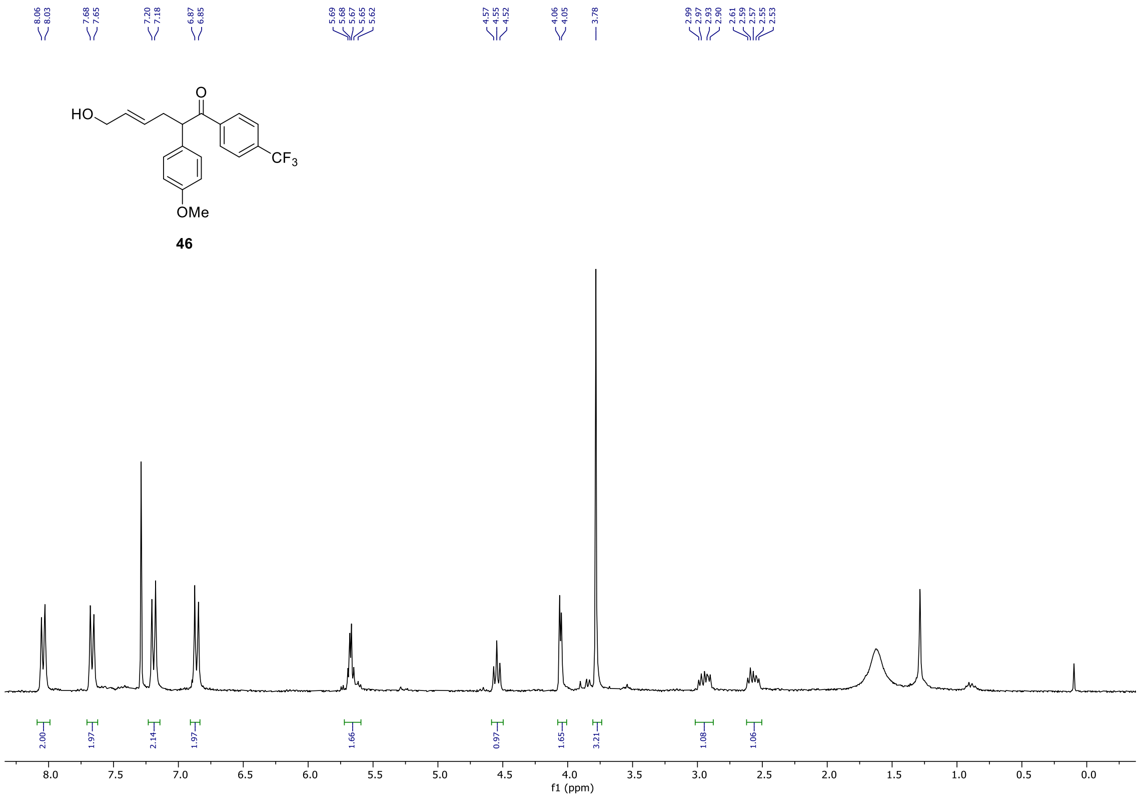

^{13}C NMR (75 MHz, $CDCl_3$)

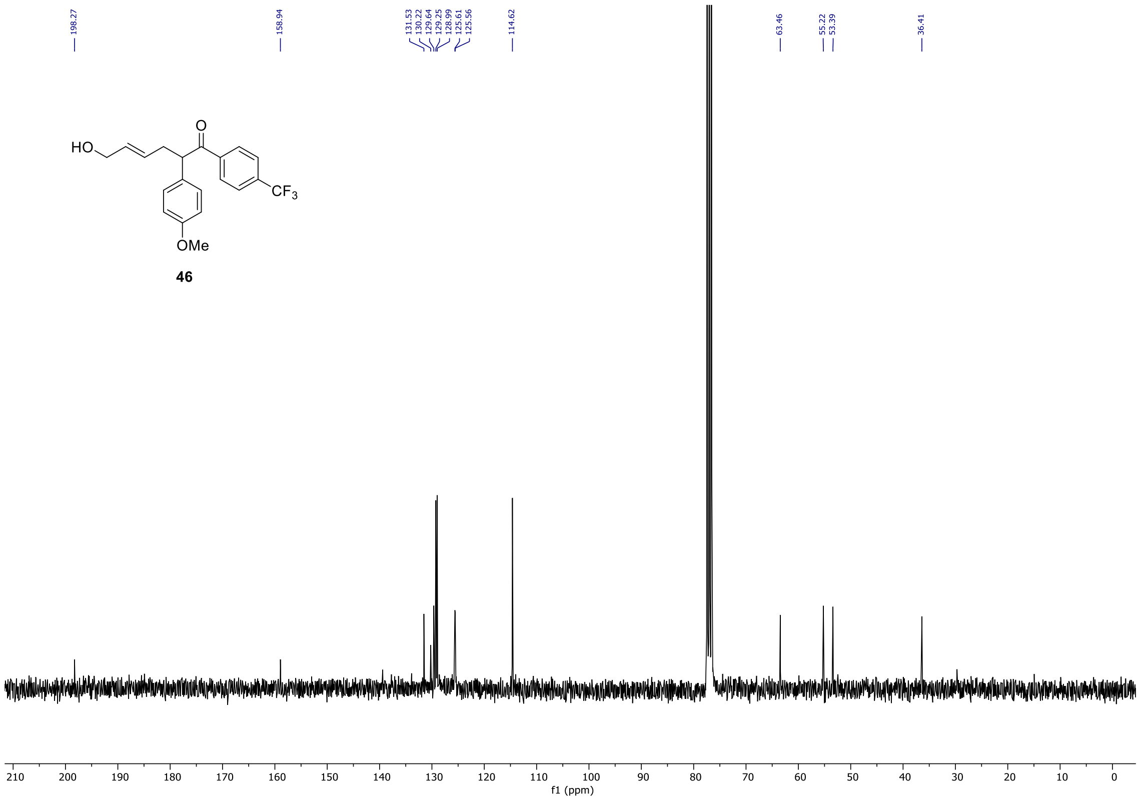

9. Stereochemistry determination

- **Compound 3**

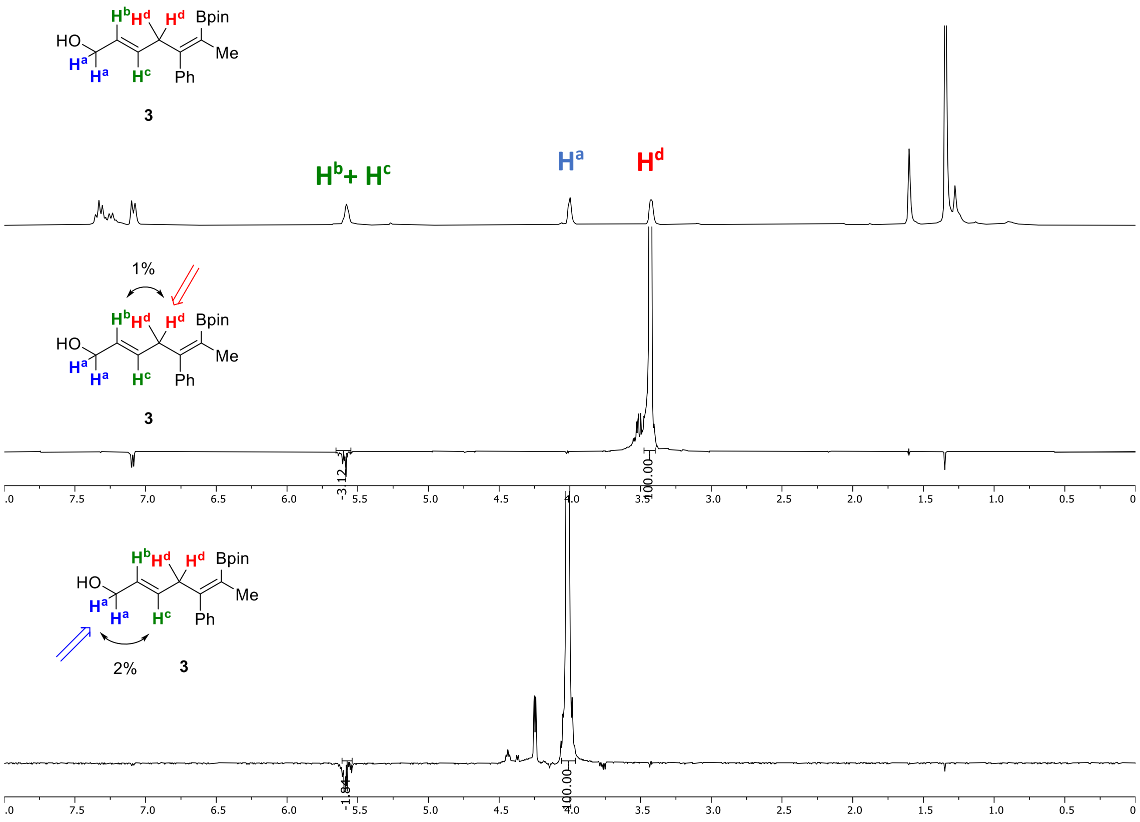

The irradiation at CH_2 signal (H^d) induces a NOE response on the CH corresponding to (H^b and H^c) and not on the CH_2 corresponding to (H^a).

Similarly, irradiation at CH_2 signal (H^a) induces a NOE response on the CH corresponding to (H^b and H^c) and not on the CH_2 corresponding to (H^d).

- **Compound 19**

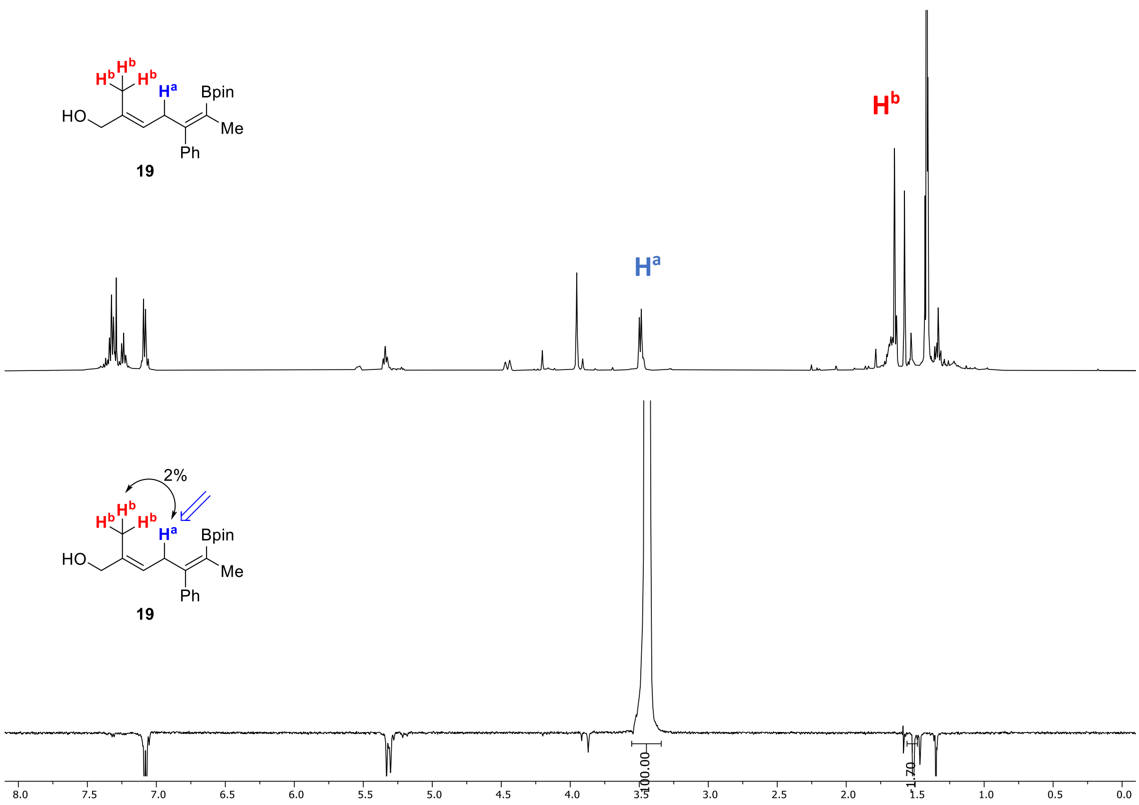

The irradiation at CH_2 signal (H^a) induces a NOE response on the CH_3 corresponding to (H^b).

- **Compounds 21 and 21'**

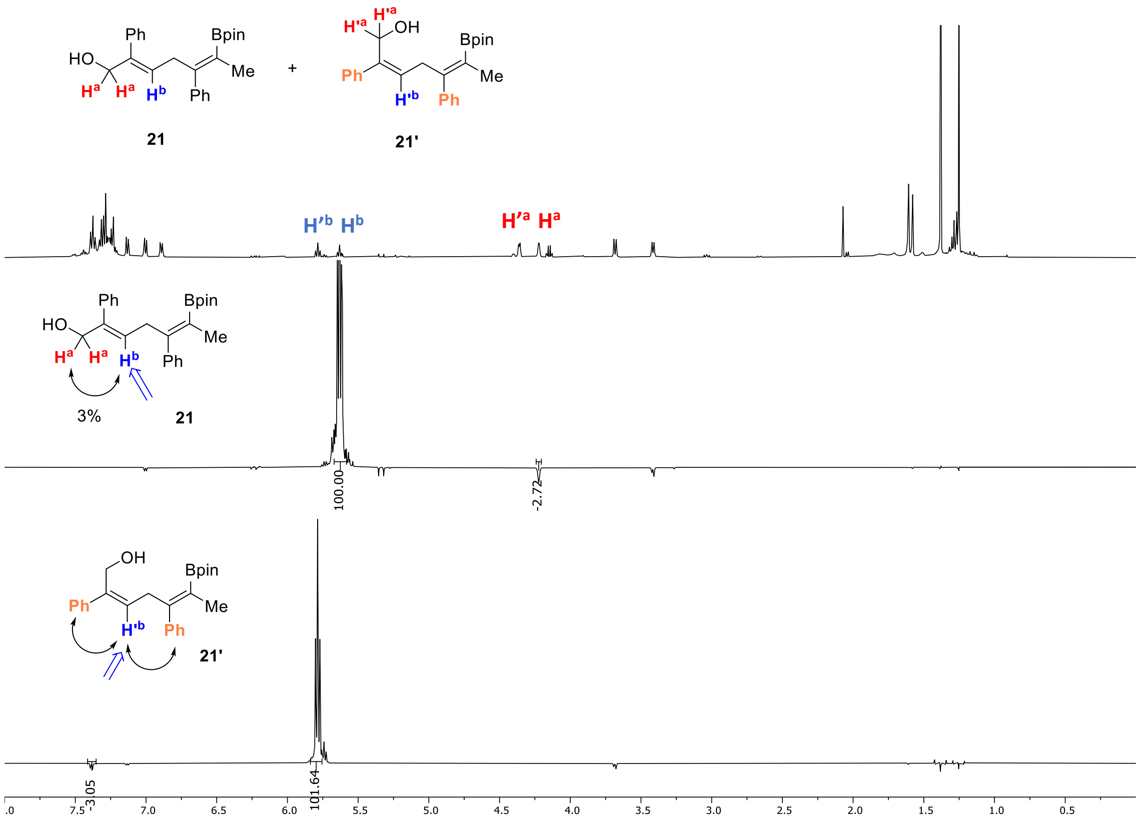

The irradiation at CH signal (**H**^{b}) induces a NOE response on the CH_2 corresponding to (**H**^{a}).

The irradiation at CH signal (**H'**^{b}) induces a NOE response on the CH corresponding to phenyl group (**H**^{Ph}).

- **Compound 24**

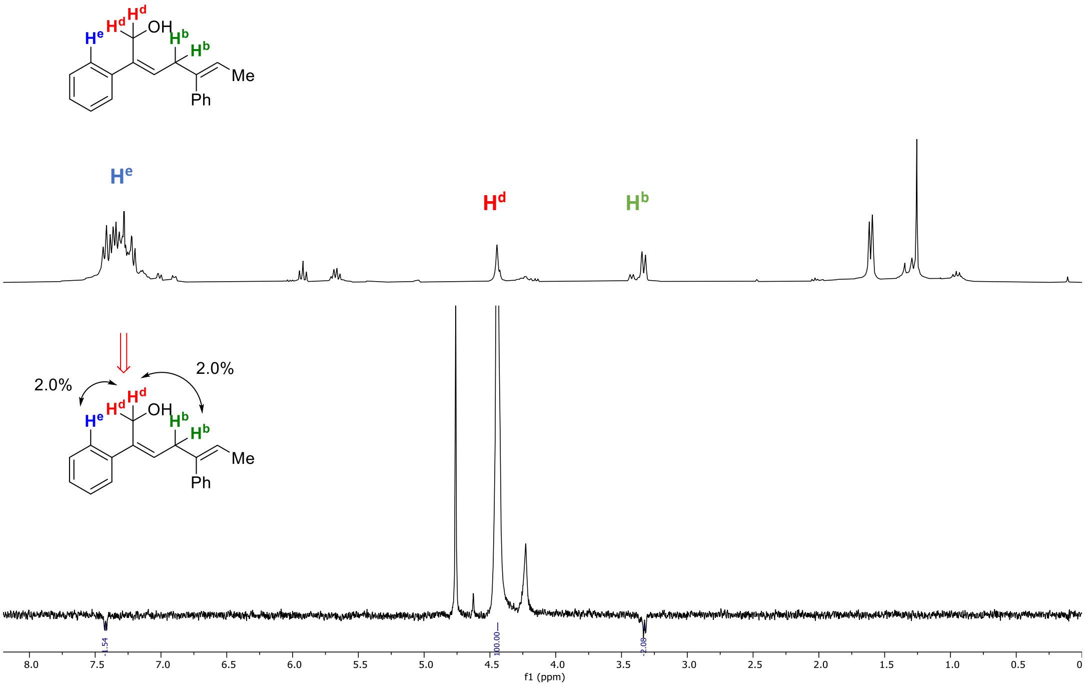

The irradiation at CH_2 signal (**H**[d]) induces a NOE response on the CH_2 corresponding to (**H**[b]) and on the CH corresponding to (**H**[e]).

- **Compound 25**

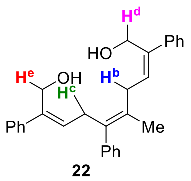

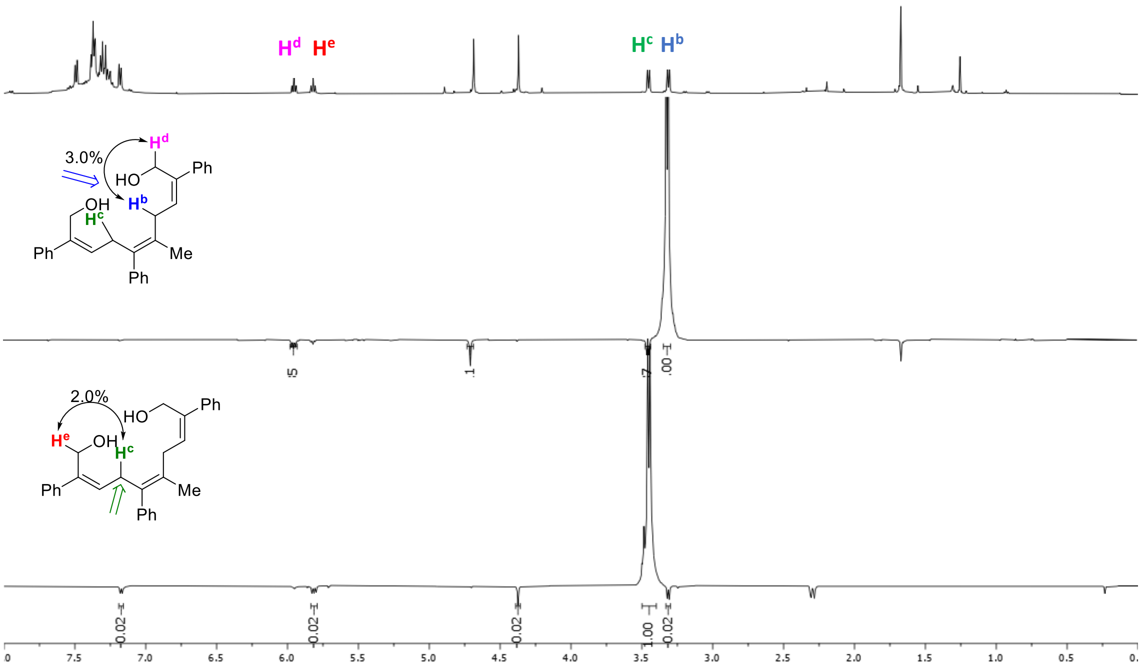

The irradiation at CH_2 signal (H^b) induces a NOE response on the CH_2 corresponding to (H^d and $H^{d'}$) and on the CH_2 corresponding to (H^c).

The irradiation at CH_2 signal (H^c) induces a NOE response on the CH_2 corresponding to (H^e).

10. Regiochemistry determination for compound 46 (and 6 and 6')

The structure of compounds **6** and **6'** (relative position of the two different aryl groups) could be determined by carrying out the following experiment.

After oxidation of a 2:1 mixture of compounds **6** and **6'** following the procedure described in section 6.3, the formation of a 2:1 mixture of ketone **46** and **46'** was observed by ^{19}F NMR. After column chromatography, it was possible to isolate the pure major product **46**, which structure was elucidated by bidimensional NMR analysis (see below).

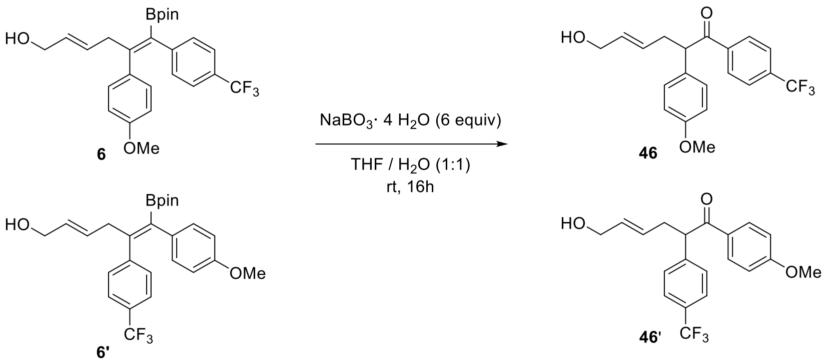

^{19}F NMR crude product after oxidation

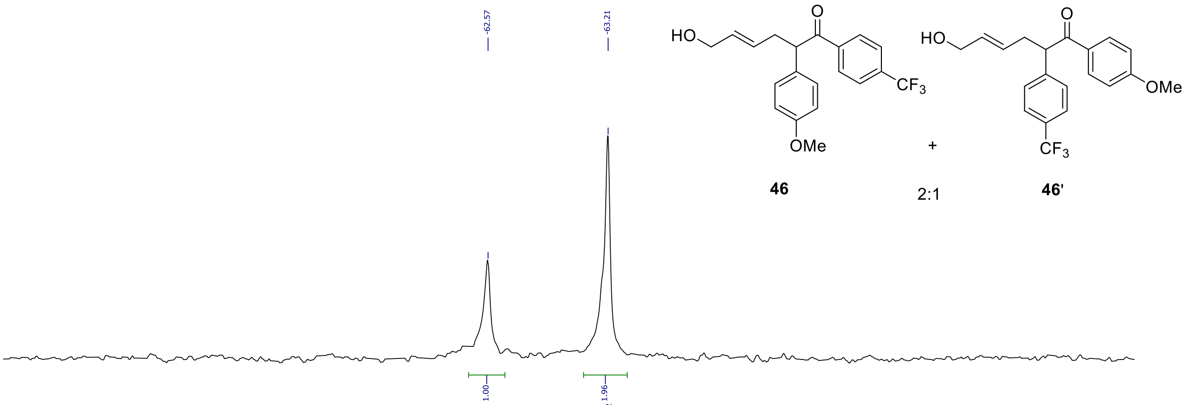

19F NMR isolated product

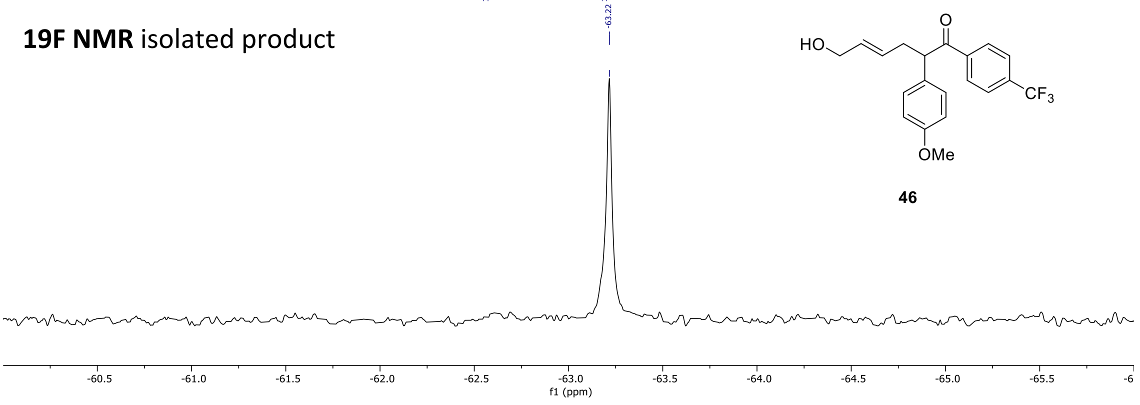

COSY

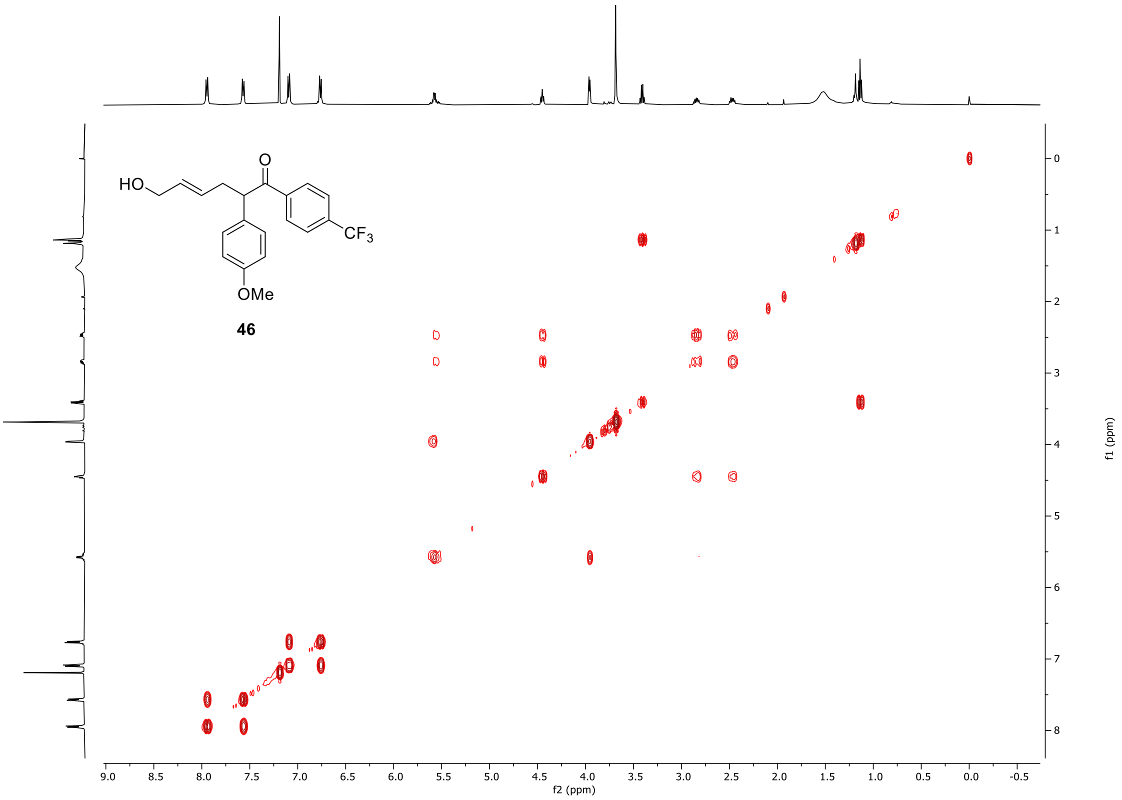

NOESY

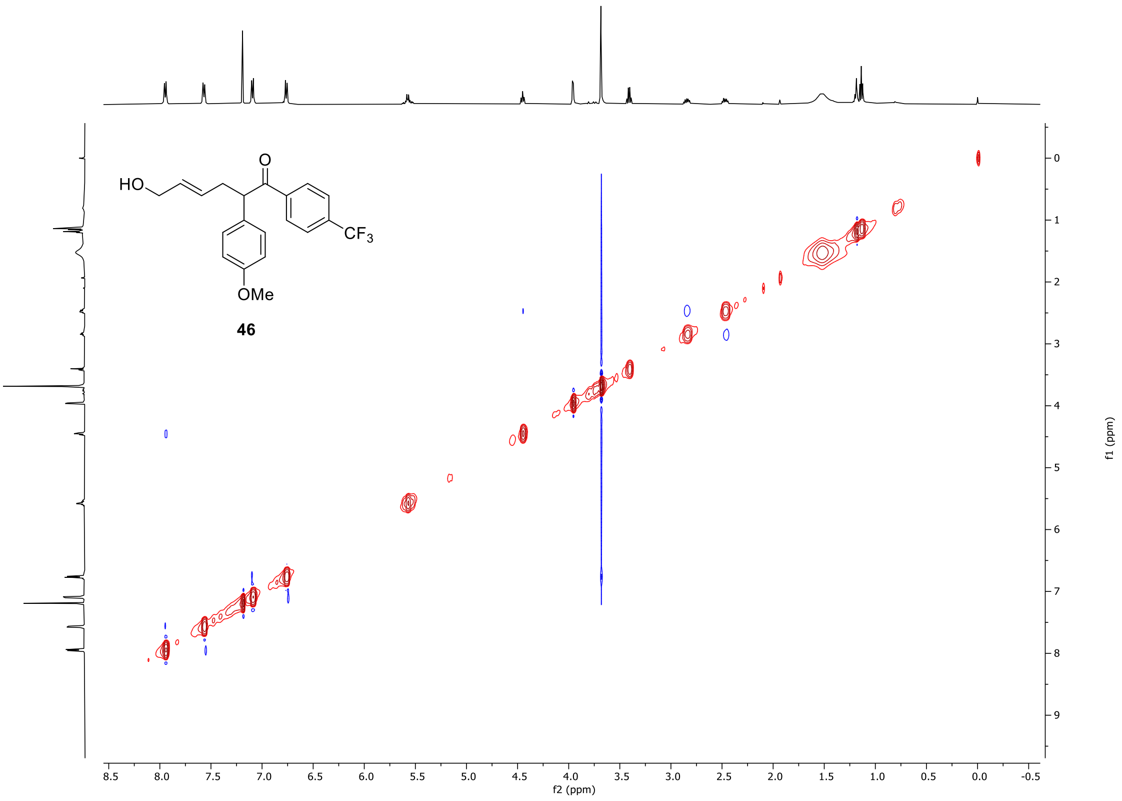

HSQC

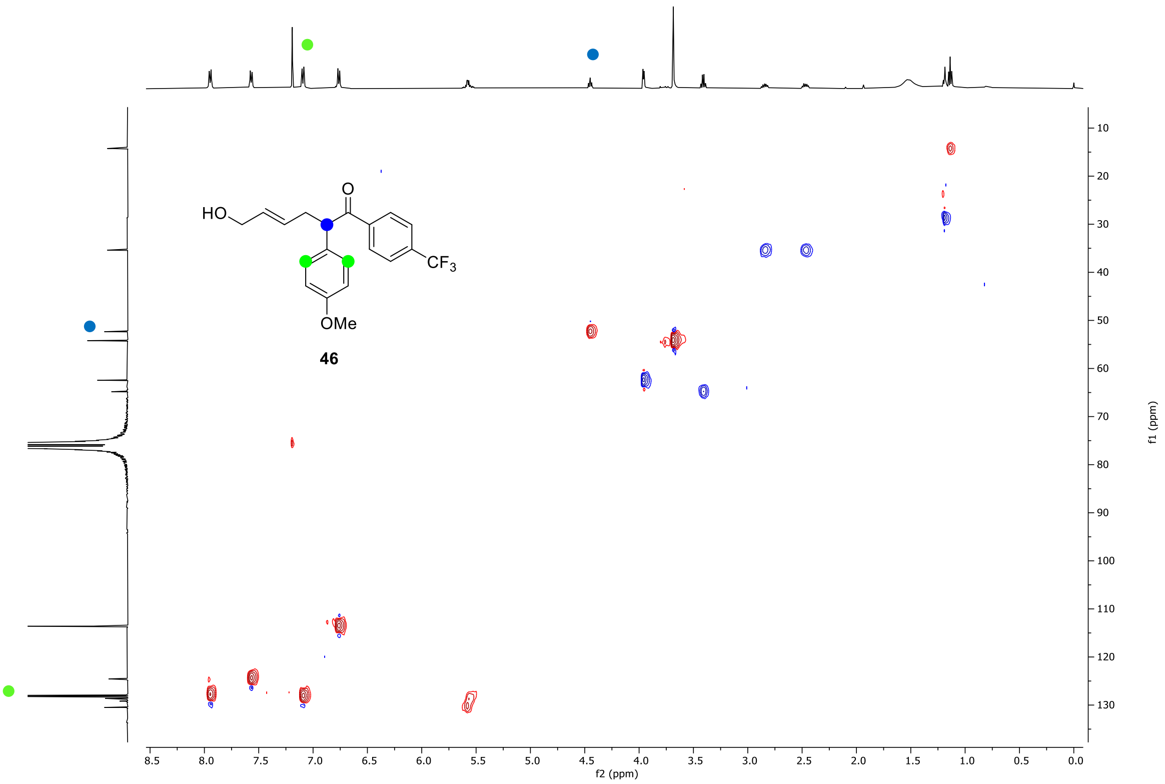

HMBC

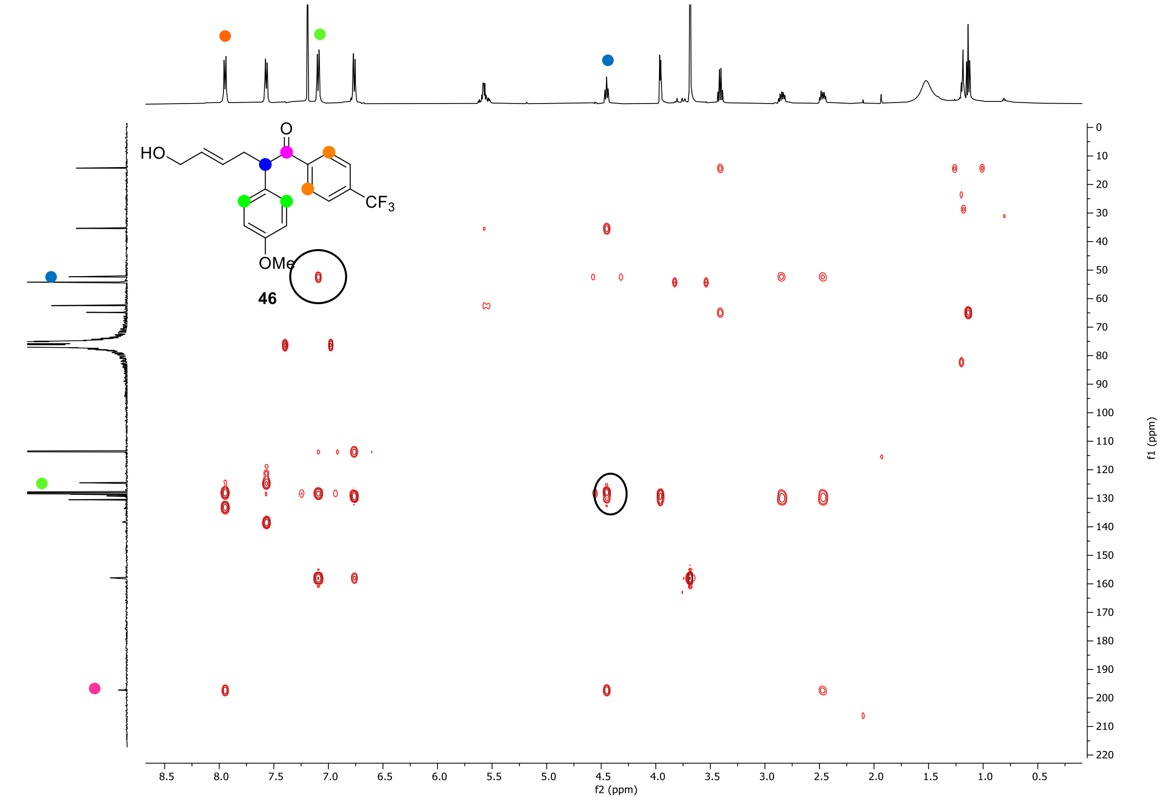

11. References

[1] M. Miio, L. C. Kopel, J.B. Braun, T. L. Gadzikwa, K. L. Hull, R. G. Brisbois, C. J. Markworth, P. A. Grieco, *Org. Lett*. **2002**, 4, 3199.

[2] Y. Yoshida, E. Takesh, *Polym. Chem*., **2016**, *7*, 6770.

[3] S. F. Ken, E. F. Thorsten, *Tetrahedron* **1989**, *45*, 2969.

[4] L. Deng, A. W. Kleij, W. Yang, *Chem. Eur. J*. **2018**, *24*, 19156

[5] E. Hansen, D. Lee, *J. Am. Chem. Soc*. **2006**, *128*, 8142.

[6] W. Su, T.-J. Gong, Q. Zhang, Q. Zhang, B. Xiao, Y. Fu, *ACS Catal*. **2016**, *6*, 6417
